# You Had Me at “MAGIC”!: Four Barley MAGIC Populations Reveal Novel Resistance QTL for Powdery Mildew

**DOI:** 10.3390/genes11121512

**Published:** 2020-12-18

**Authors:** Fluturë Novakazi, Lene Krusell, Jens Due Jensen, Jihad Orabi, Ahmed Jahoor, Therése Bengtsson

**Affiliations:** 1 Department of Plant Breeding, Swedish University of Agricultural Sciences, P.O. Box 101, 23053 Alnarp, Sweden; fluture.novakazi@slu.se (F.N.); ahja@nordicseed.com (A.J.); 2 Sejet Plant Breeding, Nørremarksvej 67, 8700 Horsens, Denmark; lkr@sejet.dk; 3 Nordic Seed A/S, Kornmarken 1, 8464 Galten, Denmark; jdje@nordicseed.com (J.D.J.); jior@nordicseed.com (J.O.)

**Keywords:** *Blumeria graminis* f. sp. *hordei*, GWAS, *Hordeum vulgare* L., linkage disequilibrium, multi-locus mixed linear model, multi-parent advanced generation inter-cross, plant breeding

## Abstract

*Blumeria graminis* f. sp. *hordei* (*Bgh*), the causal agent of barley powdery mildew (PM), is one of the most important barley leaf diseases and is prevalent in most barley growing regions. Infection decreases grain quality and yields on average by 30%. Multi-parent advanced generation inter-cross (MAGIC) populations combine the advantages of bi-parental and association panels and offer the opportunity to incorporate exotic alleles into adapted material. Here, four barley MAGIC populations consisting of six to eight founders were tested for PM resistance in field trials in Denmark. Principle component and STRUCTURE analysis showed the populations were unstructured and genome-wide linkage disequilibrium (LD) decay varied between 14 and 38 Mbp. Genome-wide association studies (GWAS) identified 11 regions associated with PM resistance located on chromosomes 1H, 2H, 3H, 4H, 5H and 7H, of which three regions are putatively novel resistance quantitative trait locus/loci (QTL). For all regions high-confidence candidate genes were identified that are predicted to be involved in pathogen defense. Haplotype analysis of the significant SNPs revealed new allele combinations not present in the founders and associated with high resistance levels.

## 1. Introduction

Cultivated barley (*Hordeum vulgare* ssp. *vulgare* L.) is mainly grown for fodder and brewing purposes [1], and has the advantage that it can be cultivated under harsh conditions and at high altitudes [1,2]. Nevertheless, pests and diseases still pose a threat. One of the most wide-spread diseases in cereals is powdery mildew (PM) caused by the obligate Ascomycota pathogen *Bgh* [3]. In Europe, it is a disease that can lead to yield losses of up to 30% as well as reduced grain quality [4]. *Bgh* epidemics are currently controlled by chemicals and by breeding for resistant barley cultivars, where the latter constitutes the most economically and environmentally sound method. Several important resistance (*R*) genes in barley have been described, and are used by barley breeders to develop resistant cultivars. These are *Mla*, *Mlat*, *MlGa*, *Mlk*, *Mlnn*, *Mlra* on chromosome 1H [5,6,7], *MlLa* and *MlMor* on chromosome 2H [7,8,9], *Mlg* and *MlBo* on chromosome 4H [7,10], *Mlj* on chromosome 5H [11], *Mlh* on chromosome 6H [7], *mlt* and *Mlf* on chromosome 7H [11] and many more, some of which are derived from wild (*H. v.* ssp. *spontaneum*) and bulbous barley (*H. bulbosum*) [12]. All these *R* genes have in common that they are major, race-specific genes, and therefore, prone to be easily overcome by the pathogen within a few years and not considered durable [12].

One major race-specific resistance locus is the gene cluster *Mla* on chromosome 1H [7]. The *Mla* locus shows suppressed recombination but high rates of polymorphism that are based on point mutations and indels [6,13,14]. This gene has over 30 described specificities [15,16,17,18] and was mapped to the telomeric end of chromosome 1HS [6]. Wei et al. [6] showed that the *Mla* specificities *Mla6*, *Mla13*, *Mla14* and *Ml-Ru3* co-segregate and narrowed the interval down to a 240 kb region. They identified eight coiled-coil nucleotide-binding-site leucine-rich-repeats (CC-NBS-LRR) resistance gene homologues located in the *Mla* region that cluster into three families (*RGH1*, *RGH2*, *RGH3*) [6,19]. Six *Mla* specificities have been cloned, *Mla1*, *Mla6*, *Mla7*, *Mla10*, *Mla12* and *Mla13* and it was shown that some *Mla* specificities require additional genes in order to express full resistance, such as *Rar1* (*required for* Mla*-specified resistance 1*) [20,21,22,23,24].

The big exception to race-specific genes is the *Mildew resistance locus o* (*Mlo*) [25]. It was first described by Freisleben and Lein [26], who X-rayed the barley cultivar ‘Haisa’ and identified three genotypes that were completely resistant against three *Bgh* isolates. After that, many more mutants were induced in various genetic backgrounds [27] and to date more than 40 *mlo* alleles are known [28]. Additionally, in the 1970s it was confirmed that the mutation can also occur spontaneously, after it was found in Ethiopian landrace collections from the 1930s [27]. Through a loss-of-function mutation, *mlo* plants inherit resistance against a wide range of PM isolates by formation of cell wall appositions that prevent the fungus from penetrating the epidermal cell walls [25]. However, in order to function *mlo* requires two genes, *Ror1* and *Ror2*, *required for* mlo*-specified resistance* [29]. The *mlo* gene is recessively inherited and located in the middle of the long arm of chromosome 4H [25,27]. The coded protein is a seven transmembrane protein with an extracellular N-terminal segment and an intracellular C-terminal domain, has a highly conserved gene structure and shows a monophyletic origin that is restricted to the plant kingdom [30,31]. Since its discovery, *mlo* has been widely used in resistance breeding against PM and still shows durability in the field [32]. Despite its success story, *mlo* has several drawbacks. It shows a pleiotropic effect, i.e., necrotic leaf lesions occur spontaneously, which lead to a decrease in kernel size and yield due to reduced photosynthesis [32]. However, the biggest drawback is the trade-off between PM resistance and resistance towards non-biotrophic pathogens. Barley plants carrying *mlo* resistance show enhanced susceptibility towards diseases like the rice blast pathogen *Magnaporthe grisea* [33], spot blotch disease caused by *Bipolaris sorokiniana* [34] and Ramularia leaf spot caused by *Ramularia collo-cygni* [35]. Especially, Ramularia leaf spot epidemics have increased in the past decades in all major barley growing regions and the disease has become a major threat for barley production [36]. This emphasizes the need for the continuous search for novel sources of resistance from diverse barley germplasm, as well as, the need to pyramid resistance genes against different diseases into adapted and semi-adapted genetic backgrounds.

One powerful method to detect QTL (quantitative trait locus/loci) associated with traits of interest is genome-wide association studies (GWAS) [37]. An advantage of GWAS is that unrelated populations can be analysed, thereby increasing the number of recombination events and allelic diversity that can be exploited and at the same time increasing the mapping resolution [38]. In contrast, in QTL mapping, bi-parental populations are prerequisite limiting the number of analysed alleles to two per locus [39]. Nevertheless, this also comes with the disadvantage that unrelated or uncontrolled populations often have more complex population structures that will hamper true allelic effects [40]. Additionally, markers with minor allele frequency (MAF) < 1% and low frequency 1%  ≤  MAF  <  5% are excluded from the analysis, in order to account for possible false positive associations, resulting in low power for detecting rare alleles [40].

Multi-parent advanced generation inter-cross (MAGIC) populations for GWAS can be considered the golden mean between unrelated and bi-parental populations, as they combine the advantages of both [41,42]. MAGIC populations typically consist of 4, 8 or 16 founders (parents), which are inter-crossed in funnels for several generations, followed by inter-crossing of individuals from different funnels and subsequently development recombinant inbred line (RIL) or doubled haploid (DH) production [41,42]. Since MAGIC populations are derived from only a few founders allele frequencies are much higher than in unrelated populations, thus, increasing the chance of detecting rare alleles [42]. By inter-crossing all founders with each other, genetic recombination and variation as well as the number of polymorphisms is increased, while linkage disequilibrium (LD) decay is decreased, allowing for higher mapping precision and resolution compared to bi-parental populations. Additionally, inter-crossing can yield individuals with novel allele combinations that are not present in the parent lines [41]. If the founders are carefully chosen, the phenotypic and genotypic diversity can be increased for the traits of interest compared to bi-parental populations [41]. Moreover, pyramiding of genes for traits of interest can be done while developing MAGIC populations without the need for backcrossing [42]. If the selected founders consist of adapted material, the produced RILs can be used as breeding lines or released directly as cultivars [41,42]. Finally, RILs can be used to develop near isogenic lines (NILs) which can further be used for fine mapping [42]. Several examples of QTL detection using MAGICs have been reported in recent years spanning over a variety of plant species including crops such as cotton [43], sorghum [44], *Brassica juncea* [45], rice [46,47,48,49], wheat [50,51,52,53], maize [54], and barley [55].

The most commonly used GWAS model, for both quantitative and qualitative traits, is the single-locus model. However, the single-locus model disregards the presence of multiple QTL, which may lead to less statistical power, biased effect estimates and increased Type I and Type II errors [56]. Consequently, a number of multi-locus models have been suggested that may increase the power in QTL detection [56].

Considering the advantages of MAGICs, four Nordic spring barley MAGIC populations have been developed, pyramiding resistance towards diseases such as leaf scald (LS), net type net blotch (NTNB), fusarium head blight (FHB), leaf rust (LR), and PM. These MAGIC populations were evaluated for PM resistance under field conditions, at two locations in Denmark, in 2017 and 2018. Eight founders were used for each of the four populations, selected based on previous knowledge of their disease resistance and yield performance. The founders included in this study consist of breeding lines, cultivars and landraces. Landraces harbor valuable sources for tolerance and resistance to both biotic and abiotic stress. For instance, earlier screenings of landraces have led to the detection of novel sources of resistance to PM [9,57,58,59,60,61].

Here we report of three putatively new QTL located on chromosomes 1HL, 4HS and 5HS and allele combinations associated with PM resistance in Nordic spring barley, detected using a multi-locus genome-wide association approach in GAPIT (Genome Association and Prediction Integrated Tool). Data for PM resistance of 474 Doubled haploid (DH) lines, i.e., MAGIC progenies was collected from three environments in Denmark.

## 2. Materials and Methods

### 2.1. Multi-Parent Advanced Generation Inter-Cross (MAGIC) Populations

**Figure 1:**
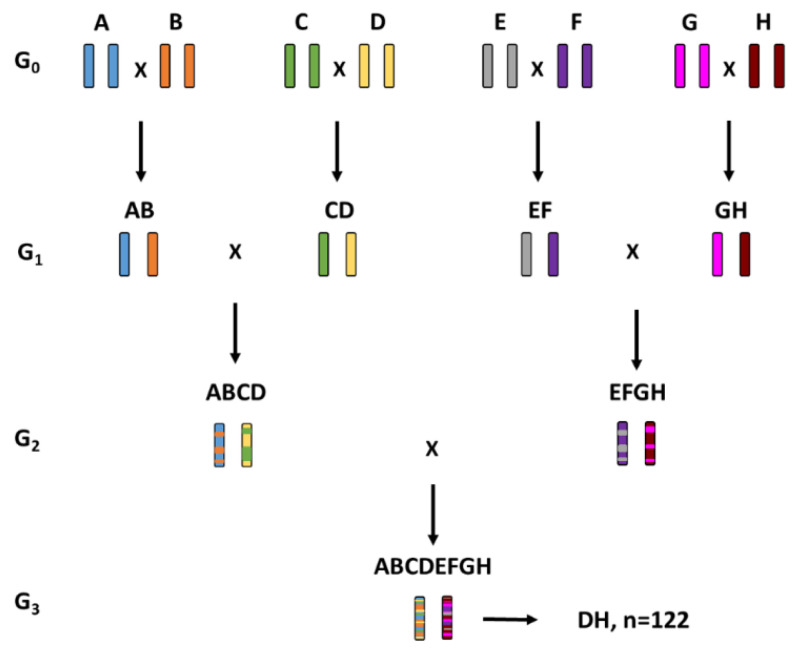
Crossing scheme of the eight-founder barley MAGIC populations, exemplary for MAGIC 1. Capital letters correspond to A: Ylitornion, B: GN 06075, C: Lavrans, D: RGT Planet, E: MBR 1012, F: Iron, G: JLB06-034. The name of each generation (G) can be found on the left side of the scheme indicated by a G followed by a lower-case number. DH means doubled haploid and n indicates the number of DH lines produced.

A barley panel of 490 genotypes, consisting of progenies and founders from four multi-parent populations, was investigated in this study. The founders consisted of cultivars (9), breeding lines (4) and landraces (4) (Table 1) with desirable traits for diseases such as scald, leaf rust, Fusarium and spot form of net blotch and PM [62]. One goal was to pyramid resistances into adapted background. Therefore, cultivars such as RGT Planet and SJ 11198 were included as founders. These lines have the mlo-11 allele, as well as other desirable agronomic traits such as yield and adaptation. The MAGIC populations were developed, using half diallel mating of eight founders (Figure 1). Doubled Haploid (DH) lines were developed from the G3 progenies at Sejet Plant Breeding, Boreal Ltd. and Nordic Seed. MAGIC 1 yielded n = 122 progenies, MAGIC 2 n = 29, MAGIC 3 n = 81 and MAGIC 4 yielded n = 303 progenies (Table 2). Since only a low number of progenies could be developed for MAGIC 2 and seven of eight founders were the same for MAGIC 1 and MAGIC 2, these two populations were treated as one in all subsequent analyses. All analyses were performed for each population separately, i.e., MAGIC 1 + 2, MAGIC 3 and MAGIC 4, and across populations.

### 2.2. Field Trials and Phenotypic Evaluation

Field trials were conducted at two locations in Denmark, Dyngby (2017, 2018) and Horsens (2018) (Table 3). They were set up in an α lattice design with one and two replications in Horsens and Dyngby, respectively. Since PM is ubiquitous, trials rely on natural inoculation. The disease assessment was conducted when pressure on susceptible founder lines like ‘Fairytale’ was optimal. Disease severity was observed at two time-points at each location, except in Dyngby 2018, where only one observation was performed due to serious drought and consequently low disease severity.

Powdery mildew severity was assessed using a rating scale from 1–9, where 1 represents no infection and 9 high susceptibility.

### 2.3. Statistical Analysis

Descriptive statistics were calculated for each population for all observations separately and combined, using the *psych* software package v. 1.8.12 [63] in R [64]. The frequency distribution was calculated in R for each population using the mean values of each line across environments. The pairwise Spearman Rank correlations were calculated in R using mean values from replicates of each line, observation time, field location, and year, respectively.

Analysis of variance (ANOVA model III, with Satterthwaite’s method) were performed using the lmer function in the *lme4* R package [65], to estimate the relative contributions of genotype, environment, and genotype by environment interactions. The model assumed the genotype, environment, and genotype by environment effect to be fixed, and the observations nested within the environments to be random.

The best linear unbiased predictors (BLUPs) for PM were calculated across the three environments for each population, using the lme function in the *nlme* R package [66], assuming all effects to be random. Phenotypic data across the three environments were estimated as

*y*_*ijk*_ = *µ* + *g*_*i*_ + *en*_*j*_ + *r*_*(j)k*_ + *e*_*ijk*_ (1)

where *y_ijk_* is the *k*th observation of the *i*th genotype in the *j*th environment, *μ* is the common intercept, *g_i_* is the random effect of the *i*th genotype, *en_j_* is the effect of the *j*th environment, *r_(j)k_* is the effect of the *k*th observation in environment *j*, and *e_ijk_* is the corresponding error. The BLUPs for each population and across populations were then used as phenotype values for the association mapping.

The broad sense heritability (*H*^2^) was estimated as:

(2) $$H^{2}\;=\;\frac{V_{G}}{V_{G}\;+\;\frac{V_{GE}}{e}+\;\frac{V_{R}}{eo}}$$

where *V_G_* is genotypic variance component, *V_GE_* is variance component of genotype x environment, *V_R_* is residual variance component, and *e* and *o* are the numbers of environments and observations, respectively.

### 2.4. Genotyping and SNP Filtering

The barley panel was genotyped with the SNP&SEQ Technology Platform, Uppsala (www.genotyping.se) using the 50K Illumina Infinium iSelect genotyping array for barley with 44 040 working SNP assays [67]. Leaf samples from seedlings were freeze-dried and homogenized prior to DNA extractions, using a QIAcube HT extraction and the QIAamp 96 DNA QIAcube HT Kit (Qiagen, Hilden, Germany), as previously described in Åhman and Bengtsson [68].

Prior to subsequent analyses, all heterozygote calls were set to missing, the SNPs were filtered for call rate (≥95%) and monomorphic and unmapped markers were removed. This resulted in 27 407 SNPs for MAGIC 1 + 2, 24 093 SNPs for MAGIC 3 and 24 997 SNPs for MAGIC 4 and 31 667 SNPs for the whole panel for analyses. For GWAS the SNPs were further filtered for MAF ≤ 0.05, resulting in 25 068 polymorphic SNPs for MAGIC 1 + 2, 18 103 SNPs for MAGIC 3, 19 072 SNPs for MAGIC 4 and 24 638 polymorphic SNPs for the whole panel to be used in the association mapping. The physical positions based on the barley reference genome [67,69] were retrieved using the online tool BARLEYMAP (http://floresta.eead.csic.es/barleymap/) [70].

### 2.5. Population Structure and LINKAGE Disequilibrium

Population structure was determined based on a Bayesian clustering approach using STRUCTURE v. 2.3.4 [71]. STRUCTURE was run ten times for each hypothetical number of sub-groups (K), between one and ten. The ploidy level was set to 2 and the Markov Chain Monte Carlo (MCMC) was set to 5000 burn-in phases and 10 000 NUMREPS. The most likely number of sub-groups (K) were determined based on the Delta K method [72] using Structure Harvester v0.6.94 [73]. STRUCTURE analysis was performed for each population individually and for the whole panel.

Principal component analysis (PCA) was performed using the build-in function *prcomp* in R v 4.0.2 and plotted with the package *ggfortify*.

Linkage disequilibrium (LD) was estimated using the function *LD.decay* from the package *sommer* v 2.9 in R v 4.0.2 [74] by calculating the squared allele frequency correlation *r^2^* between marker pairs. Markers with a minor allele frequency (MAF) below 0.05 were excluded. The intra-chromosomal LD decay was calculated by plotting *r^2^* values against the physical distance with a second-degree smoothed loess curve fitted using the build-in R function *loess* with span set to 0.1. The 95th percentile of the LD distribution between unlinked markers was calculated and considered as threshold when estimating LD decay [55]. LD decay was estimated for each chromosome per population, genome-wide per population and genome-wide for the whole panel.

### 2.6. Association Mapping

GWAS were performed using four models: general linear model (GLM), mixed linear model (MLM) [75], multiple loci mixed linear model (MLMM) [76] and fixed and random model circulating probability unification (FarmCPU) [77] using GAPIT [78]. To find the best model–covariate combination to account for population structure, the kinship matrix (K) calculated in GAPIT with the Van Raden method [79], the ancestry coefficient data (Q matrix) obtained from STRUCTURE and the principal component analysis (PCA) covariates from GAPIT, were incorporated into the models. The different models were compared, where possible, based on (i) the least deviation from the expected *p*-values, (ii) highest number of groups, (iii) high −2 log likelihood value (–2LL) and (iv) lowest variance error. Manhattan plots were generated with the R package *CMPlot*.

The Bonferroni threshold for significant associations was calculated based on the number of effective markers (MAGIC 1 + 2 n = 4226, MAGIC 3 n = 1618, MAGIC 4 n = 1999, MAGIC 1 to 4 n = 4923) with α = 0.05 [80].

### 2.7. Candidate Gene Indentification and Haplotype Formation

Candidate genes, their locations and annotations were retrieved from the BARLEYMAP website [70] (http://floresta.eead.csic.es/barleymap/). The gene search around the peak markers was increased according to the LD decay of the respective chromosome.

Haplotypes were constructed for each population based on the respective significant markers.

## 3. Results

### 3.1. Panel Evaluation

All data concerning analysis of phenotypic data are found in Figure S1 and Table S1. Phenotypic results showed a wide range of variability for mildew severity in all four panels. The frequency distribution for MAGIC 1 + 2 was right skewed with a mean infection severity of 3.11 and over 70 lines showing infection scores between 1 and 3. MAGIC 3 showed an almost normal distribution with a mean infection severity of 4.03. Frequency distribution for MAGIC 4 was slightly right skewed with a mean infection severity of 4.04 and about 50 lines showing infection scores <3. The frequency distribution across all environments and panels (MAGIC 1 to 4) was slightly right skewed with a mean infection severity of 3.80. The broad sense heritability was very high for all populations as well as for the combined panel (MAGIC 1 to 4) and ranged from *H*^2^ = 0.96 to *H*^2^ = 0.98 (Figure S1). Significant correlations (*p* ≤ 0.05) were found between all observations per panel. The observations Horsens_18_1 and Horsens_18_2 showed the lowest correlation in all panels (r_s_ ≈ 0.2). Furthermore, Horsens_18_1 showed generally low correlation with Dyngby_17_1 and Dungby_17_2 in all panels. All other observation combinations showed moderate to high correlations (0.4 and above) (Figure S1).

Analysis of variance (ANOVA) revealed that in all populations the genotype had a significant effect (*p* < 0.0001), whereas no significance was observed for the environment or the genotype by environment interaction (Table S1).

### 3.2. Population Structure and Linkage Disequilibrium

**Figure 2:**
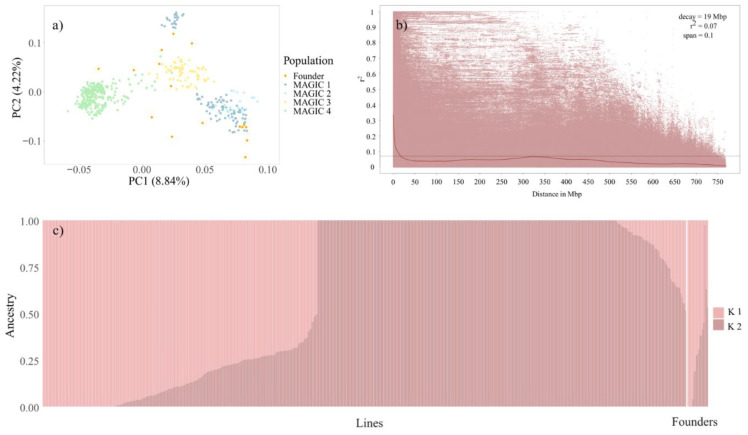
Analyses of population structure and linkage disequilibrium (LD) decay for the combined panel, MAGIC 1 to 4. (**a**) Principle component analysis (PCA) plot of the first two components, colored according to population; (**b**) genome-wide LD decay plot, with r^2^ plotted against the physical position (Mbp), the horizontal line represents the LD decay threshold (**c**) Population structure revealed by STRUCTURE analysis.

STRUCTURE analysis identified an optimal *k* value of 2 for each population tested (Figure S2). In the case of MAGIC 1 + 2, MAGIC 3 and MAGIC 4 the identification of two sub-populations can be ascribed to the fact that the STRUCTURE software assumes at least two sub-populations. For MAGIC 1 + 2 n = 24 individuals grouped to K1 and n = 100 to K2, with n = 10 individuals showing admixed ancestry (<0.75). For MAGIC 3 n = 16 individuals grouped to K1, n = 31 to K2 and n = 36 were admixed. K2 included all founders of MAGIC 3. For MAGIC 4 n = 120 individuals grouped to K1, n = 100 and n = 59 were admixed. All founders of MAGIC 4 showed admixed ancestry. In the combined panel (MAGIC 1 to 4) STRUCTURE analysis revealed as well an optimal *k* = 2 (Figure 2c). One hundred and fifty-four (154) individuals grouped to K1, n = 265 grouped to K2 and n = 71 showed admixed ancestry. Of the 265 individuals belonging to sub-population K2, 257 were lines from MAGIC 4. The remaining eight lines were from MAGIC 1 and the founder ‘Fairytale’. Principle component analysis (PCA) showed all individual populations are unstructured and do not cluster according to row-type. The first two components explained 17.96%, 14.84% and 13.46% of the phenotypic variation for MAGIC 1 + 2, MAGIC 3 and MAGIC 4, respectively. (Figure S2). PCA for the complete panel (MAGIC 1 to 4) showed most lines belonging to MAGIC 4, seven lines belonging to MAGIC 1 and one parent forming one cluster and all other lines forming another (Figure 2a), thereby, confirming STRUCTURE analysis results. Nonetheless, it is a weak population structure, where the first two components explain 13.06%.

LD decay in MAGIC 1 + 2 varied from 7 Mbp (3H) to 19 Mbp (7H) per chromosome and was estimated at 14 Mbp across chromosomes (Table 4, Figure S3). Genome-wide LD decay for MAGIC 3 dropped at 38 Mbp and varied from 31 Mbp (5H) to 140 Mbp (6H) per chromosome (Table 4, Figure S3). MAGIC 4 showed a genome-wide LD decay of 33 Mbp and chromosome-wide LD decays of 28 Mbp (3H) to 497 Mbp (7H) (Table 4, Figure S3). The genome-wide LD decay across all populations was estimated at 19 Mbp (Figure 2b). The LD decay per chromosome varied from 15 Mbp (3H) to 32 Mbp (7H) (Table 4, Figure S3).

### 3.3. Model Selection

**Figure 3:**
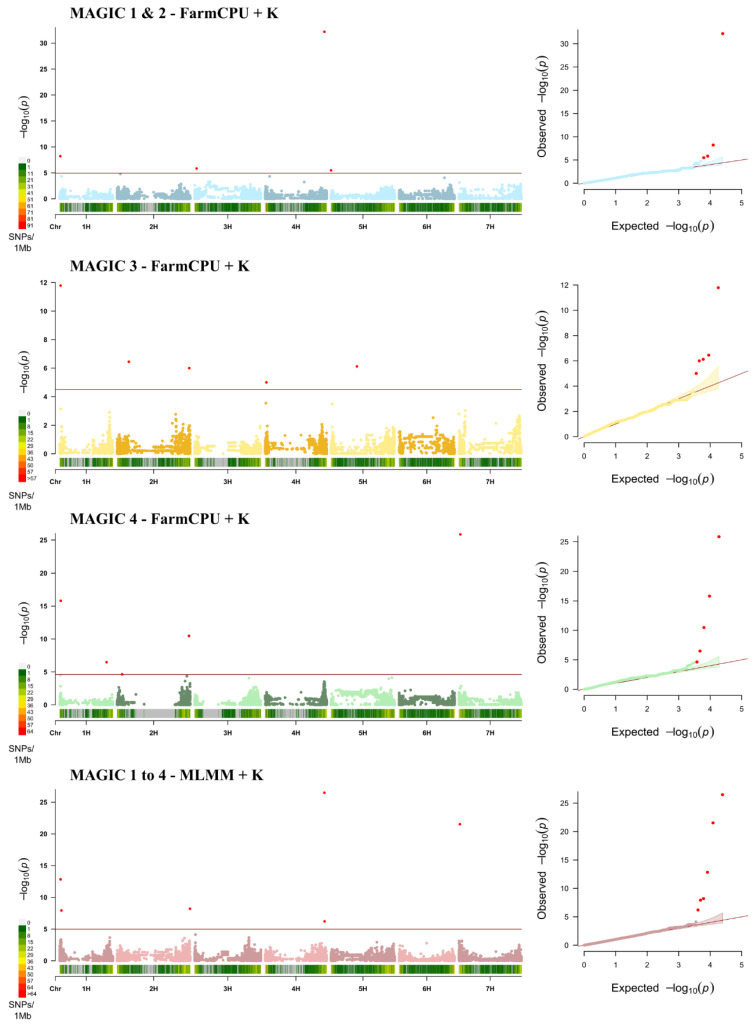
Manhattan plots of the best model and respective quantile–quantile (QQ) plots for each spring barley MAGIC population. The *x*-axis shows the seven barley chromosomes with physical positions, the *y*-axis displays the −log10 (*p*)-values. The red horizontal line represents the Bonferroni adjusted significance threshold –log10 (*p*) with values of 4.93 (MAGIC 1 + 2), 5.1 (MAGIC 3), 4.6 (MAGIC 4), and 4.99 (MAGIC 1 to 4), corresponding to an experiment wise error rate of 0.05.

Based on the Bayesian information criterion (BIC) and maximum log likelihood values, implemented in the model selection option in GAPIT, no principal component was included in the GWAS. Based on the model selection criteria, the best model for MAGIC 1 + 2, MAGIC 3 and MAGIC 4 was FarmCPU including only the kinship (K) (Figure 3, Figure S4). The best model for the combined panel was MLMM + K.

### 3.4. Marker Trait Associations (MTAs)

The output from the GWAS based on the optimal model for each population can be found in Table S2, the corresponding Manhattan plots are shown in Figure 3.

A total of 20 MTAs were detected, corresponding to 11 distinct loci, located on chromosomes 1H, 2H, 3H, 4H, 5H and 7H (Table 5, Figure 3). The first QTL, Qrbg_1H_1, is located on chromosomes 1H between 6.9 and 10.2 Mbp and was detected in all populations. LOD values ranged from 7.9 (JHI_Hv50k_2016_14683) to 15.8 (SCRI_RS_148733) (Table 5). Close to the first QTL a second QTL, Qrbg_1H_2 was detected, located at 18.4 Mbp. This MTA was significant in MAGIC 1 to 4 (Table 5). The third QTL on chromosome 1H, designated Qrbg_1H_3, is located at 492 Mbp and was detected for MAGIC 4 only, with a LOD value of 6.5. Qrbg_2H_1 was detected in MAGIC 3 and MAGIC 4 and the peak markers are located on chromosome 2H at 52.0 and 122 Mbp (Table 5). The second region on chromosome 2H, Qrbg_2H_2, is located at 754 to 765 Mbp and was detected in MAGIC 3, MAGIC 4 and MAGIC 1 to 4, their LOD values ranged from 5.9 to 10.5 (Table 5). Qrbg_3H_1 and Qrbg_4H_1 were identified in MAGIC 1 + 2 and MAGIC 3, respectively, and are located on chromosome 3H at 21 Mbp and on chromosome 4H at 12.2 Mbp. Qrbg_4H_2 was identified for MAGIC 1 + 2 and MAGIC 1 to 4 and is located on the long arm of chromosome 4H at 621 to 623 Mbp. The peak marker JHI_Hv50k_2016_265870 was significant in both populations and had LOD values of 32.2 and 26.5 (Figure 3, Table 5). On chromosome 5H two QTL were identified (Table 5). The first designated Qrbg_5H_1 is located at 396 kbp and was significant in MAGIC 1 + 2. The second QTL, Qrbg_5H_1, was identified in MAGIC 3 and is located at 272 Mbp. The last region, Qrbg_7H_1, is located on chromosome 7H and was significant in MAGIC 4 and MAGIC 1 to 4 (Table 5). The peak markers JHI_Hv50k_2016_444783 and JHI_Hv50k_2016_449746 are located at 9.4 (LOD 21.5) and 14.7 Mbp (LOD 25.9), respectively.

The *p*-value for the significance threshold for MAGIC 1 + 2, MAGIC 3, MAGIC 4 and MAGIC 1 to 4 resulted in logarithm of odds (LOD) values of 4.93, 4.51, 4.6 and 4.99, respectively.

MAGIC 3 showed the highest mean MAF with 0.29 (Table S2). MAGIC 1 + 2 and MAGIC 4 had mean MAF of 0.26 and 0.27, respectively. In the combined panel (MAGIC 1 to 4), the mean MAF was lowest with 0.25.

### 3.5. Candidate Genes for the Identified QTL

The number of annotated genes located in the 11 QTL associated with PM resistance varied between 119 (Qrbg_5H_1) and 883 (Qrbg_7H_1) (Table S3). All regions contained genes that are directly involved in plant defense. In all regions, at least one leucine-rich repeat (LRR) is located and in all regions, except Qrbg_5H_2, at least one disease resistance protein is located. In many regions, peroxidases, pectinases, chitinases, cellulose and callose synthases are found (Table S3). The peak markers of Qrbg_4H_2 are located close to the MLO protein.

### 3.6. Allele Combinations

In order to find lines with favourable allele combinations, haplotypes were formed based on the significant markers for each population. Table 5 shows, which founder contributed the positive allele for each QTL and how many lines carry this allele. Haplotype formation for MAGIC 1 + 2 revealed 14 different haplotypes (Table S4). Five lines had all four positive alleles (TGAT) and significantly reduced BLUP values. However, all lines (n = 46) having the positive allele for Qrbg_4H_2 showed significantly reduced BLUP values between −1.4 and −1.6 (Table S4). This allele is inherited from founders ‘RGT Planet’ and ‘SJ 111998’ (Table 5). Significant SNPs for MAGIC 3 combined into 21 haplotypes. No lines had all five positive alleles (Table S4). Eleven lines, including the founders ‘Fairytale’, ‘Iron’ and ‘RGT Planet’ combined four positive alleles and had significantly reduced BLUP values ranging between −1.6 and −2.2 (Table S4). The positive allele of Qrbg_5H_2 seemed to have the largest influence on disease reduction. This allele was inherited from founders ‘Brage’, ‘Fairytale’, ‘Iron’ and ‘RGT Planet’ (Table 5).

Five lines including the founder ‘Nordic’ showed all negative alleles (CCCTA) and had significantly increased BLUPs (Table S4). Haplotype formation for MAGIC 4 revealed 22 different haplotypes (Table S4). Five lines combined all five positive alleles (ATGAC) and showed reduced BLUPs of –1.7 (Table S4). Especially lines (*n* = 32) with the positive allele from Qrbg_7H_1 showed high resistance (Table 5, Table S4). This allele comes from founder ‘Gaffelbyg’, who despite having only one positive allele (GCACC) has a BLUP of –1.6 (Table S4). Two and six lines have four and three positive alleles and show the lowest mean BLUPs with –2.2 and –2.0, respectively (Table S4). Thirty lines combined all five negative alleles (GCACT) and had significantly increased BLUP values (Table S4). However, lines (*n* = 208), including the founders ‘Krasnodarskij 35’ and ‘Nordic’, that combined the negative alleles of Qrbg_2H_2 and Qrbg_7H_1 all showed increased BLUPs (Table S4).

## 4. Discussion

MAGIC populations offer the advantages of higher allele frequencies and lower LD decay as compared to bi-parental populations and unstructured populations in contrast to diversity panels [41,42]. In the present study four barley MAGIC populations, were investigated for PM resistance at two locations in Denmark, Dyngby (2017, 2018) and Horsens (2018). The absence of population structure allowed the combining of the four MAGIC populations in the subsequent analysis, in order to increase the population size and therefore the power for detecting QTL for resistance towards PM. The initial goal was to produce at least n = 200 DH lines per MAGIC population. Unfortunately, this was not possible for MAGICs 1, 2 and 3. Successful DH line production is very dependent on the genotype [81]. Hence, in order to avoid the risk of producing low numbers of MAGIC progenies, recombinant inbred lines (RILs) should preferably be produced by the single-seed descent method. Population size is an important factor when it comes to the power to detect QTL, which in return is linked to the frequency of an allele [42]. In bi-parental populations, allele frequencies for segregating QTL are 0.5 and power to detect a QTL is maximized. In diversity panels, rare alleles occur at lower frequencies, which decreases the power to detect these alleles significantly [42]. Generally, in MAGIC populations the number of SNPs with low MAF should be very low and in populations consisting of eight founders, any allele would ideally be present at 12.5% [42]. Founder lines of MAGIC 3 are all cultivars, hence, the higher MAF compared to the other MAGIC populations was expected, since cultivars are generally genetically more similar to each other compared to landraces. Of the 24,093 informative SNPs for MAGIC 3, 27.85% had MAF lower than 0.125 and 24.86% had MAF lower than 0.05. The higher diversity of the founders of MAGIC 1 + 2 is reflected in a lower mean MAF compared to MAGIC 3 and MAGIC 4. In the combined panel (MAGIC 1 to 4), the lowest MAF was observed, which was to be expected, since this panel is the most diverse compared to the separate populations. Ongom and Ejeta (2018) reported a mean MAF of 0.15 in a sorghum MAGIC population. However, this population consists of 19 founders, which explains the lower mean MAF. Nonetheless, the majority of the SNPs in the present study had high MAF and were therefore suitable for GWAS.

LD decay can be a measure of a population’s genetic diversity, i.e., the lower the LD decay, the higher the diversity [82]. MAGIC 1 + 2 had the lowest LD decay per chromosome and genome-wide. Six out of the nine founders of MAGIC 1 + 2 are landraces and breeding lines, which presumable are more diverse than cultivars [83] and resulted in low LD decays compared to the other populations. In contrast, the founders of MAGIC 3 are all cultivars, mainly from Europe, and this population showed the highest LD decays. LD decay of MAGIC 4 ranged between those of MAGIC 1 + 2 and MAGIC 3. The founders of MAGIC 4 consist of one landrace, one breeding line and four cultivars, with the line ‘Fairytale’ having served as a founder three times in the crossing scheme. This could have led to a decreased diversity and an increased LD decay. LD is broken down by recombination [84]. Combining the four MAGIC populations results in a set with an increased number of recombination events, which consequently decreases LD decay. Unfortunately, based on the currently available data it is not possible to compare LD decay with other barley MAGIC populations, since the previously published papers on barley MAGICs were calculated on genetic maps [55].

Several barley resistance genes distributed across all seven chromosomes against PM have been described [12]. The most famous resistance gene against PM is *mlo*. This recessively inherited gene is located distally on the long arm of chromosome 4H [85] and confers complete and broad spectrum resistance against a wide range of *Bgh* isolates [27]. This region was also identified in the present study (Qrbg_4H_2). Two founders, ‘RGT Planet’ and ‘SJ 111998’, carry the *mlo11* allele and this region was significantly associated with PM resistance in GWAS with MAGIC 1 + 2 and MAGIC 1 to 4. Qrbg_4H_2 is located between 621 and 623 Mbp and the MLO protein (HORVU4Hr1G082710) is located between the peak markers. Moreover, haplotypes carrying the positive allele had significantly reduced BLUP values, emphasizing the major effect of *mlo*. Even though ‘RGT Planet’ is also a founder of MAGIC 3, we did not detect Qrbg_4H_2 in this population. This could be due to the small population size of MAGIC 3 and the fact that the peak marker JHI_Hv50k_2016_265870 detected in MAGIC 1 + 2 and MAGIC 1 to 4 had a MAF < 0.05.

The resistance gene cluster *Mla* located on chromosome 1H is another major resistance locus against PM [6]. This gene has been detected in many other studies [86,87,88,89,90,91,92] and was also detected in the present study. The region Qrbg_1H_1 was significant in all populations and is located on chromosome 1HS between 6.9 and 10.2 Mbp. Several LRR and disease resistance proteins are located in this region, as well as one coiled-coil NBS-LRR (HORVU1Hr1G003700) between the two significant markers JHI-Hv50k-2016-7757 (6 976 974 bp) and BOPA2_12_30918 (8 935 905 bp). Only 8 Mbp away from another region Qrbg_1H_2 at 18.3 Mbp was detected. The significant marker JHI_Hv50k_2016_14683 is not in linkage with the markers from region Qrbg_1H_1 and, therefore, represents a distinct QTL. We hypothesize this region to correspond to the resistance locus *Mlk*, which was reported to be located about 7.7 cM from *Mla* [16]. A third QTL, Qrbg_1H_3, was identified on chromosome 1H at 492 Mbp. Based on the available data, no corresponding QTL has to our knowledge been described located in this region, hence, this region can be considered as a putatively novel resistance QTL. Qrbg_1H_3 was only detected in GWAS for MAGIC 4. Nonetheless, several peroxidases, callose and cellulose synthases, chitinases, as well as LRRs and disease resistance proteins are located close to the peak marker. All these proteins are known to be involved in plant defense [93,94,95,96,97], hence, supporting the region to be a true resistance QTL.

On chromosome 2H two SNPs (Qrbg_2H_1) that were significant for GWAS in MAGIC 3 and MAGIC 4, were identified located at 52 and 122 Mbp. Haplotype analysis showed that the SNPs alone had only a minor effect on disease reduction and the positive allele for Qrbg_2H_1 was easily masked when negative alleles were present for the other QTL (Table S4). Nonetheless, close to the peak marker JHI-Hv50k-2016-87627 disease resistance proteins and WRKY transcription factors are located [95]. Several studies have identified QTL for resistance on the short arm of chromosome 2H. Von Korff et al. [91] identified three QTL on chromosome 2H for PM resistance located between 17 and 146 cM, named *lang1031Qrbg.S42-2H.a*, *Qrbg.S42-2H.b*, *Qrbg.S42-2H.c*. Shtaya et al. [98] detected a QTL at 100.5 cM designated *Rbgq1* and Aghnoum et al. [90] detected two QTL, *Rbgq7* and *Rbgq8*, located around 25–40 cM and 50–70 cM, respectively. Schweizer and Stein [99] identified several meta-quantitative trait loci between 0 and 80 cM on chromosome 2HS. Based on the available data it is not possible to say, if these regions and the one identified in the current study correspond to the same QTL, however it is very likely. The second QTL detected on chromosome 2HL is located between 754 to 765 Mbp and was detected in GWAS with MAGIC 3, MAGIC 4 and MAGIC 1 to 4. This region corresponds to the resistance QTL *MlLa* [8,100] and was introgressed from *Hordeum laevigatum* [101]. This gene is known to confer intermediate resistance towards PM and was detected in many studies [8,86,90,91,99]. Hoseinzadeh et al. [102] identified and fine-mapped a resistance QTL derived from the Ethiopian landrace ‘HOR2573’. This QTL mapped to the resistance gene *MlLa* and was, therefore, designated *MlLa-H*. They were able to map the QTL to an 850 kb interval between 762.8 and 763.7 Mbp and identify four leucine-rich repeats as candidate genes [102]. The four candidate genes, HORVU2Hr1G126250, HORVU2Hr1G126380, HORVU2Hr1G126440 and HORVU2Hr1G126510, are located between the two peak markers JHI-Hv50k-2016-142889 (757 Mbp) and JHI-Hv50k-2016-147232 (765 Mbp) in the present study.

Qrbg_3H_1 was detected in MAGIC 1 + 2 located on chromosome 3HS at 21 Mbp. In the proximity of the peak marker BOPA2_12_30192 there is a LRR protein (HORVU3Hr1G010070) and two disease resistance proteins (HORVU3Hr1G010310, HORVU3Hr1G010990). Aghnoum et al. [90] detected a QTL, *Rbgq10*, for PM resistance on chromosome 3H at 11.8 cM that could coincide with the region identified in the present study, further investigation has to be conducted to verify this.

Two putatively new resistance QTL were detected on chromosomes 4HS (Qrbg_4H_1) and 5HS (Qrbg_5H_1), respectively. The QTL on chromosome 4HS was identified in MAGIC 3 and the peak marker JHI-Hv50k-2016-230367 is located at 12.2 Mbp. Several LRRs are located in proximity to the peak marker (Table S3). Resistance genes on chromosome 4H against PM have been described only on the long arm of this chromosome and correspond to the resistance genes *mlo* and *Mlg* [10,25,85,99,103]. To our knowledge, no resistance locus located on the short arm of chromosome 4H has been reported. Similarly, the locus Qrbg_5H_1 identified on chromosome 5HS at 396 kb does not correspond to any previously reported loci on this chromosome [99]. Three disease resistance proteins (HORVU5Hr1G001030, HORVU5Hr1G001060, HORVU5Hr1G001080) are located about 3 Mbp away from the peak marker, and two serine threonine-protein kinases (HORVU5Hr1G000140, HORVU5Hr1G000240) are located within 600 kb from the peak marker (Table S3). Serine threonine-protein kinases have been shown to be involved in plant signaling pathways and in plant defense [104].

Qrbg_5H_2 was identified in GWAS for MAGIC 1 + 2 and is located on chromosome 5H at 272 Mbp. Only one LRR (HORVU5Hr1G037990) is located in proximity to the peak marker, however, other studies have reported resistance QTL for PM in this region before [90,91] and this locus most likely coincides with the semi-dominant resistance gene *Mlj*, derived from *H. vulgare* ssp. *spontaneum*, first reported by Schönfeld et al. [11]. The same study reported another QTL on chromosome 7HS, also derived from *H. vulgare.* ssp. *spontaneum*, designated *mlt* [11]. The *mlt* gene is recessively inherited and confers major race-specific resistance towards PM [11,105]. In a study with a cross between the PM resistant Spanish landrace ‘SBCC97’ and the susceptible cultivar ‘Plaisaint’ a resistance QTL corresponding to *mlt* was detected [58]. In subsequent studies the gene was fine-mapped [106,107]. In the latter study, the authors mapped the locus to a 4 Mbp interval between 9 and 13 Mbp. Amongst others, they identified disease resistance proteins, LRR, and NBS-LRR as putative candidate genes [107]. This QTL coincides with the locus Qrbg_7H_1 (9.4–14.7 Mbp) detected in the present study. Four NBS-LRR and one disease resistance protein are located less than 400 kb away from the peak marker JHI-Hv50k-2016-444783.

Fifteen lines of MAGIC 1 + 2 showed haplotypes that were not found in the founders and had mean BLUPs lower than the best founders. Five lines even have the positive alleles (TGAT) for all four QTL detected in this population. All fifteen lines carry the positive allele for *mlo*, which might have overshadowed the effect of the other QTL. However, the lines carrying all positive alleles show that pyramiding of resistance QTL is possible and these five lines carry the putatively new resistance QTL Qrbg_5H_1. In MAGIC 4, 16 lines had haplotypes not shared with any of the founders and were more resistant than the most resistant founder ‘Gaffelbyg’. Five lines carried all five positive alleles (ATGAC), however, the highest effect on resistance was conferred by QTL Qrbg_1H_1 (*Mla*) and Qrbg_7H_1 (*mlt*). Nonetheless, seven out of the 16 most resistant lines, carried the positive allele for the putatively new locus Qrbg_1H_2 in addition to *Mla* and *mlt* and could be used for further analysis and trait pyramiding. Generally, haplotype analysis showed that MAGIC populations are a good tool for breeding lines with new allele combinations that exceed the founders’ performances, as was proposed in Huang et al. [41].

## 5. Conclusions

In the present study, four barley MAGIC populations were screened for PM resistance under field conditions. Phenotypic analysis showed high variation between the lines and a significant effect of the genotype. Genetic analysis of the populations revealed no population structure and suitable LD decay combined with a high number of informative SNPs, showing high suitability for conducting GWAS. GWAS identified 11 QTL associated with PM resistance. Three out of the 11 QTL are putatively new resistance loci as strongly supported by the identified candidate genes. Additional haplotype analysis revealed lines with new allele combinations and high resistance levels compared to the founders.

Further studies on lines carrying the positive alleles for these putatively new loci with different isolates have to be conducted to test the nature of these putatively new resistance loci and to determine whether they confer race or non-race specific resistance.

## Figures and Tables

**Table 1:** Founders of the four barley MAGIC populations.

Letter	Pedigree	Type	Country of Origin	Row Type
A	Ylitornion	Landrace	Finland	Six-rowed
B	GN 06075	Breeding line	Norway	Six-rowed
C	Lavrans	Cultivar	Norway	Six-rowed
D	RGT Planet	Cultivar	France	Two-rowed
E	MBR 1012	Landrace	Former Yugoslavia	Six-rowed
F	Iron	Cultivar	Poland	Two-rowed
G	JLB06-034	Landrace	Jordan	Two-rowed
H	GN 09096	Breeding line	Norway	Six-rowed
I	SJ 111998	Breeding line	Denmark	Two-rowed
J	Chevron	Cultivar	Switzerland	Six-rowed
K	Olve	Cultivar	Norway	Two-rowed
L	Brage	Cultivar	Norway	Six-rowed
M	Krasnodarskij 35	Cultivar	Soviet Union	Two-rowed
N	Nordic	Cultivar	US	Six-rowed
O	Fairytale	Cultivar	Denmark	Two-rowed
P	GN 09005	Breeding line	Norway	Six-rowed
Q	Gaffelbyg	Landrace	Denmark	Six-rowed

**Table 2:** Crossing scheme for the four barley MAGIC populations.

Population	Crosses	No of Progenies
MAGIC 1	((A × B) × (C × D)) × ((E × F) × (G × H))	122
MAGIC 2	((A × B) × (C × D)) × ((E × F) × (G × I))	29
MAGIC 3	((J × D) × (K × L)) × ((M × F) × (N × O))	81
MAGIC 4	((P × O) × (Q × O)) × ((M × F) × (N × O))	303

Capital letters correspond to: A: Ylitornion, B: GN 06075, C: Lavrans, D: RGT Planet, E: MBR 1012, F: Iron, G: JLB06-034, H: GN 09096, I: SJ 111998, J: Chevron, K: Olve, L: Brage, M: Krasnodarskij 35, N: Nordic, O: Fairytale, P: GN 09005, Q: Gaffelbyg.

**Table 3:** Field evaluation details.

Environment	Location	Entity	Year	Sowing Day	Replications	Observation Day
1	Dyngby	Nordic Seed	2017	8 April	2	1 June and 21 June
2	Dyngby	Nordic Seed	2018	14 April	2	25 June
3	Horsens	Sejet Plant Breeding	2018	13 April	1	25 May and 15 June

**Table 4:** Linkage disequilibrium decay (Mbp) for each of the four barley MAGIC populations.

Chromosome	MAGIC 1 + 2	MAGIC 3	MAGIC 4	MAGIC 1 to 4
1H	15	60	56	17
2H	14	40	23	17
3H	7	44	28	15
4H	12	45	34	16
5H	13	31	29	19
6H	12	140	429	20
7H	19	33	497	32
Genome-wide	14	38	33	19

**Table 5:** Quantitative trait locus/loci (QTL) for powdery mildew resistance identified in genome-wide association studies in four barley MAGIC populations, markers significantly associated with these regions and their physical locations, as well as founders and lines carrying the positive alleles. Complete lists can be found in Tables S2 and S4.

QTL	SNP	Chromosome	Position (bp)	*p*-Value	LOD	MAF	Effect	MAGIC Population	Founder with pos. Allele	Lines with pos. Allele
Qrbg_1H_1	JHI_Hv50k_2016_7757	1H	6,976,974	6.06 × 10^9^	8.218	0.306	−0.376	1 + 2	GN06075, GN09096, Iron, Lavrans	37
	BOPA2_12_30918	1H	8,935,905	1.45 × 10^13^	12.840	0.343	-	1 to 4	Brage, Fairytale, GN06075, Iron, Krasnordarskij 35, Lavrans, SJ111998	315
	JHI_Hv50k_2016_10019	1H	9,240,411	1.63 × 10^12^	11.788	0.313	−0.531	3	Iron	25
	SCRI_RS_148733	1H	10,236,703	1.55 × 10^16^	15.809	0.151	0.429	4	Fairytale, GN09005, Iron, Krasnordarskij 35	233
Qrbg_1H_2	JHI_Hv50k_2016_14683	1H	18,358,190	1.19 × 10^8^	7.926	0.233	-	1 to 4	Chevron, Gaffelbyg, GN06075, GN09096, Iron, Lavrans, Nordic, Ylitornion	101
Qrbg_1H_3	JHI_Hv50k_2016_37800	1H	492,560,309	3.28 × 10^7^	6.484	0.299	−0.169	4	Nordic	82
Qrbg_2H_1	BOPA1_7623_818	2H	52,014,030	2.29 × 10^5^	4.640	0.147	0.204	4	Krasnordarskij 35, Nordic	39
	JHI_Hv50k_2016_87627	2H	122,769,327	3.56 × 10^7^	6.448	0.289	−0.332	3	Fairytale, Iron, Olve, RGT Planet	20
Qrbg_2H_2	JHI_Hv50k_2016_141795	2H	754,879,170	3.34 × 10^11^	10.476	0.206	−0.360	4	Fairytale, GN09005, Iron	57
	JHI_Hv50k_2016_142889	2H	757,857,556	1.00 × 10^6^	5.999	0.349	0.286	3	Fairytale, Iron, Krasnordarskij 35, Olve, RGT Planet	49
	JHI_Hv50k_2016_147232	2H	765,628,800	6.32 × 10^9^	8.200	0.204	-	1 to 4	Fairytale, JLB06-034	98
Qrbg_3H_1	BOPA2_12_30192	3H	21,019,740	1.46 × 10^6^	5.835	0.276	−0.258	1 + 2	GN06075, GN09096, JLB06-034, Lavrans, RGT Planet, SJ111998	91
Qrbg_4H_1	JHI_Hv50k_2016_230367	4H	12,239,110	9.93 × 10^6^	5.003	0.253	−0.285	3	Brage, Chevron, Fairytale, Krasnordarskij 35, RGT Planet	16
Qrbg_4H_2	JHI_Hv50k_2016_265870	4H	621,555,275	6.47 × 10^33^	32.189	0.358	−1.338	1 + 2	RGT Planet, SJ111998	46
				3.24 × 10^27^	26.489	0.102	-	1 to 4	GN09005, RGT Planet, SJ111998	47
	JHI_Hv50k_2016_267153	4H	623,655,675	6.28 × 10^7^	6.202	0.397	-	1 to 4	Gaffelbyg, GN09005, Iron, JLB06-034, RGT Planet, SJ111998, Ylitornion	187
Qrbg_5H_1	JHI_Hv50k_2016_276999	5H	396,682	3.26 × 10^6^	5.487	0.403	−0.316	1 + 2	JLB06-034, RGT Planet, SJ111998	51
Qrbg_5H_2	SCRI_RS_166374	5H	272,385,765	7.58 × 10^7^	6.120	0.289	0.388	3	Brage, Fairytale, Iron, RGT Planet	57
Qrbg_7H_1	JHI_Hv50k_2016_444783	7H	9,411,745	2.97 × 10^22^	21.528	0.065	-	1 to 4	Gaffelbyg	31
	JHI_Hv50k_2016_449746	7H	14,735,678	1.40 × 10^26^	25.855	0.118	−0.832	4	Gaffelbyg	32

## Supplementary Materials

The following are available online at https://www.mdpi.com/2073-4425/11/12/1512/s1, Figure S1: Frequency distribution of phenotypic results and correlation plots for all environments and populations. Figure S2: Principle component analysis and STRUCTURE output for each individual populations. Figure S3: LD decay plots for each individual MAGIC population per chromosome and across chromosomes and for the combined panel (MAGIC 1 to 4) for each chromosome. Figure S4: Manhattan and QQ-plots for each individual MAGIC population and the combined panel (MAGIC 1 to 4) for different models and covariate combinations. Table S1: Descriptive statistics for all environments and populations and Analysis of variance (ANOVA) for all populations. Table S2: Results of genome-wide association studies (GWAS) for each individual population and the respective best model. Table S3: Predicted genes located in the identified QTL regions. Table S4: Haplotype analysis for each individual population based on the respective significant markers.
