# Supplementary material for: You Had Me at “MAGIC”!: Four Barley MAGIC Populations Reveal Novel Resistance QTL for Powdery Mildew
Source: Genes (Basel). 2020 Dec 18;11(12):1512. doi: 10.3390/genes11121512 (PMC7766815; doi:10.3390/genes11121512)
Supplement: Supplementary file 1 [file genes-11-01512-s001.zip › Figure S2.pdf]

Figure S2

You had me at “MAGIC”! Four barley MAGIC populations reveal novel resistance QTL for Powdery Mildew

Fluturë Novakazi [1], Lene Krusell [2], Jens Due Jensen [3], Jihad Orabi [3], The PPP Barley Consortium, Ahmed Jahoor [3], Therése Bengtsson [1*]

[1] Department of Plant Breeding, Swedish University of Agricultural Sciences, Box 101, 230 53, Alnarp Sweden

[2] Sejet Plant Breeding, Nørremarksvej 67, 8700 Horsens, Denmark

[3] Nordic Seed A/S, Kornmarken 1, 8464 Galten Denmark

*Correspondence: Therése Bengtsson: therese.bengtsson@slu.se

Content

Principle Component Analysis for each individual MAGIC population and the complete panel (MAGIC 1 to 4). Coloured according to population (line or founder) and row-type.

STRUCTURE output results for each individual MAGIC population.

The PPP Barley Consortium
Inger Åhman (Department of Plant Breeding, Swedish University of Agricultural Sciences, Box 101, 230 53 Alnarp, Sweden),
Therése Bengtsson (Department of Plant Breeding, Swedish University of Agricultural Sci-ences, Box 101, 230 53 Alnarp, Sweden),
Fluturë Novakazi (Department of Plant Breeding, Swedish University of Agricultural Scienc-es, Box 101, 230 53 Alnarp, Sweden),
Outi Manninen (Boreal Plant Breeding Ltd, Myllytie 10, 31600 Jokioinen, Finland),
Merja Veteläinen (Boreal Plant Breeding Ltd, Myllytie 10, 31600 Jokioinen, Finland),
Mika Isolahti, (Boreal Plant Breeding Ltd, Myllytie 10, 31600 Jokioinen, Finland),
Muath Alsheikh (Graminor A/S, Hommelstadvegen 60, 2322 Ridabu, Norway),
Stein Bergersen (Graminor A/S, Hommelstadvegen 60, 2322 Ridabu, Norway),
Constantin Jansen (Graminor A/S, Hommelstadvegen 60, 2322 Ridabu, Norway),
Susanne Windju (Graminor A/S, Hommelstadvegen 60, 2322 Ridabu, Norway),
Marja Jalli, (Natural Resources Institute Finland (Luke), Tietotie 4, 31600 Jokioinen, Finland),
Juho Hautsalo, (Natural Resources Institute Finland (Luke), Survontie 9, 40500 Jyväskylä, Finland,
Ahmed Jahoor (NordicSeed A/S, Kornmarken 1, 8464 Galten, Denmark) (project leader),
Jens Due Jensen (Nordic Seed A/S, Kornmarken 1, 8464Galten, Denmark),
Jihad Orabi (Nordic Seed A/S, Kornmarken 1, 8464 Galten, Denmark),
Nana Vagndorf Nordestgaard (Nordic Seed A/S, Kornmarken 1, 8464 Galten, Denmark),
Lene Krusell (Sejet Plant Breeding, Nørremarksvej 67, 8700 Horsens, Denmark),
Rasmus Lund Hjortshøj (Sejet Plant Breeding, Nørremarksvej 67, 8700 Horsens, Denmark),
Charlotte Damgård Robertsen, (Sejet Plant Breeding, Nørremarksvej 67, 8700 Horsens, Den-mark),
Magnus Göransson (The Agricultural University of Iceland, Faculty of Land and Animal Re-sources, Hvanneyri, 311 Borgarnes, Iceland)

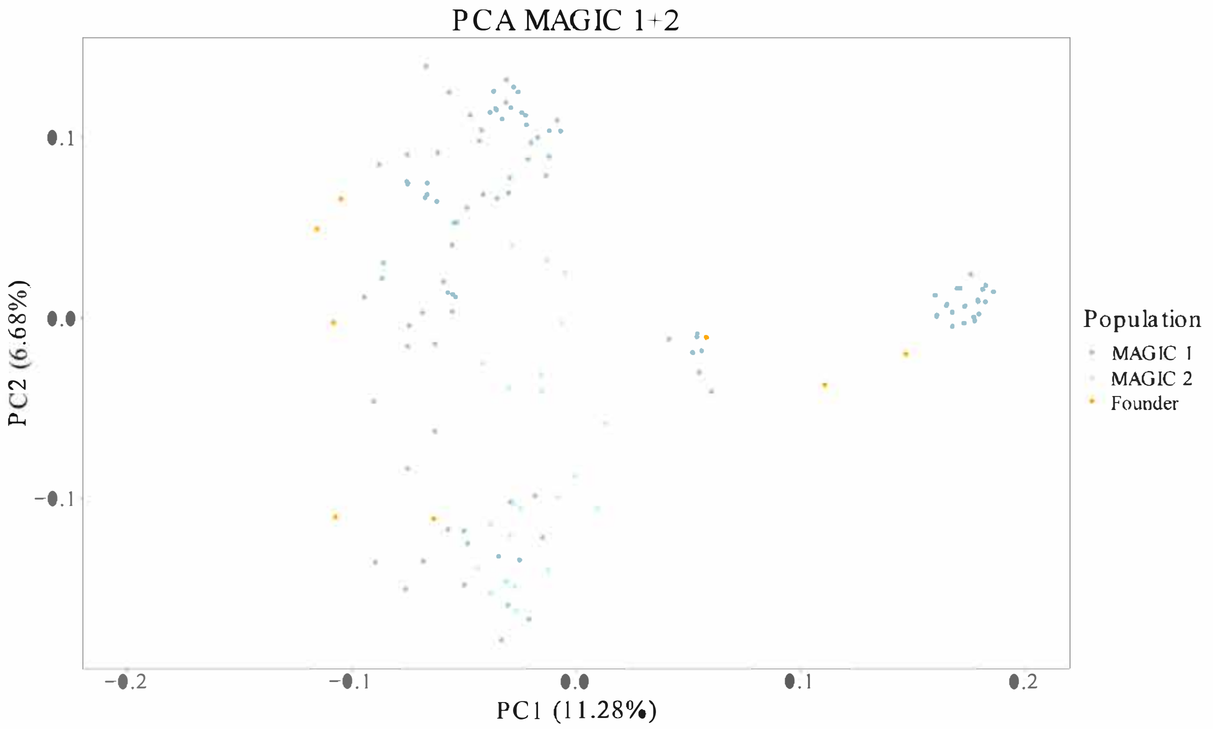
PCA MAGIC 1+2
0.1
0.0
−0.1
PC2 (6.68%)
−0.2
−0.1
0.0
0.1
0.2
PC1 (11.28%)
Population
MAGIC 1
MAGIC 2
Founder

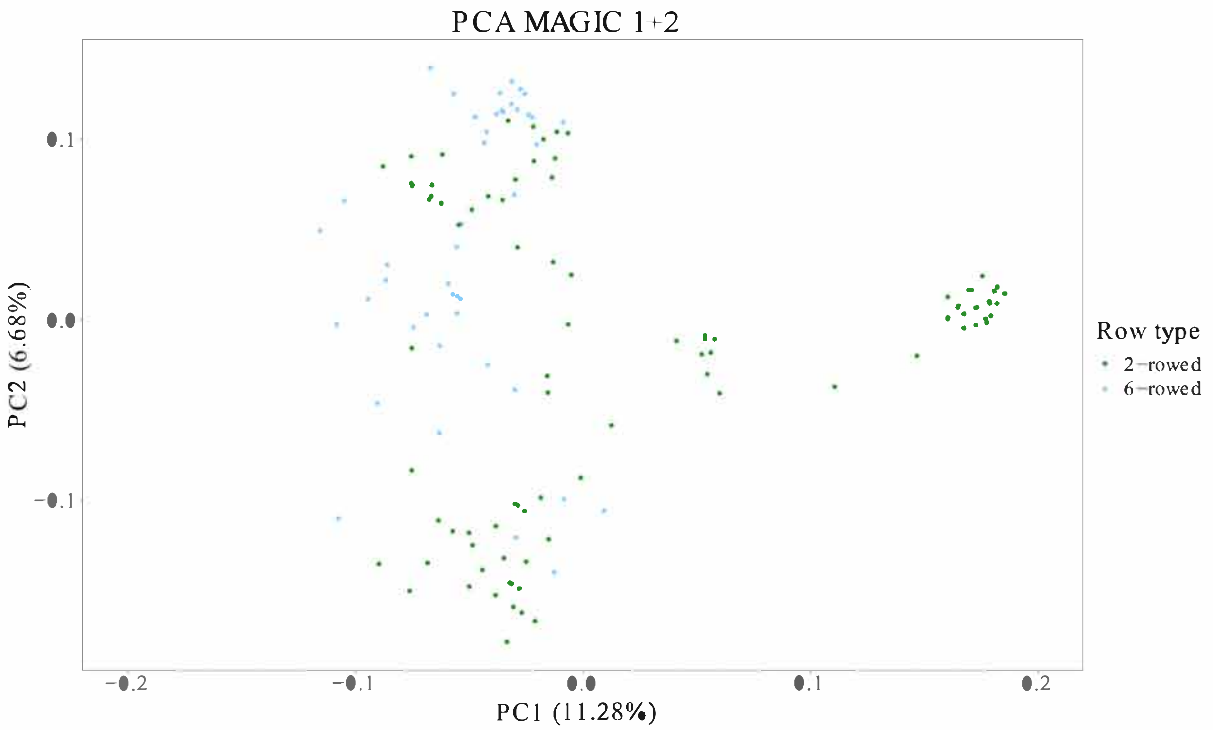
PCA MAGIC 1+2
0.1
0.0
−0.1
PC2 (6.68%)
−0.2
−0.1
0.0
0.1
0.2
PC1 (11.28%)
Row type
2−rowed
6−rowed

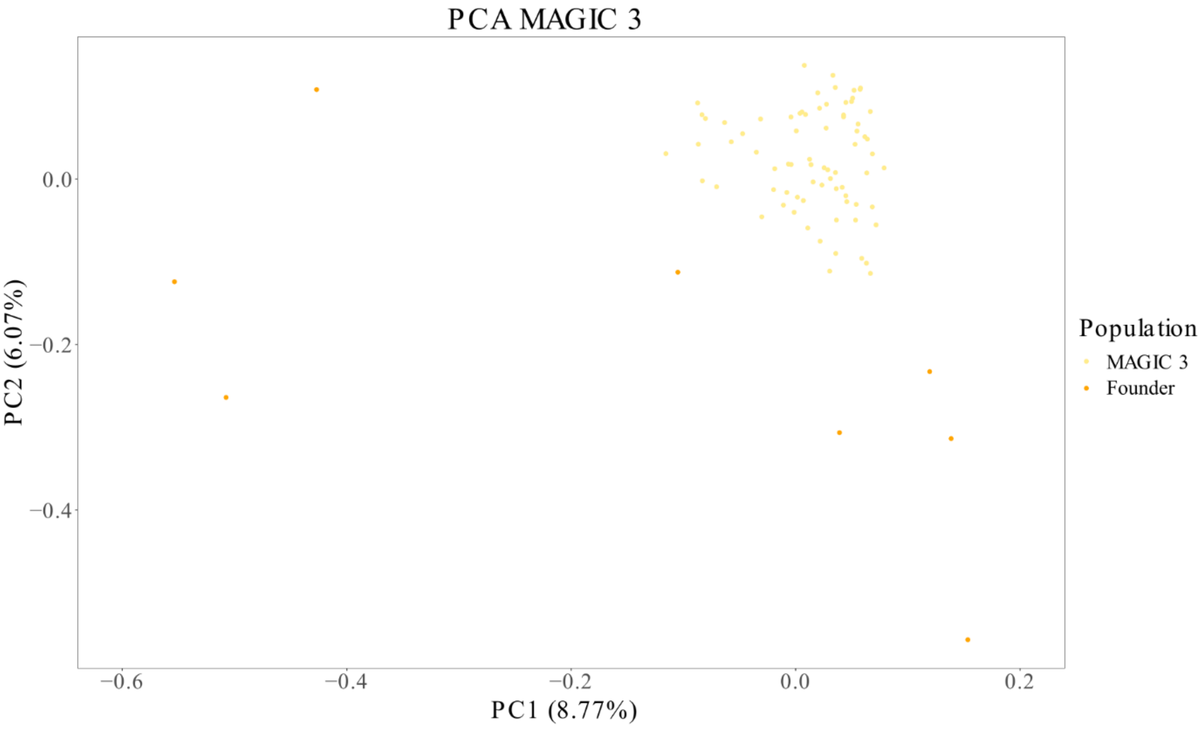
PCA MAGIC 3
0.0
−0.2
−0.4
PC2 (6.07%)
−0.6
−0.4
−0.2
0.0
0.2
PC1 (8.77%)
Population
MAGIC 3
Founder

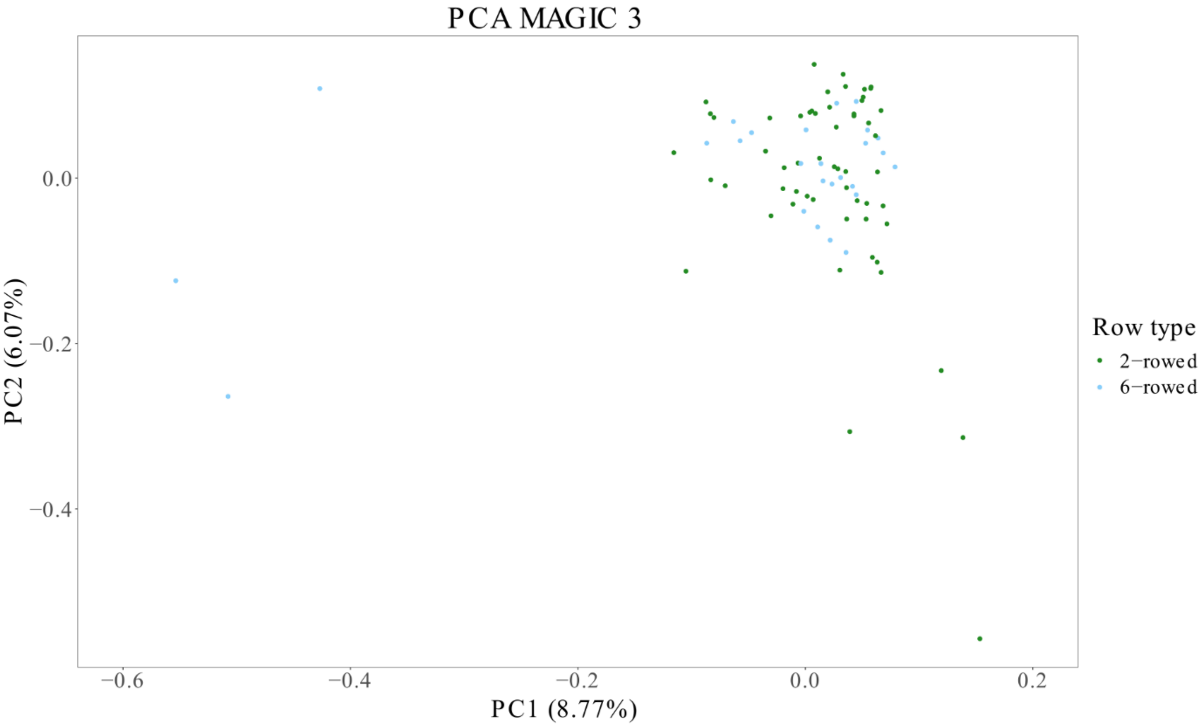
PCA MAGIC 3
0.0
−0.2
−0.4
PC2 (6.07%)
−0.6
−0.4
−0.2
0.0
0.2
PC1 (8.77%)
Row type
2−rowed
6−rowed

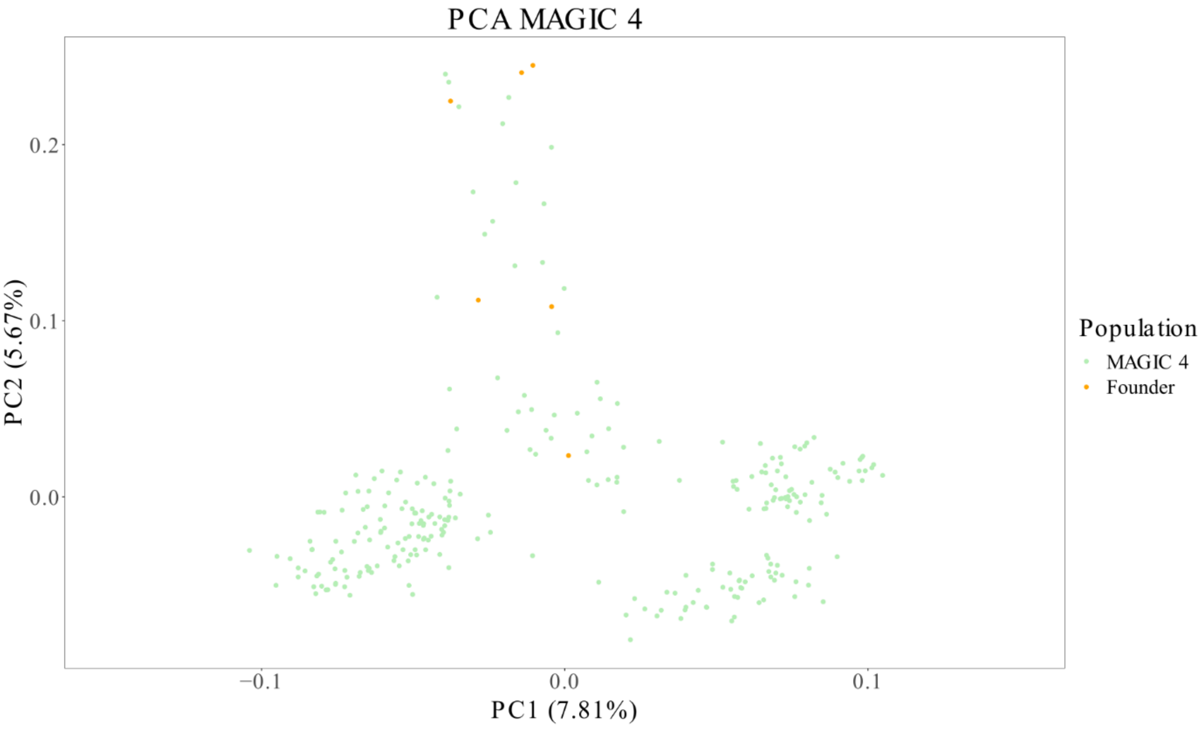
PCA MAGIC 4
PC2 (5.67%)
0.2
0.1
0.0
−0.1
0.0
0.1
PC1 (7.81%)
Population
MAGIC 4
Founder

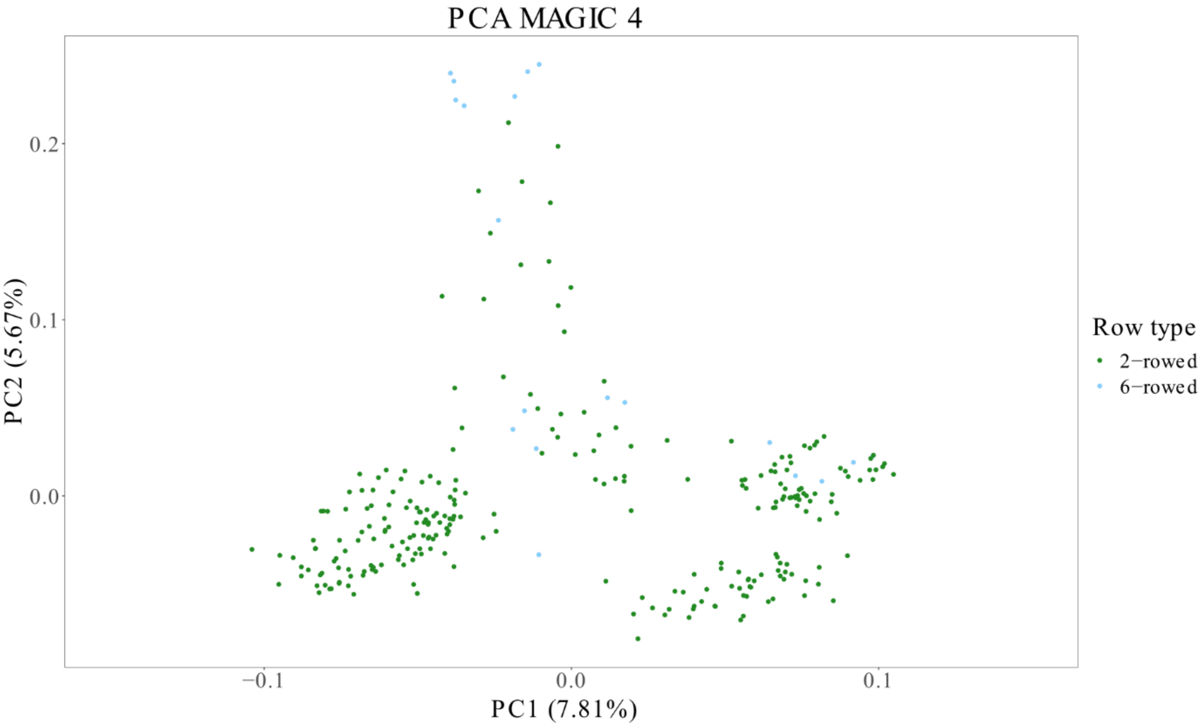
PCA MAGIC 4
PC2 (5.67%)
0.2
0.1
0.0
−0.1
0.0
0.1
PC1 (7.81%)
Row type
2−rowed
6−rowed

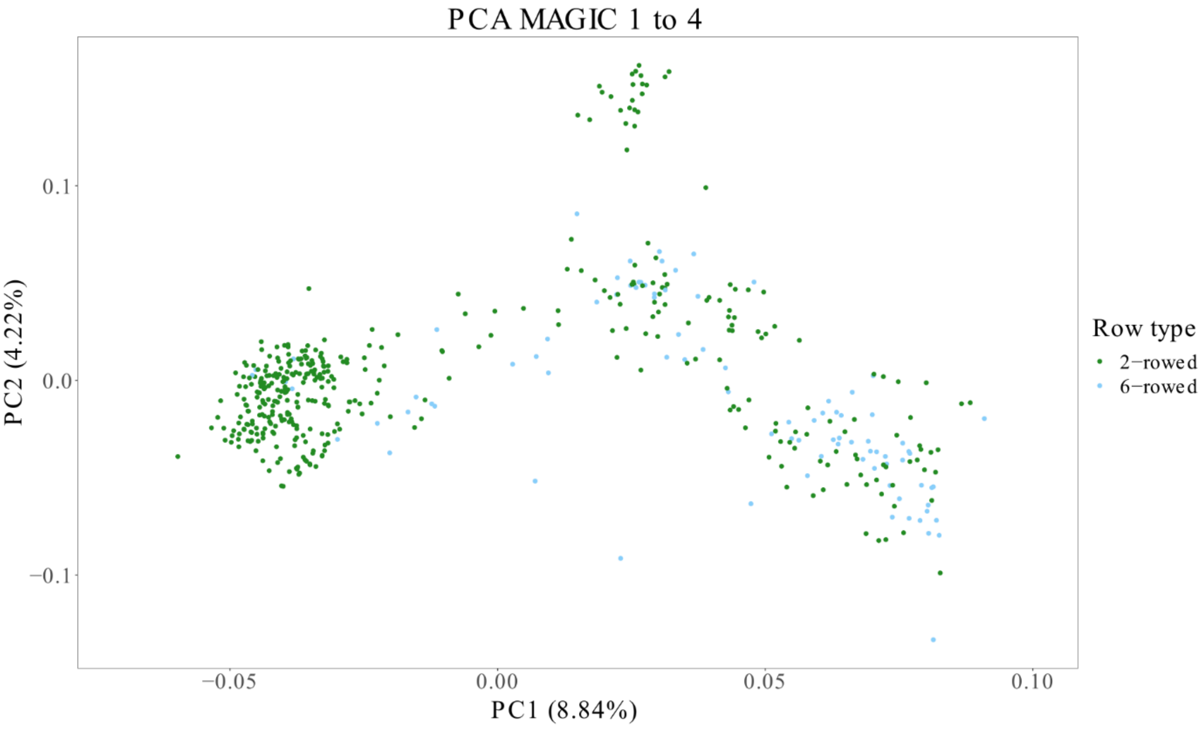
PCA MAGIC 1 to 4
PC2 (4.22%)
PC1 (8.84%)
0.1
0.0
−0.1
−0.05
0.00
0.05
0.10
Row type
2−rowed
6−rowed

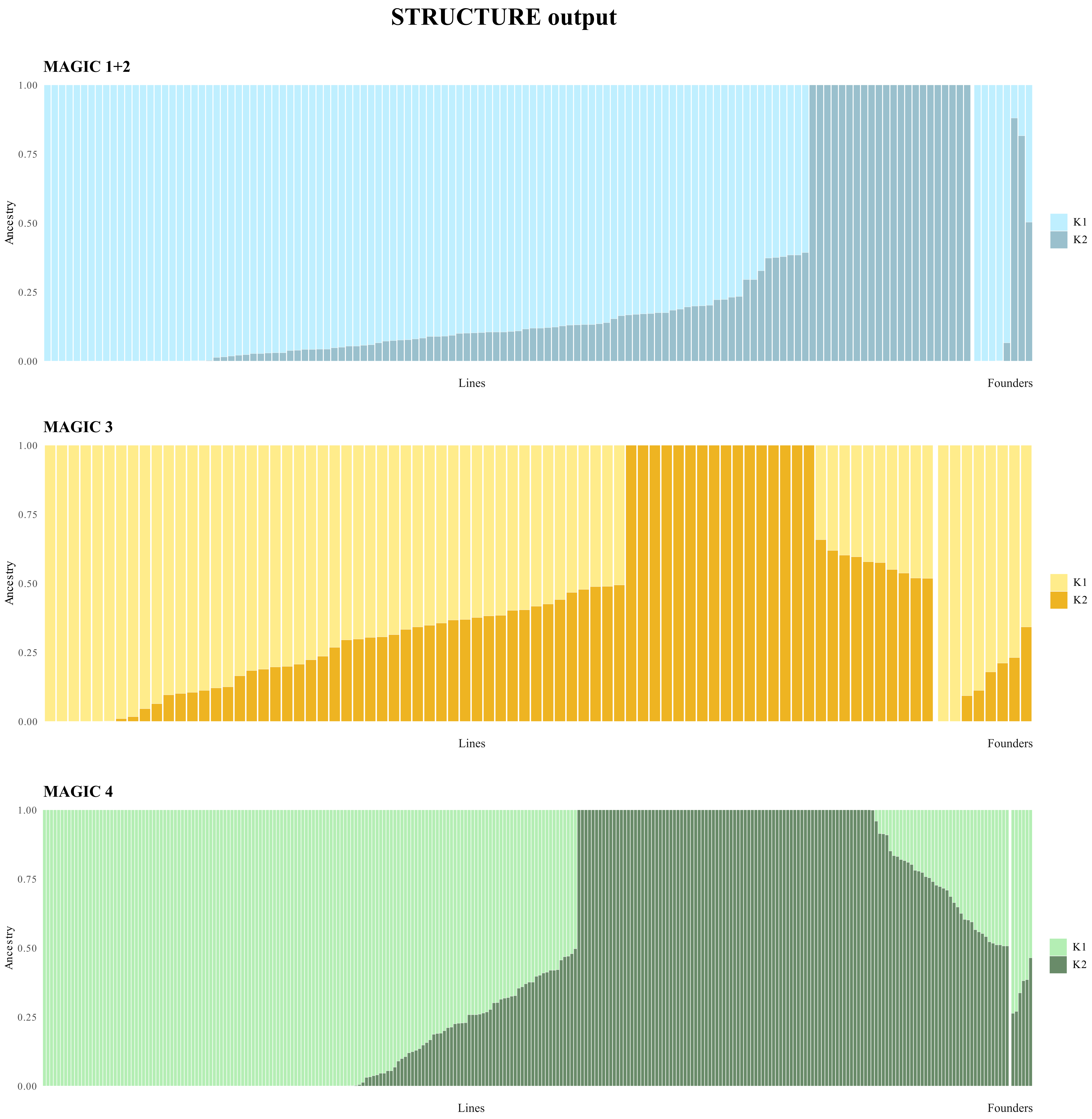
STRUCTURE output
MAGIC 1+2
Ancestry
1.00
0.75
0.50
0.25
0.00
Lines
Founders
K1
K2
MAGIC 3
Ancestry
1.00
0.75
0.50
0.25
0.00
Lines
Founders
K1
K2
MAGIC 4
Ancestry
1.00
0.75
0.50
0.25
0.00
Lines
Founders
K1
K2
