# Supplementary material for: You Had Me at “MAGIC”!: Four Barley MAGIC Populations Reveal Novel Resistance QTL for Powdery Mildew
Source: Genes (Basel). 2020 Dec 18;11(12):1512. doi: 10.3390/genes11121512 (PMC7766815; doi:10.3390/genes11121512)
Supplement: Supplementary file 1 [file genes-11-01512-s001.zip › Figure S3.pdf]

Figure S3

You had me at "MAGIC"! Four barley MAGIC populations reveal novel resistance QTL for Powdery Mildew

Fluturë Novakazi [1], Lene Krusell [2], Jens Due Jensen [3], Jihad Orabi [3], The PPP Barley Consortium, Ahmed Jahoor [3], Therése Bengtsson [1*]

[1] Department of Plant Breeding, Swedish University of Agricultural Sciences, Box 101, 230 53, Alnarp Sweden

[2] Sejet Plant Breeding, Nørremarksvej 67, 8700 Horsens, Denmark

[3] Nordic Seed A/S, Kornmarken 1, 8464 Galten Denmark

*Correspondence: Therése Bengtsson: therese.bengtsson@slu.se

Content

LD decay plots for each individual MAGIC population per chromosome and across chromosomes and for the combined panel (MAGIC 1 to 4) for each chromosome

The PPP Barley Consortium
Inger Åhman (Department of Plant Breeding, Swedish University of Agricultural Sciences, Box 101, 230 53 Alnarp, Sweden),
Therése Bengtsson (Department of Plant Breeding, Swedish University of Agricultural Sci-ences, Box 101, 230 53 Alnarp, Sweden),
Fluturë Novakazi (Department of Plant Breeding, Swedish University of Agricultural Scienc-es, Box 101, 230 53 Alnarp, Sweden),
Outi Manninen (Boreal Plant Breeding Ltd, Myllytie 10, 31600 Jokioinen, Finland),
Merja Veteläinen (Boreal Plant Breeding Ltd, Myllytie 10, 31600 Jokioinen, Finland),
Mika Isolahti, (Boreal Plant Breeding Ltd, Myllytie 10, 31600 Jokioinen, Finland),
Muath Alsheikh (Graminor A/S, Hommelstadvegen 60, 2322 Ridabu, Norway),
Stein Bergersen (Graminor A/S, Hommelstadvegen 60, 2322 Ridabu, Norway),
Constantin Jansen (Graminor A/S, Hommelstadvegen 60, 2322 Ridabu, Norway),
Susanne Windju (Graminor A/S, Hommelstadvegen 60, 2322 Ridabu, Norway),
Marja Jalli, (Natural Resources Institute Finland (Luke), Tietotie 4, 31600 Jokioinen, Finland),
Juho Hautsalo, (Natural Resources Institute Finland (Luke), Survontie 9, 40500 Jyväskylä, Finland,
Ahmed Jahoor (NordicSeed A/S, Kornmarken 1, 8464 Galten, Denmark) (project leader),
Jens Due Jensen (Nordic Seed A/S, Kornmarken 1, 8464Galten, Denmark),
Jihad Orabi (Nordic Seed A/S, Kornmarken 1, 8464 Galten, Denmark),
Nana Vagndorf Nordestgaard (Nordic Seed A/S, Kornmarken 1, 8464 Galten, Denmark),
Lene Krusell (Sejet Plant Breeding, Nørremarksvej 67, 8700 Horsens, Denmark),
Rasmus Lund Hjortshøj (Sejet Plant Breeding, Nørremarksvej 67, 8700 Horsens, Denmark),
Charlotte Damgård Robertsen, (Sejet Plant Breeding, Nørremarksvej 67, 8700 Horsens, Den-mark),
Magnus Göransson (The Agricultural University of Iceland, Faculty of Land and Animal Re-sources, Hvanneyri, 311 Borgarnes, Iceland)

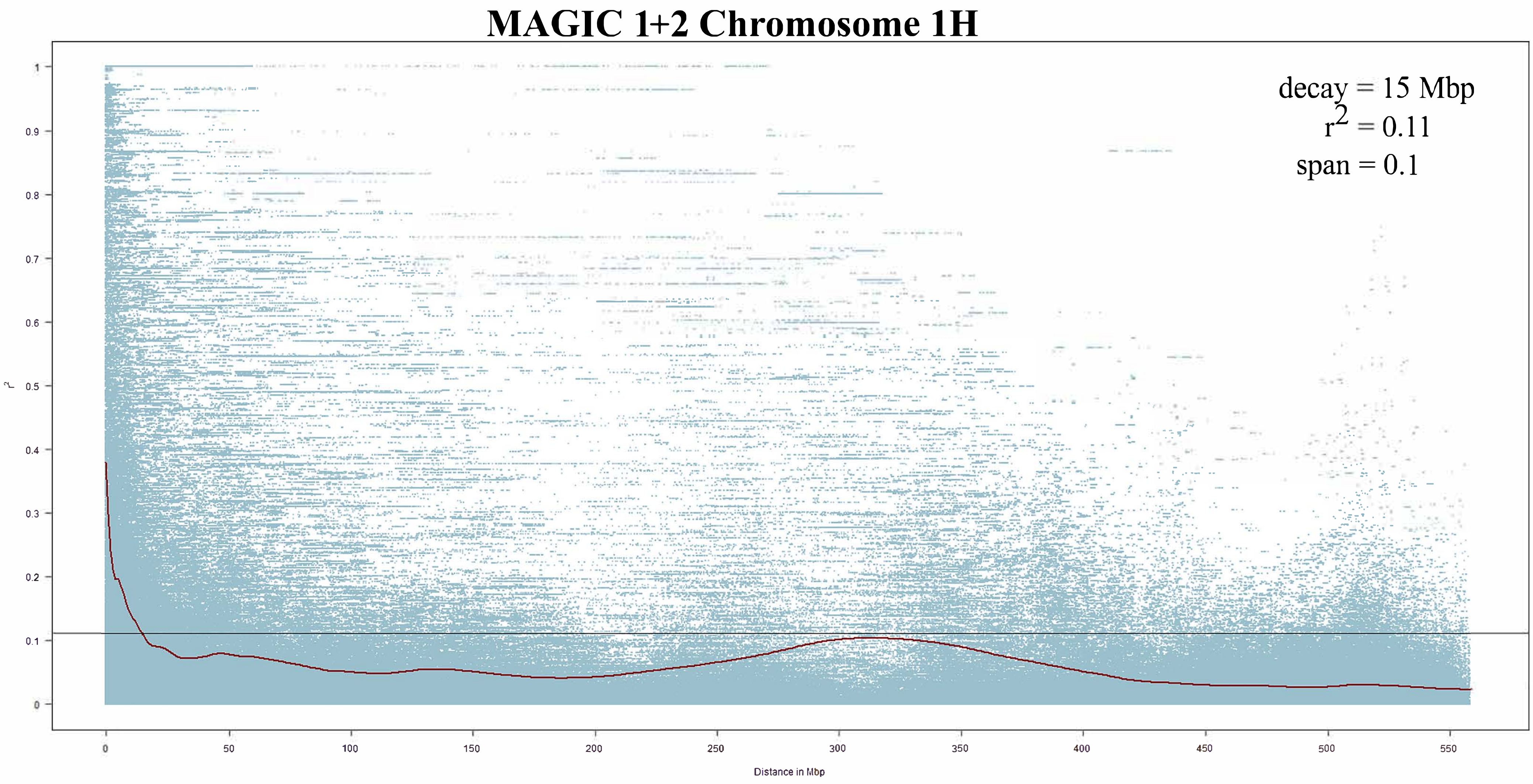
MAGIC 1+2 Chromosome 1H
decay = 15 Mbp
r^2 = 0.11
span = 0.1
r^2
0
0.1
0.2
0.3
0.4
0.5
0.6
0.7
0.8
0.9
1
0
50
100
150
200
250
300
350
400
450
500
550
Distance in Mbp

MAGIC 1+2 Chromosome 2H

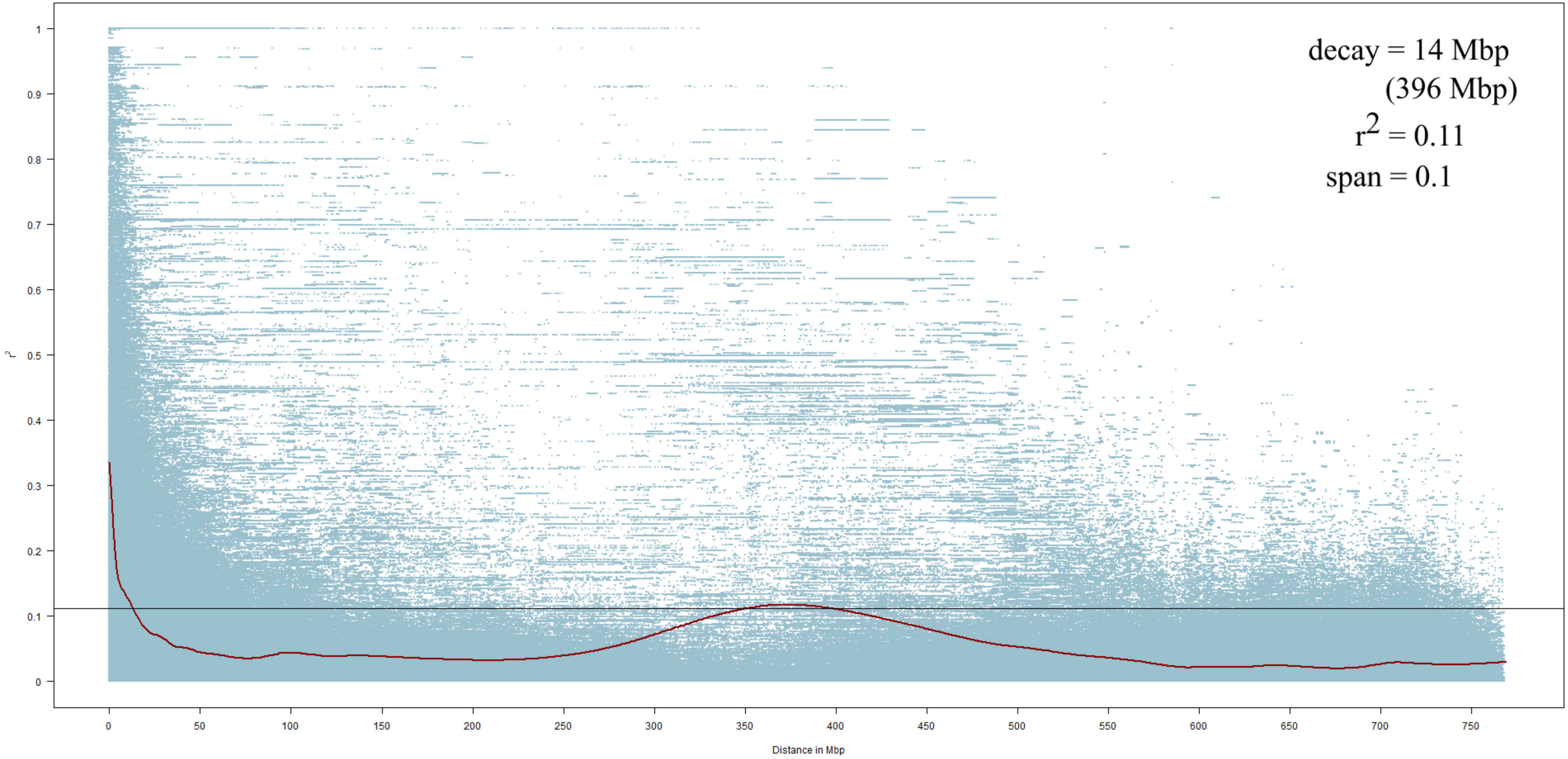

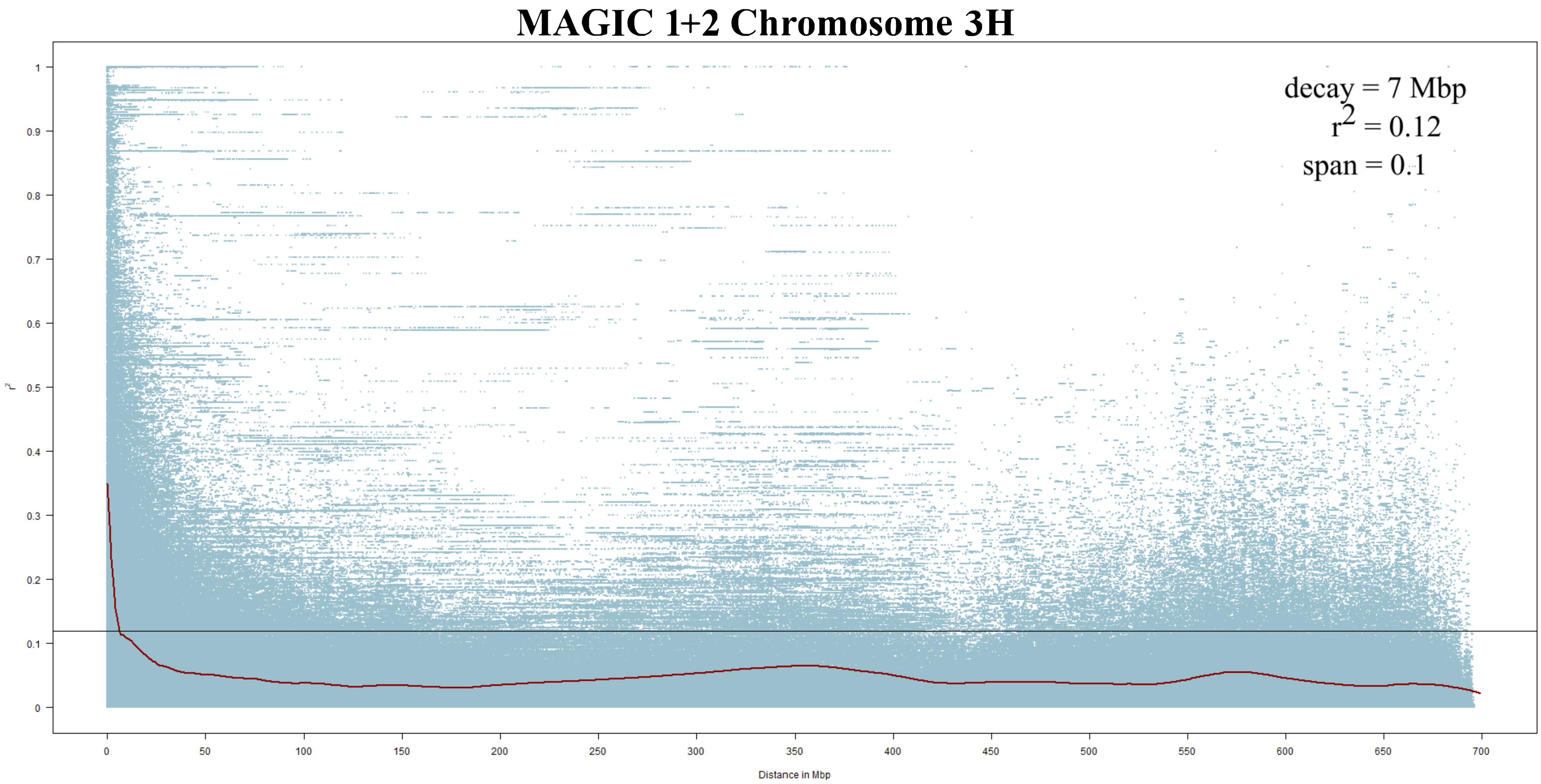
MAGIC 1+2 Chromosome 3H
decay = 7 Mbp
r2 = 0.12
span = 0.1
r2
Distance in Mbp
0
0.1
0.2
0.3
0.4
0.5
0.6
0.7
0.8
0.9
1
0
50
100
150
200
250
300
350
400
450
500
550
600
650
700

MAGIC 1+2 Chromosome 4H

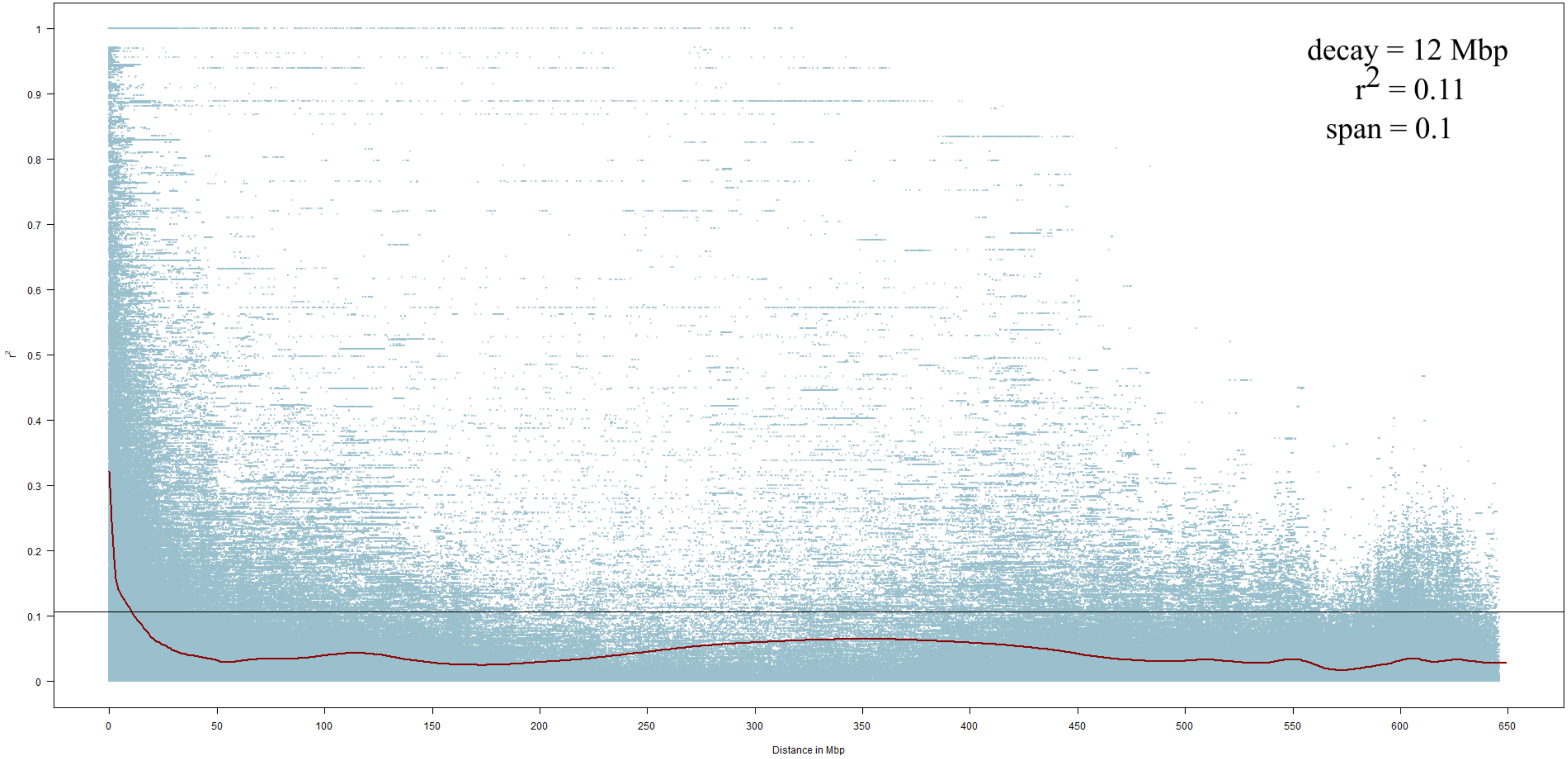

MAGIC 1+2 Chromosome 5H

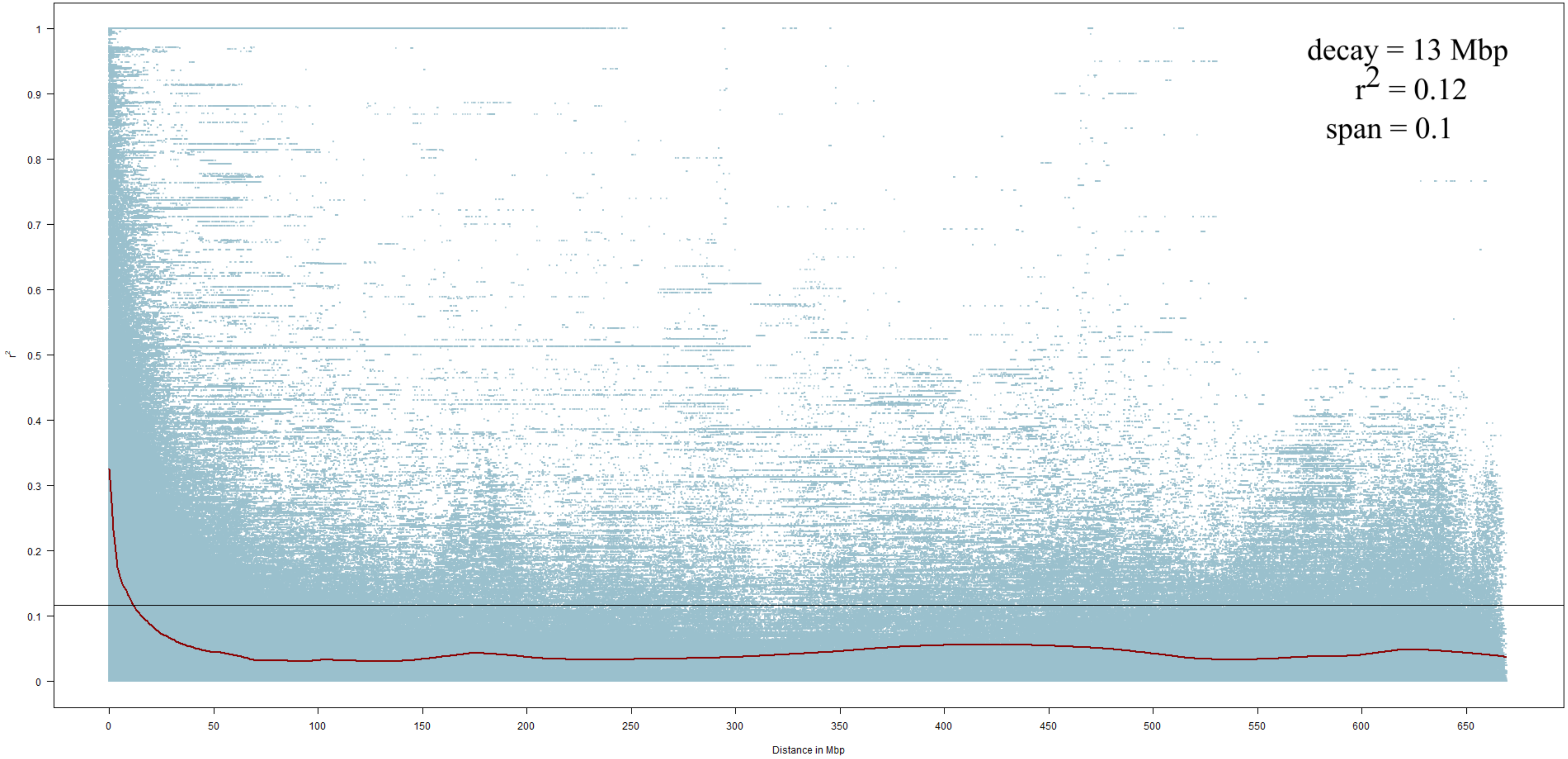

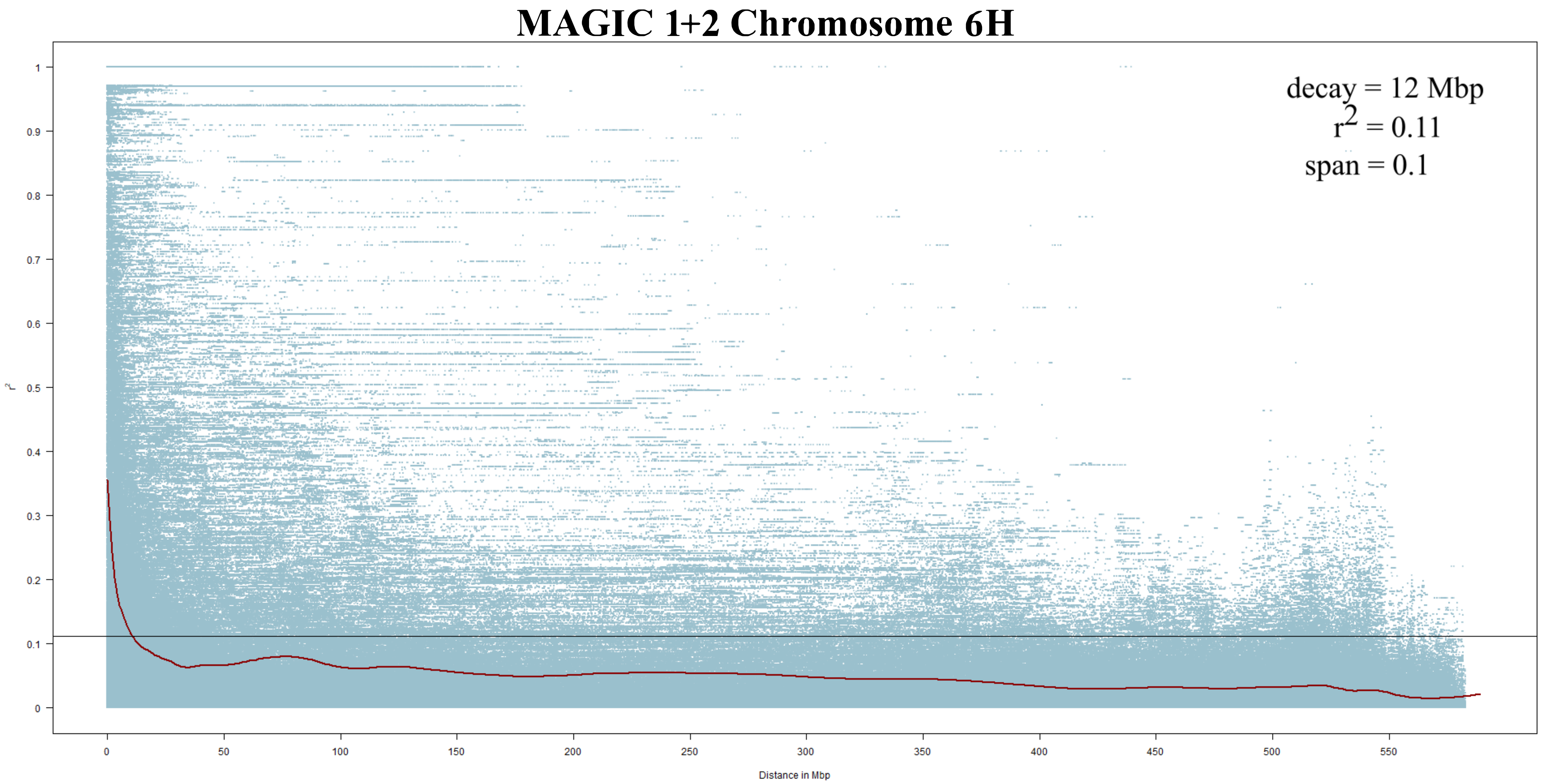
MAGIC 1+2 Chromosome 6H
decay = 12 Mbp
r2 = 0.11
span = 0.1
r2
1
0.9
0.8
0.7
0.6
0.5
0.4
0.3
0.2
0.1
0
0
50
100
150
200
250
300
350
400
450
500
550
Distance in Mbp

MAGIC 1+2 Chromosome 7H

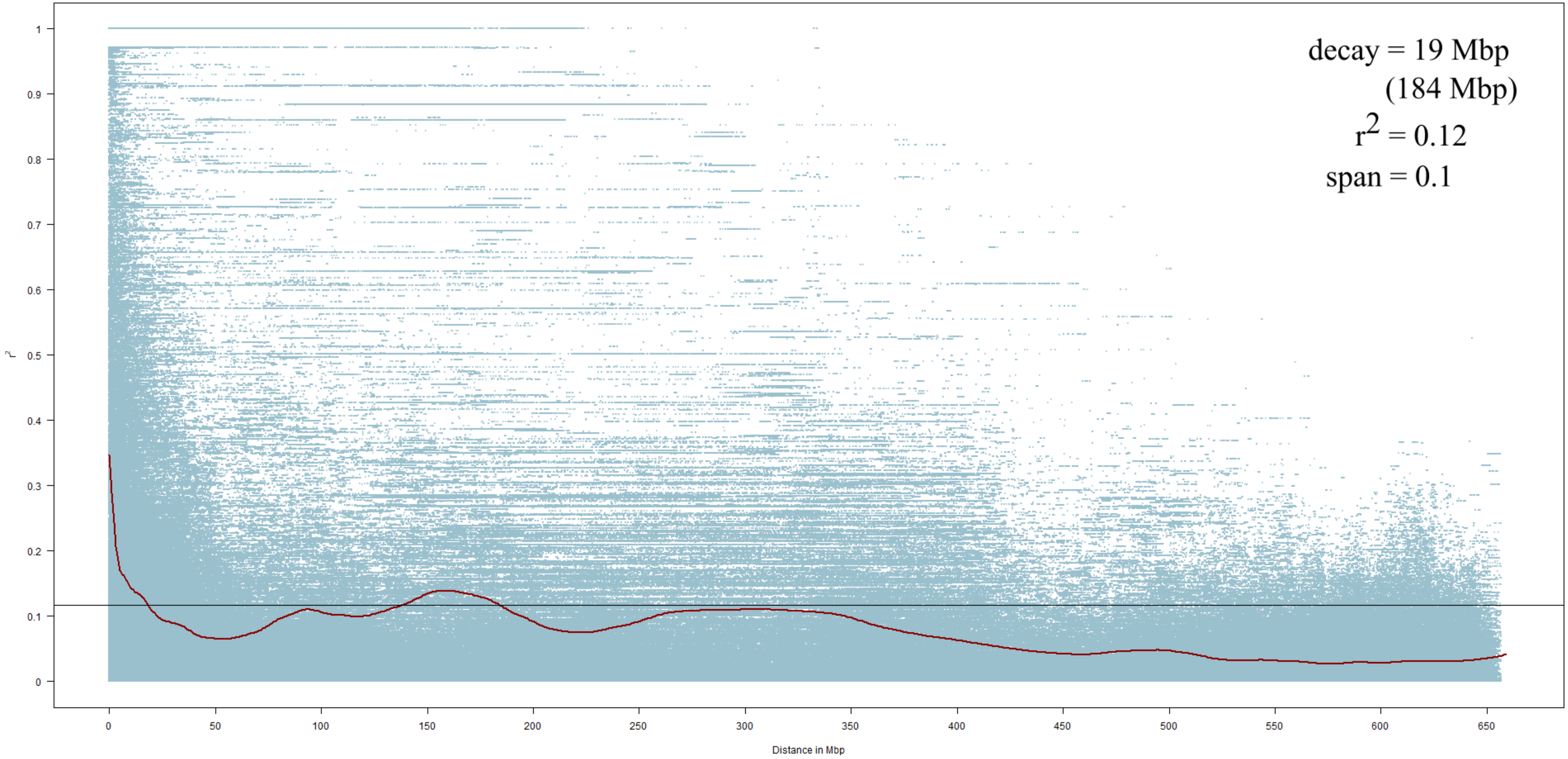

MAGIC 1+2 genome-wide LD decay

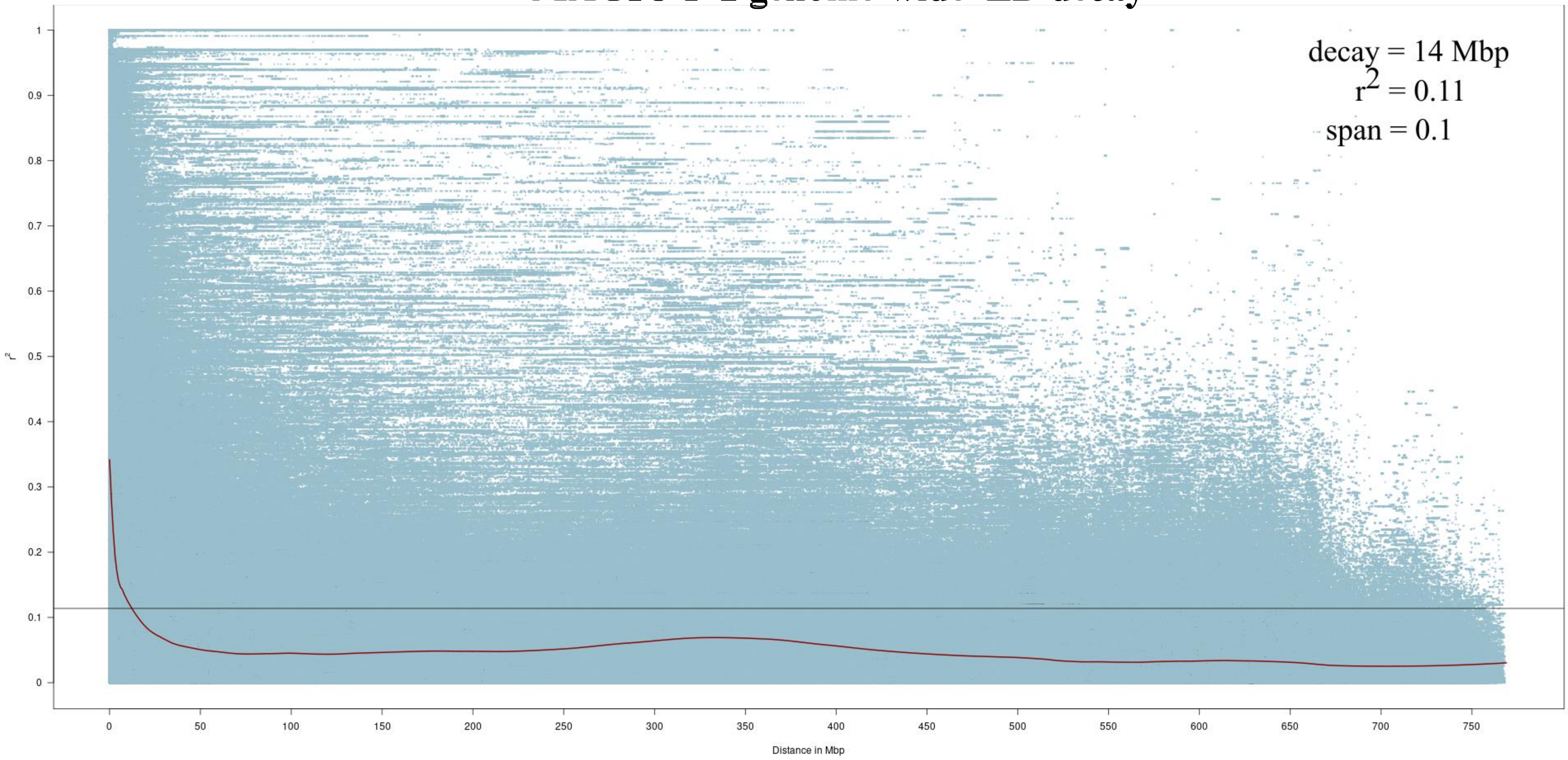

MAGIC 3 Chromosome 1H

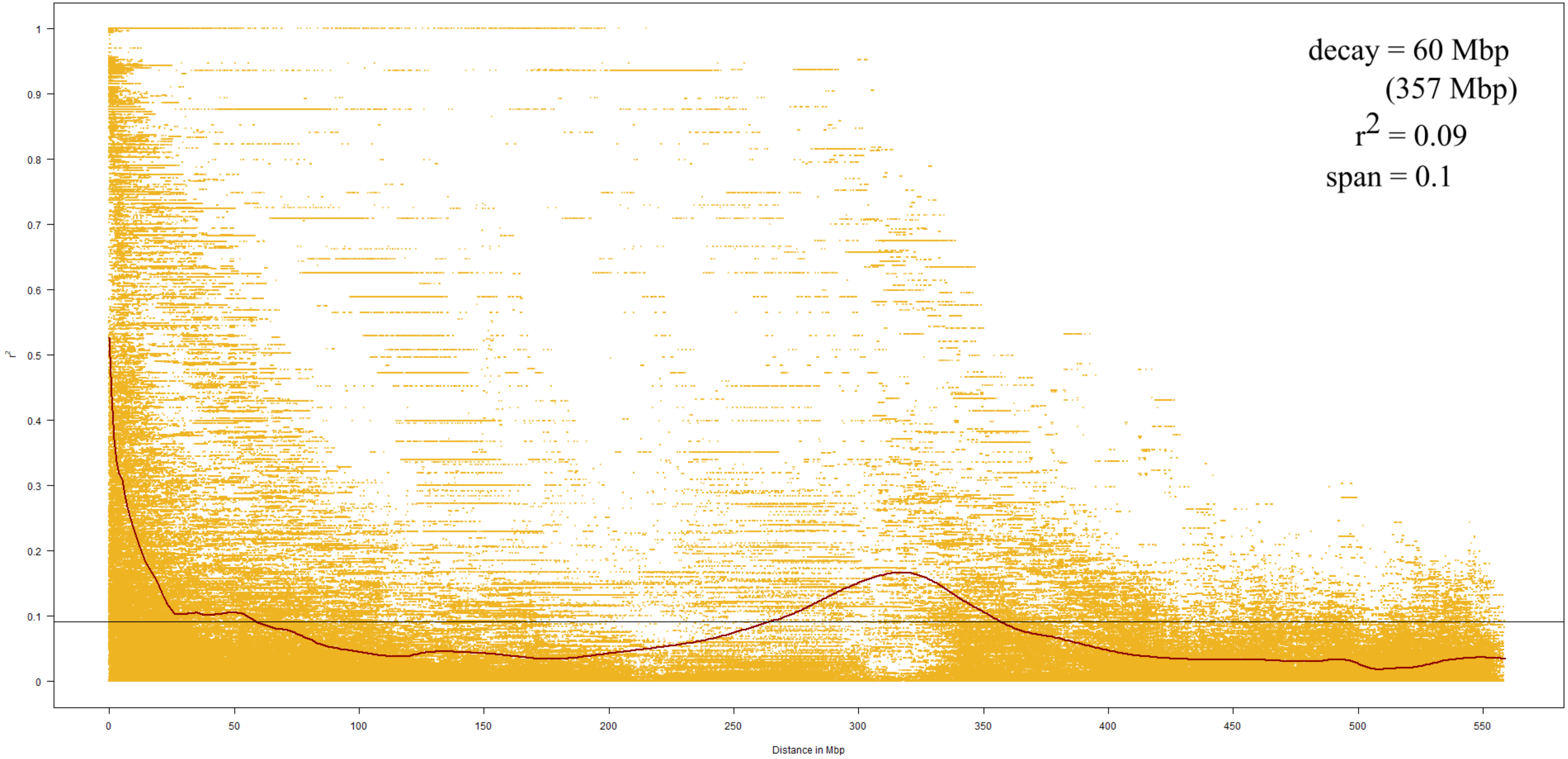

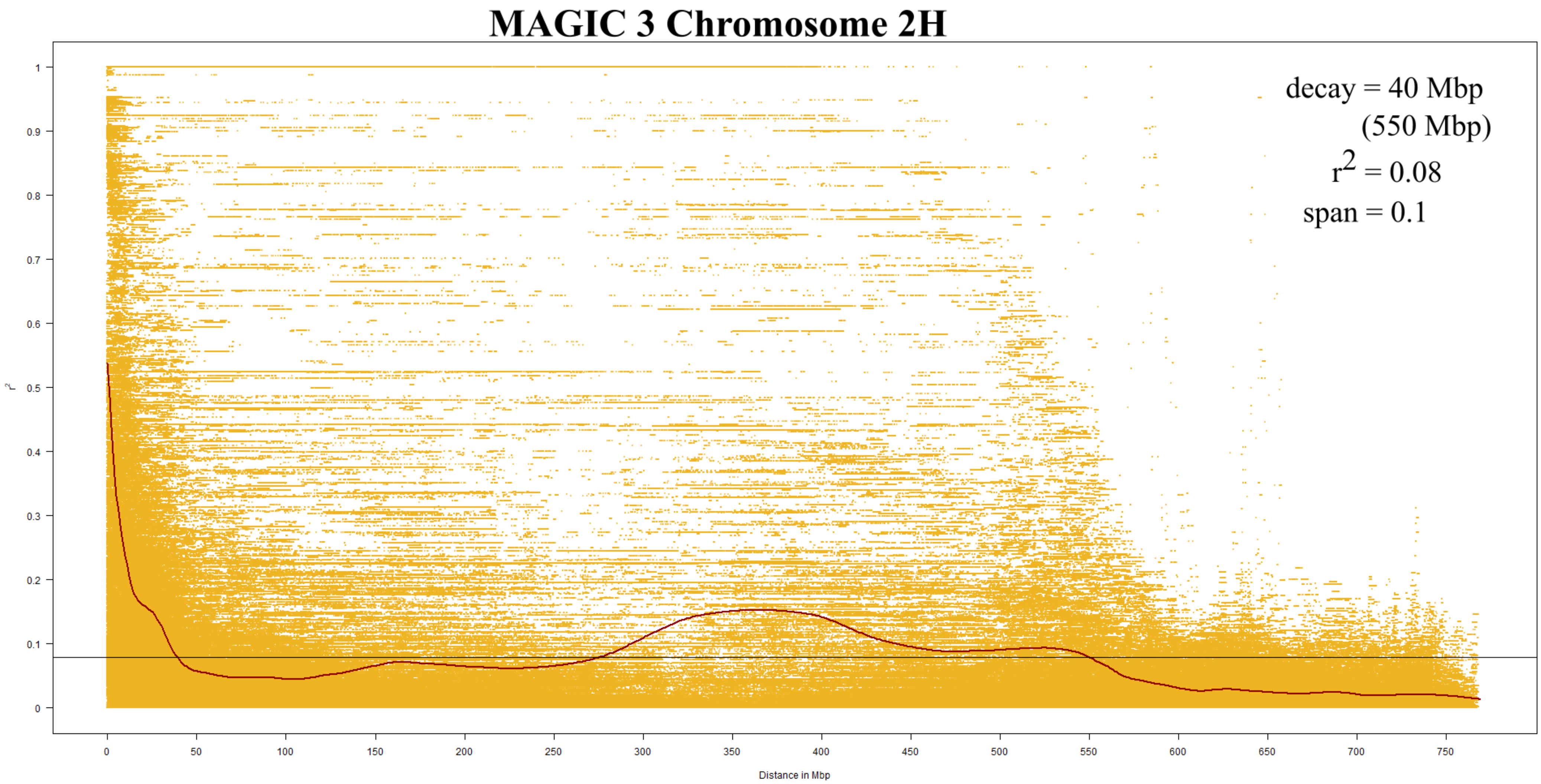
MAGIC 3 Chromosome 2H
decay = 40 Mbp
(550 Mbp)
r2 = 0.08
span = 0.1
r2
Distance in Mbp
0
0.1
0.2
0.3
0.4
0.5
0.6
0.7
0.8
0.9
1
0
50
100
150
200
250
300
350
400
450
500
550
600
650
700
750

MAGIC 3 Chromosome 3H

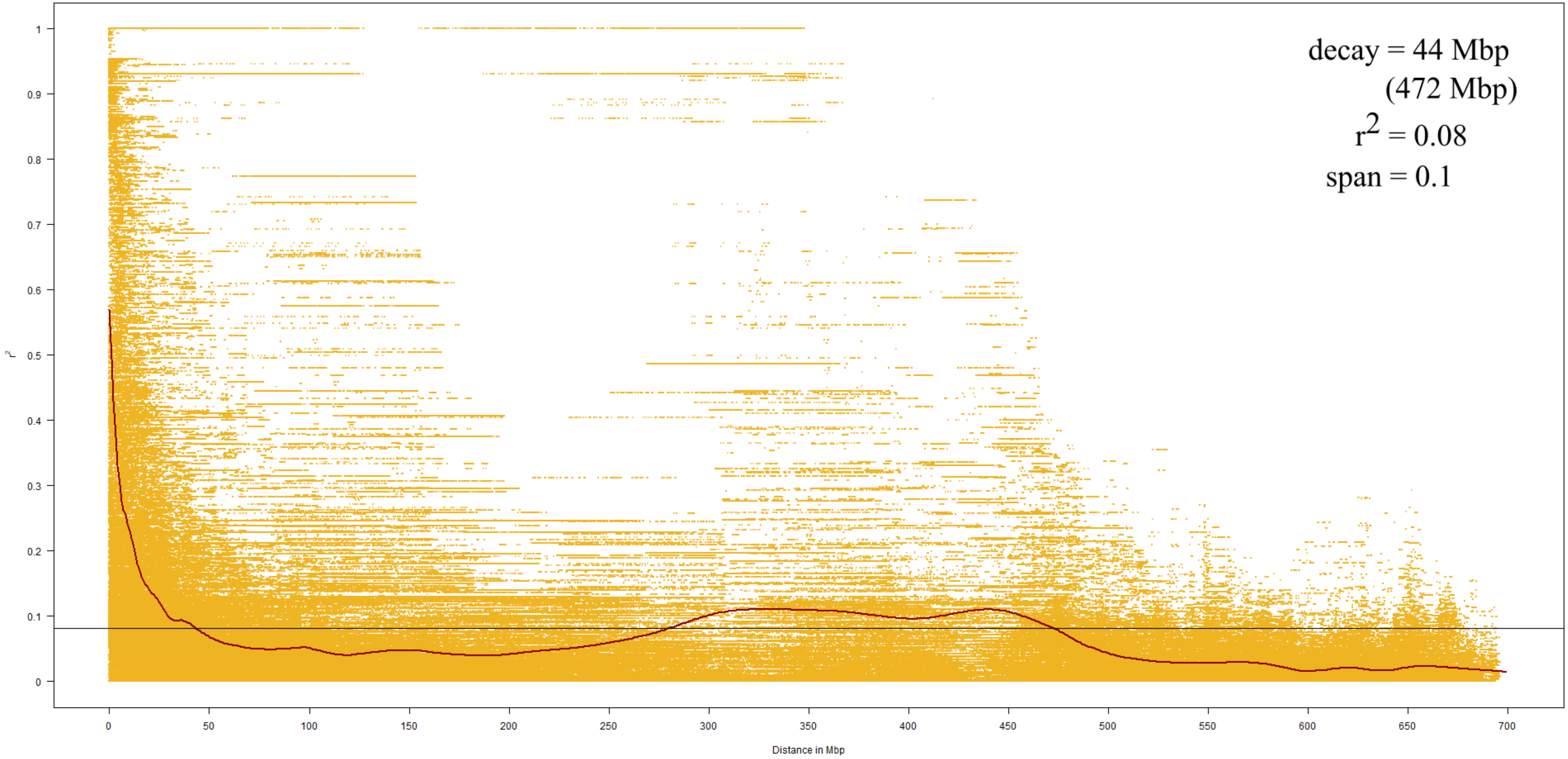

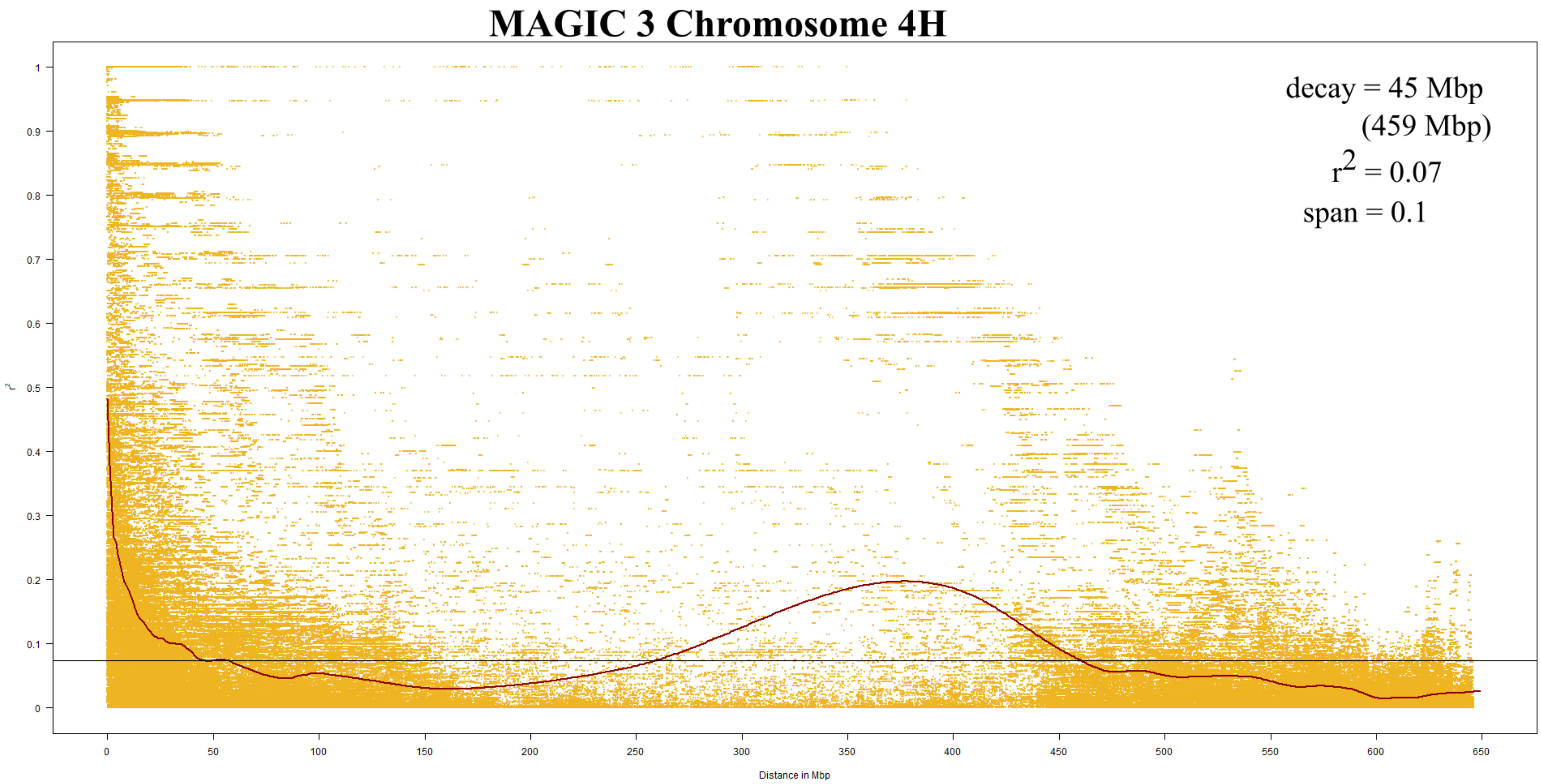
MAGIC 3 Chromosome 4H
decay = 45 Mbp
(459 Mbp)
r^2 = 0.07
span = 0.1
r^2
Distance in Mbp
0
0.1
0.2
0.3
0.4
0.5
0.6
0.7
0.8
0.9
1
0
50
100
150
200
250
300
350
400
450
500
550
600
650

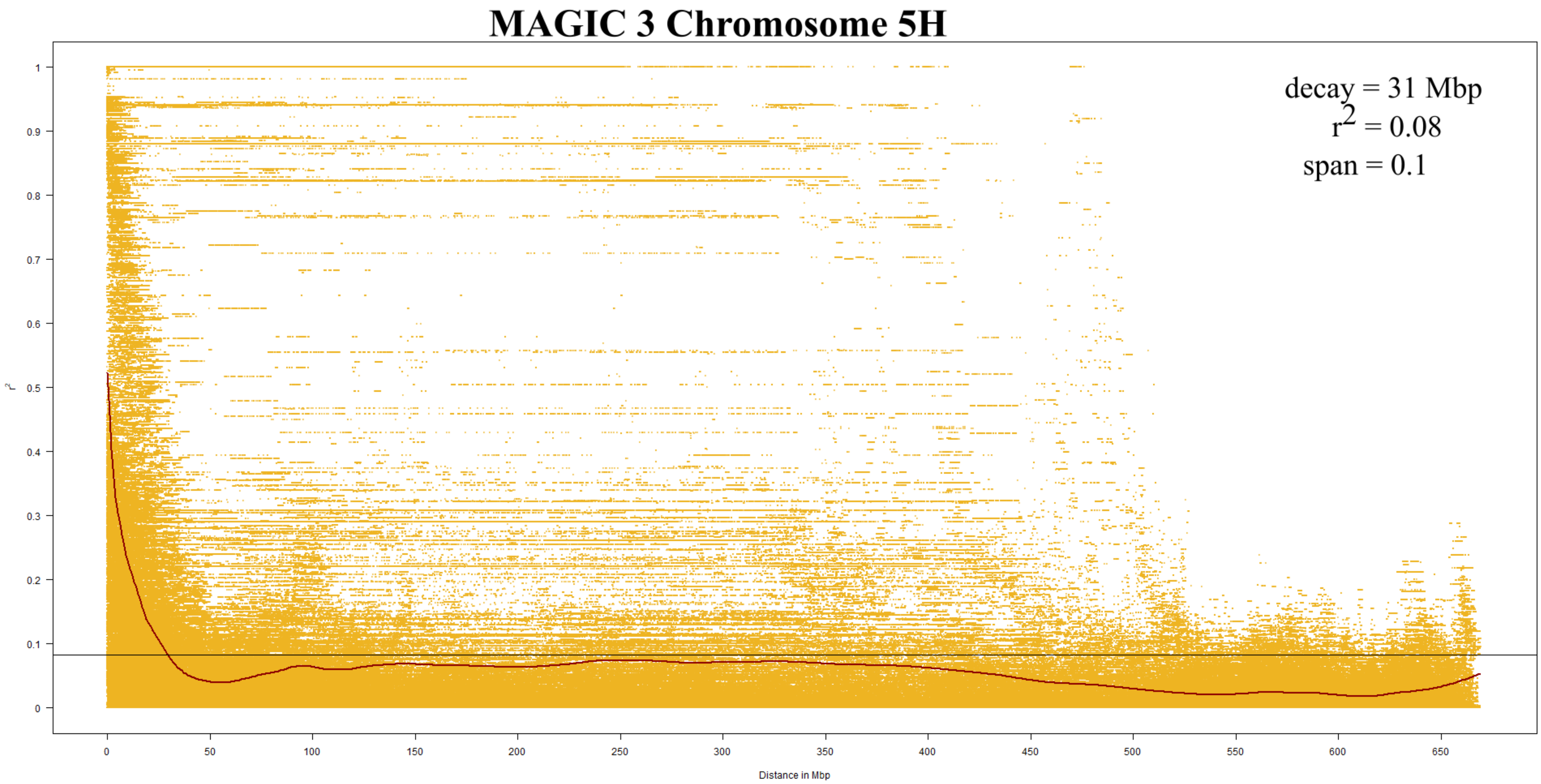
MAGIC 3 Chromosome 5H
decay = 31 Mbp
r2 = 0.08
span = 0.1
r2
0
0.1
0.2
0.3
0.4
0.5
0.6
0.7
0.8
0.9
1
0
50
100
150
200
250
300
350
400
450
500
550
600
650
Distance in Mbp

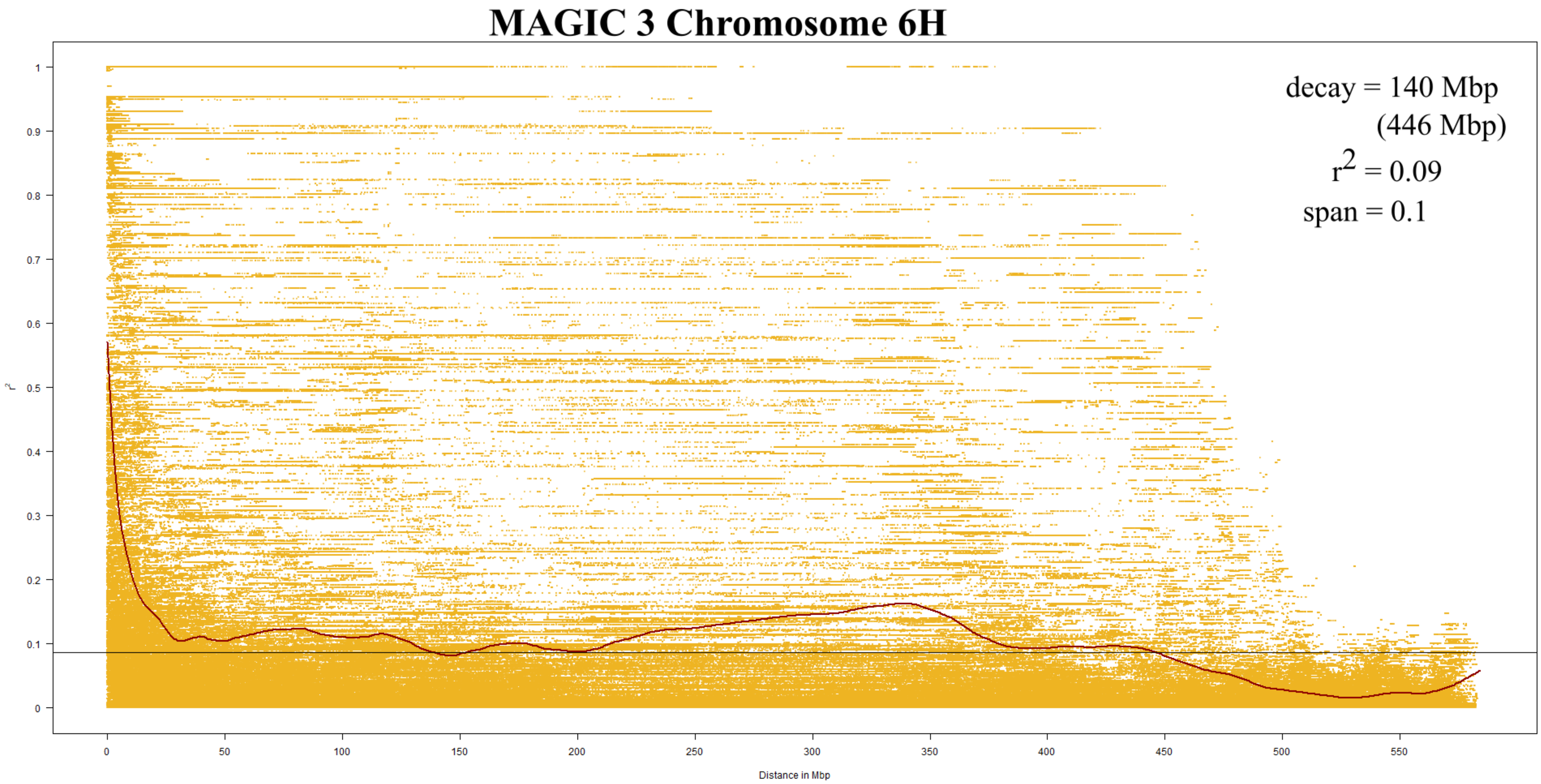
MAGIC 3 Chromosome 6H
decay = 140 Mbp
(446 Mbp)
r2 = 0.09
span = 0.1
r2
0
0.1
0.2
0.3
0.4
0.5
0.6
0.7
0.8
0.9
1
0
50
100
150
200
250
300
350
400
450
500
550
Distance in Mbp

MAGIC 3 Chromosome 7H

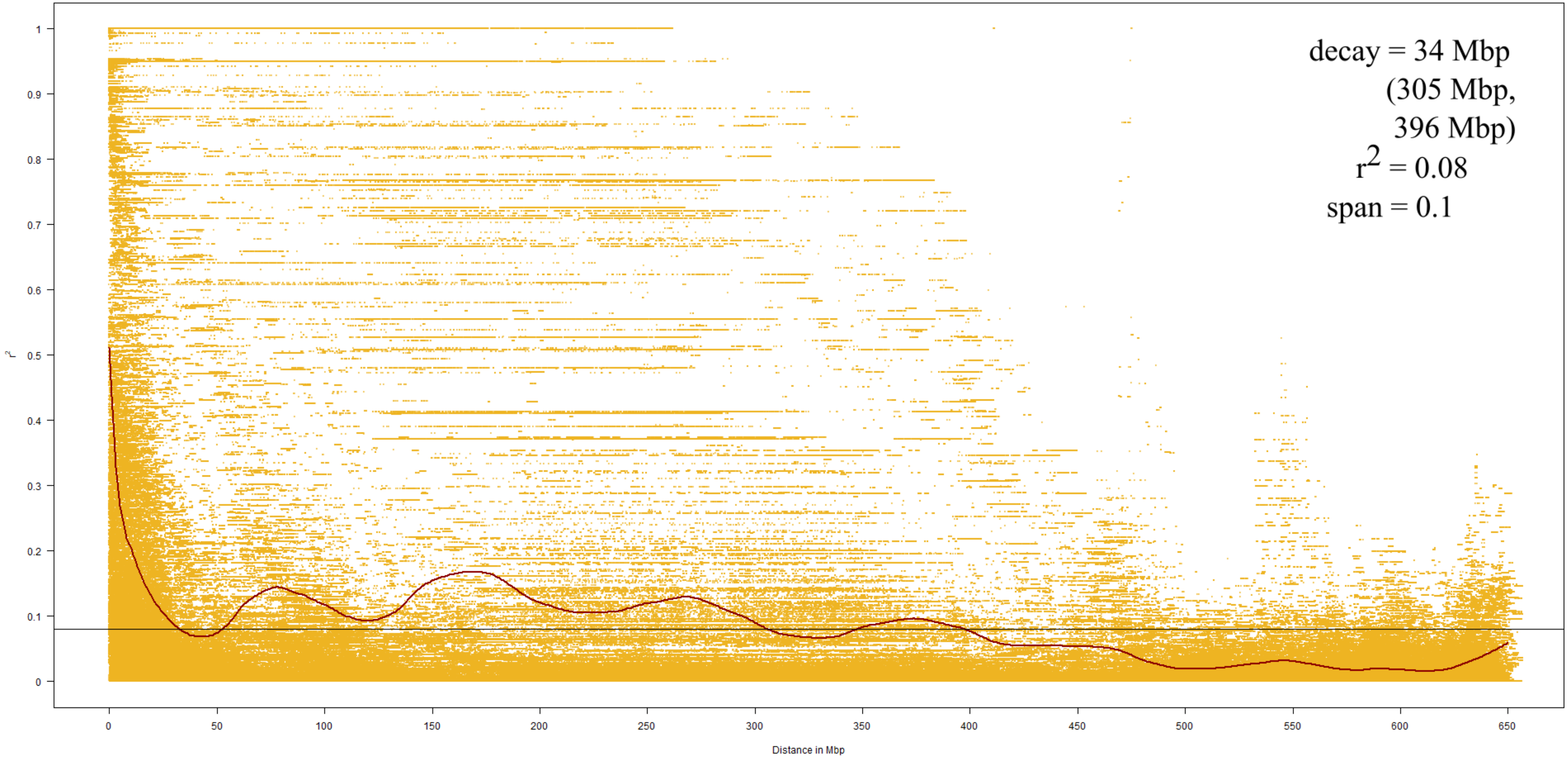

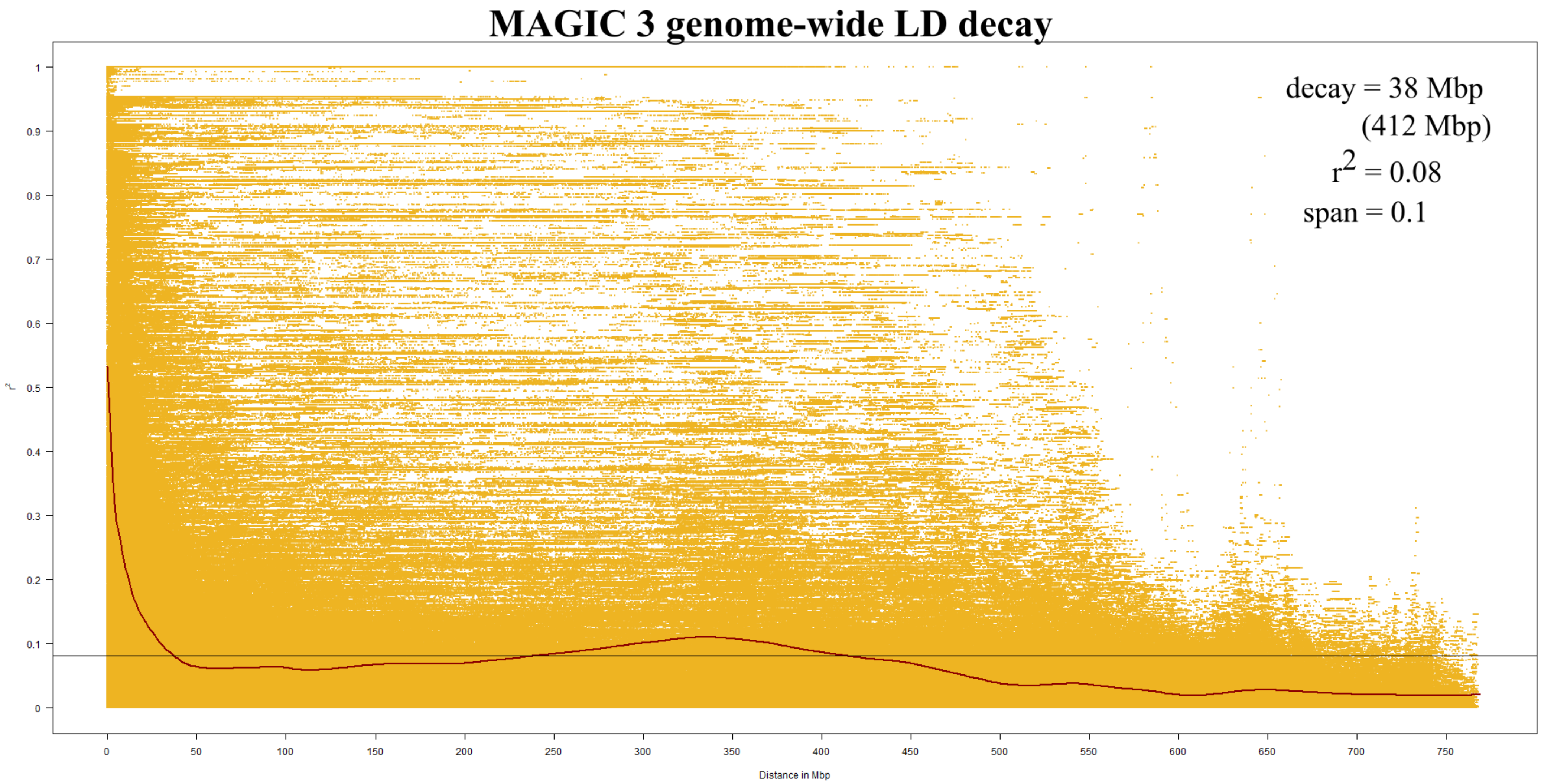

MAGIC 3 genome-wide LD decay
decay = 38 Mbp
(412 Mbp)
r2 = 0.08
span = 0.1
r2
Distance in Mbp
0
0.1
0.2
0.3
0.4
0.5
0.6
0.7
0.8
0.9
1
0
50
100
150
200
250
300
350
400
450
500
550
600
650
700
750

MAGIC 4 Chromosome 1H

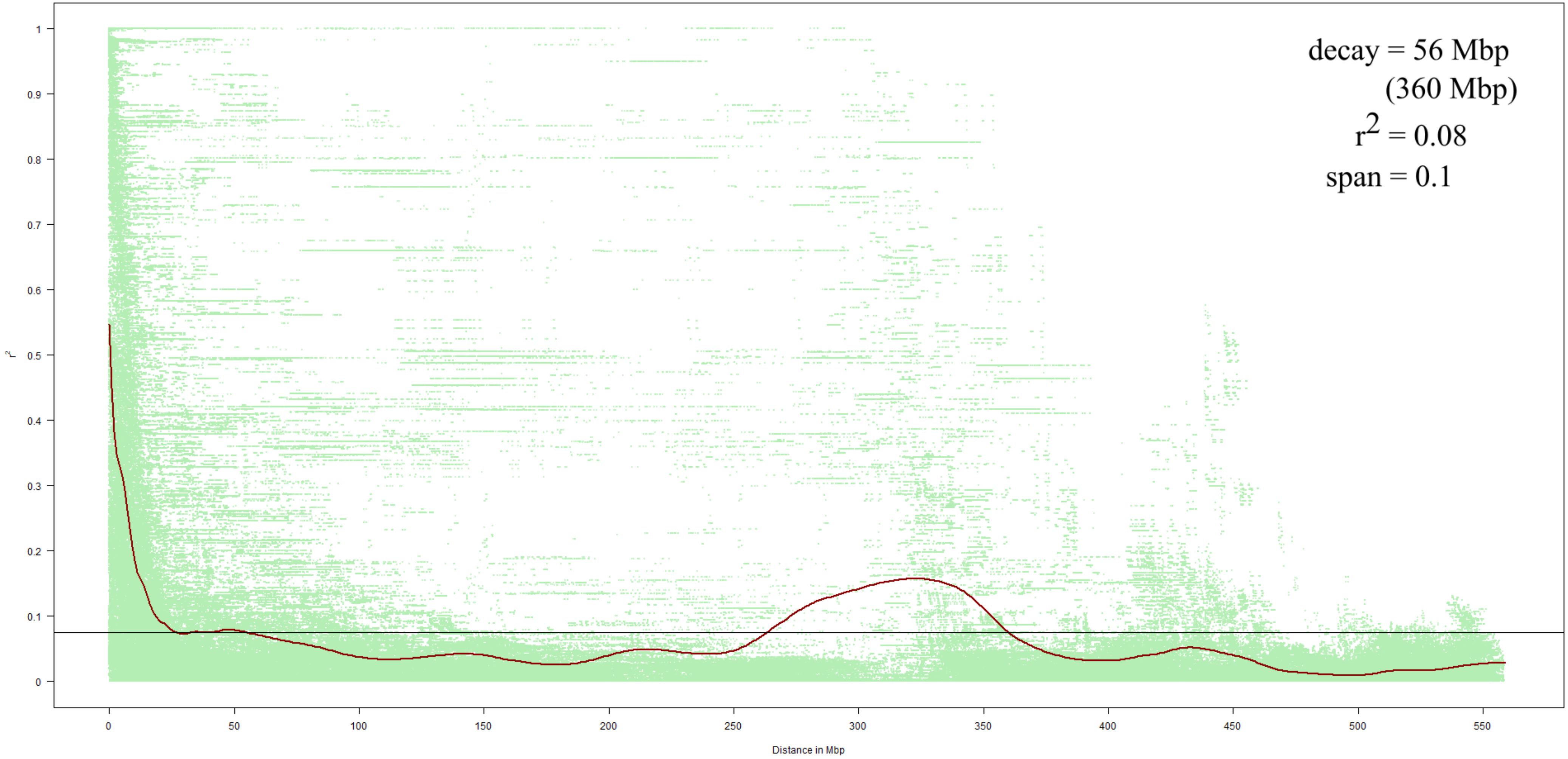

MAGIC 4 Chromosome 2H

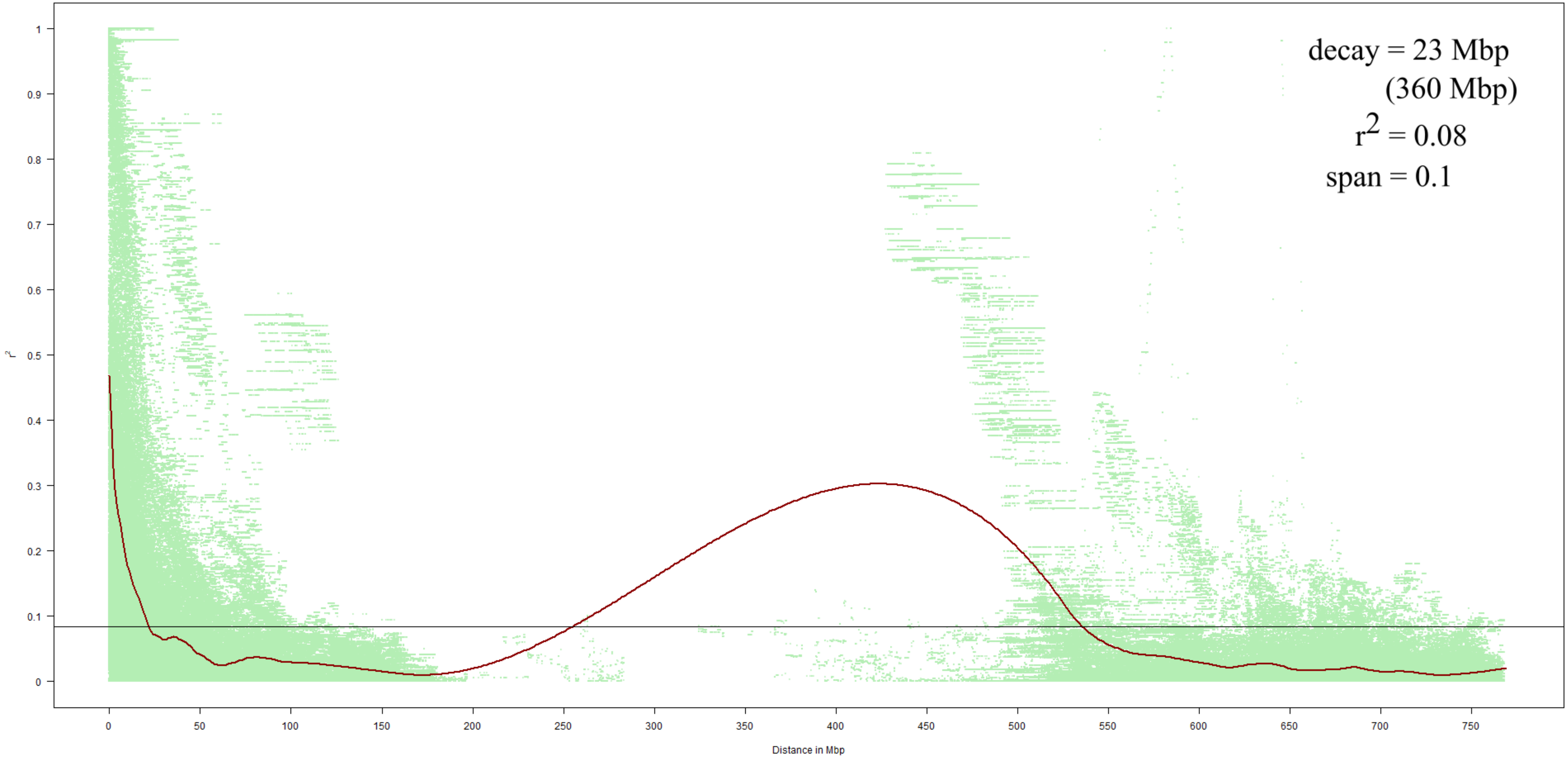

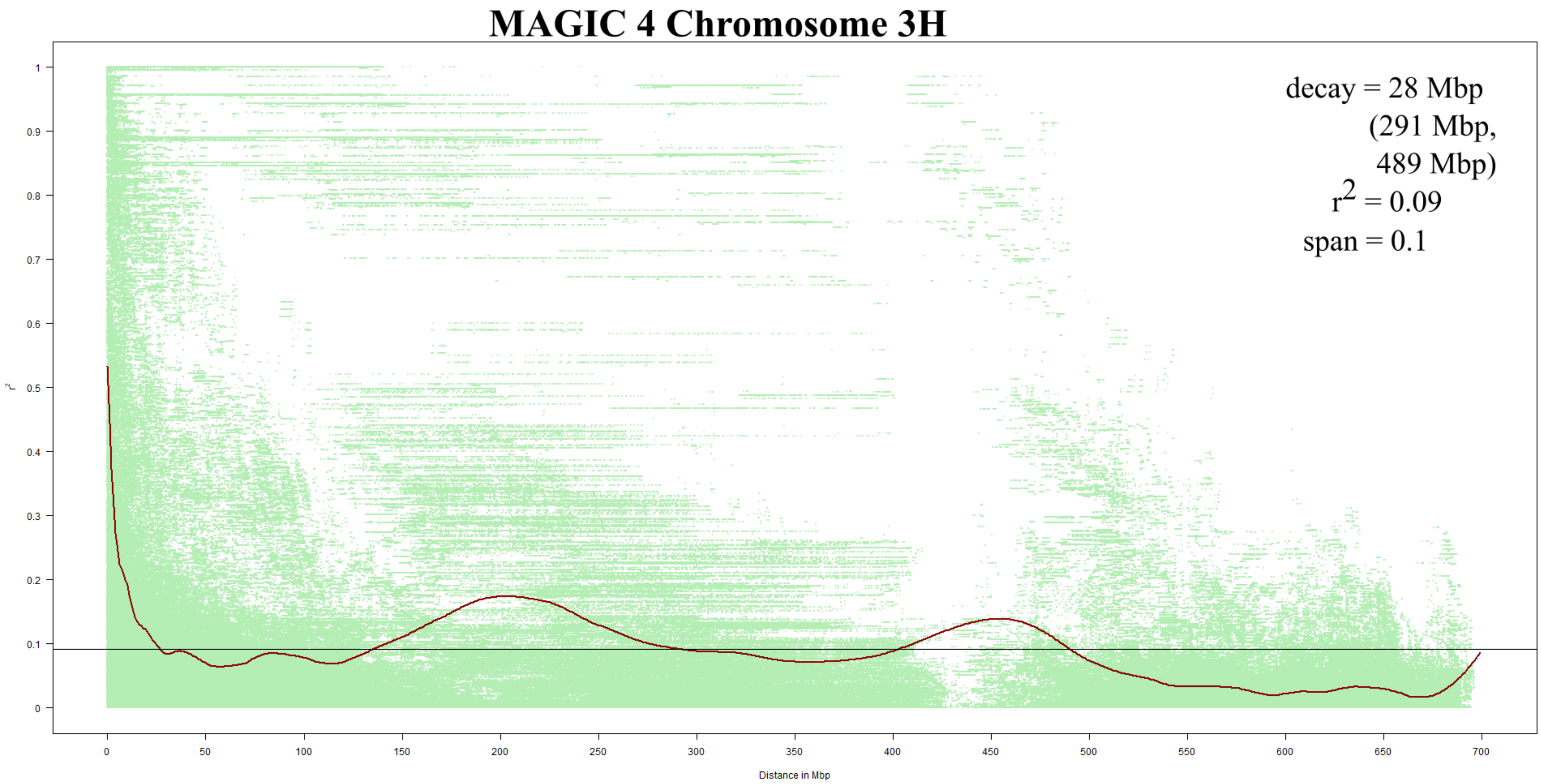
MAGIC 4 Chromosome 3H
decay = 28 Mbp
(291 Mbp,
489 Mbp)
r2 = 0.09
span = 0.1
r2
1
0.9
0.8
0.7
0.6
0.5
0.4
0.3
0.2
0.1
0
0
50
100
150
200
250
300
350
400
450
500
550
600
650
700
Distance in Mbp

MAGIC 4 Chromosome 4H

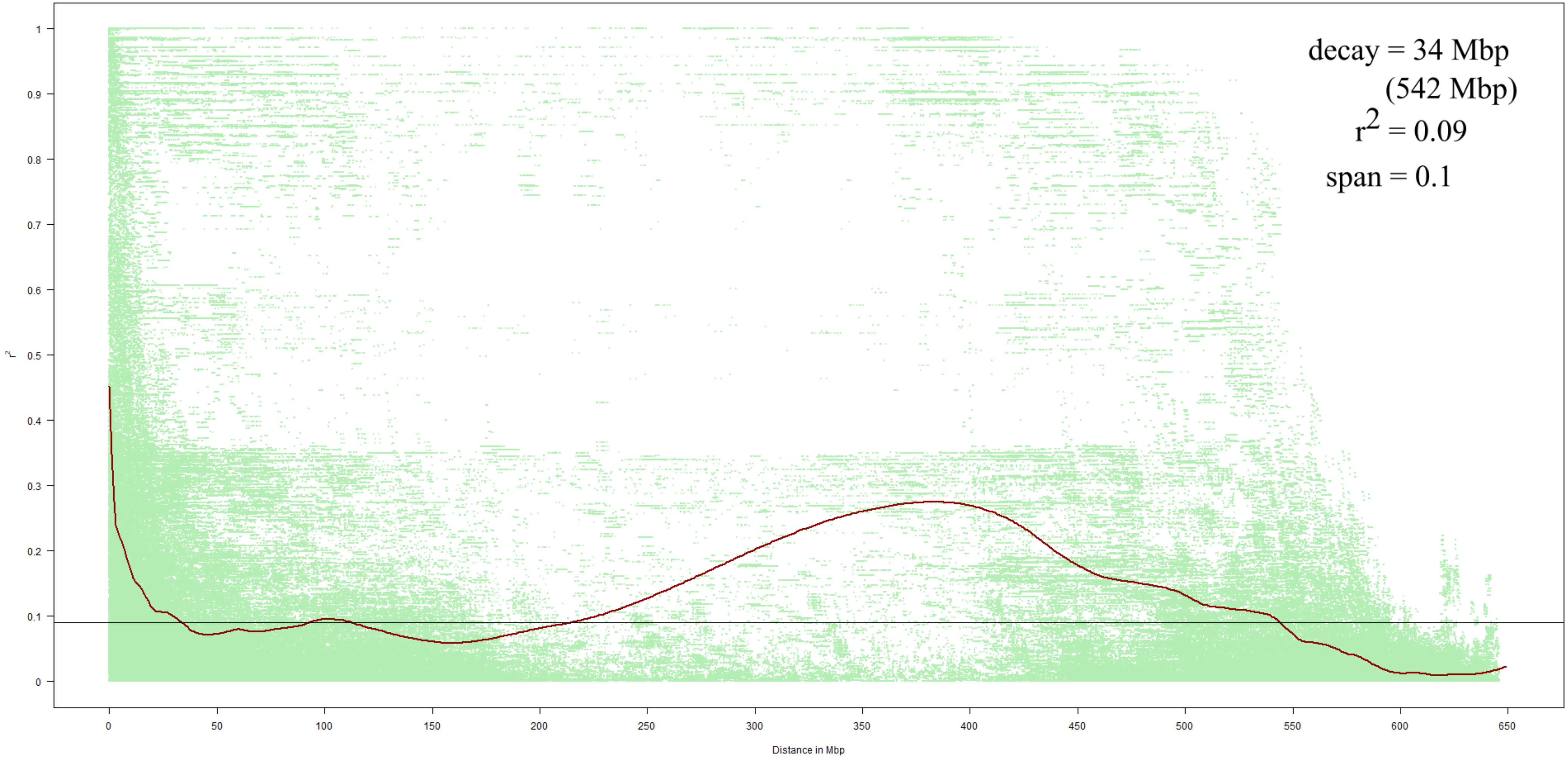

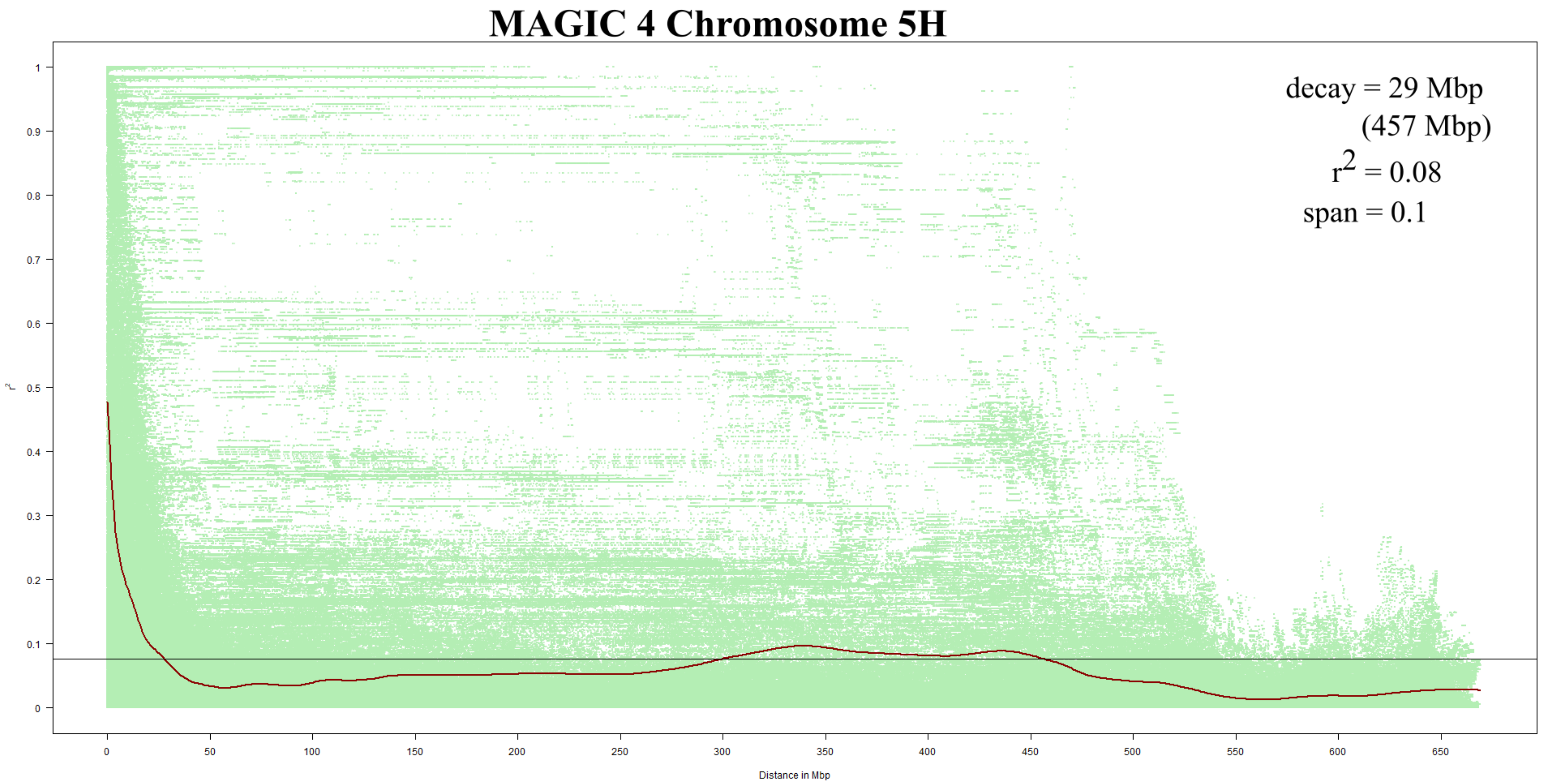

MAGIC 4 Chromosome 5H
decay = 29 Mbp
(457 Mbp)
r2 = 0.08
span = 0.1
r2
1
0.9
0.8
0.7
0.6
0.5
0.4
0.3
0.2
0.1
0
0
50
100
150
200
250
300
350
400
450
500
550
600
650
Distance in Mbp

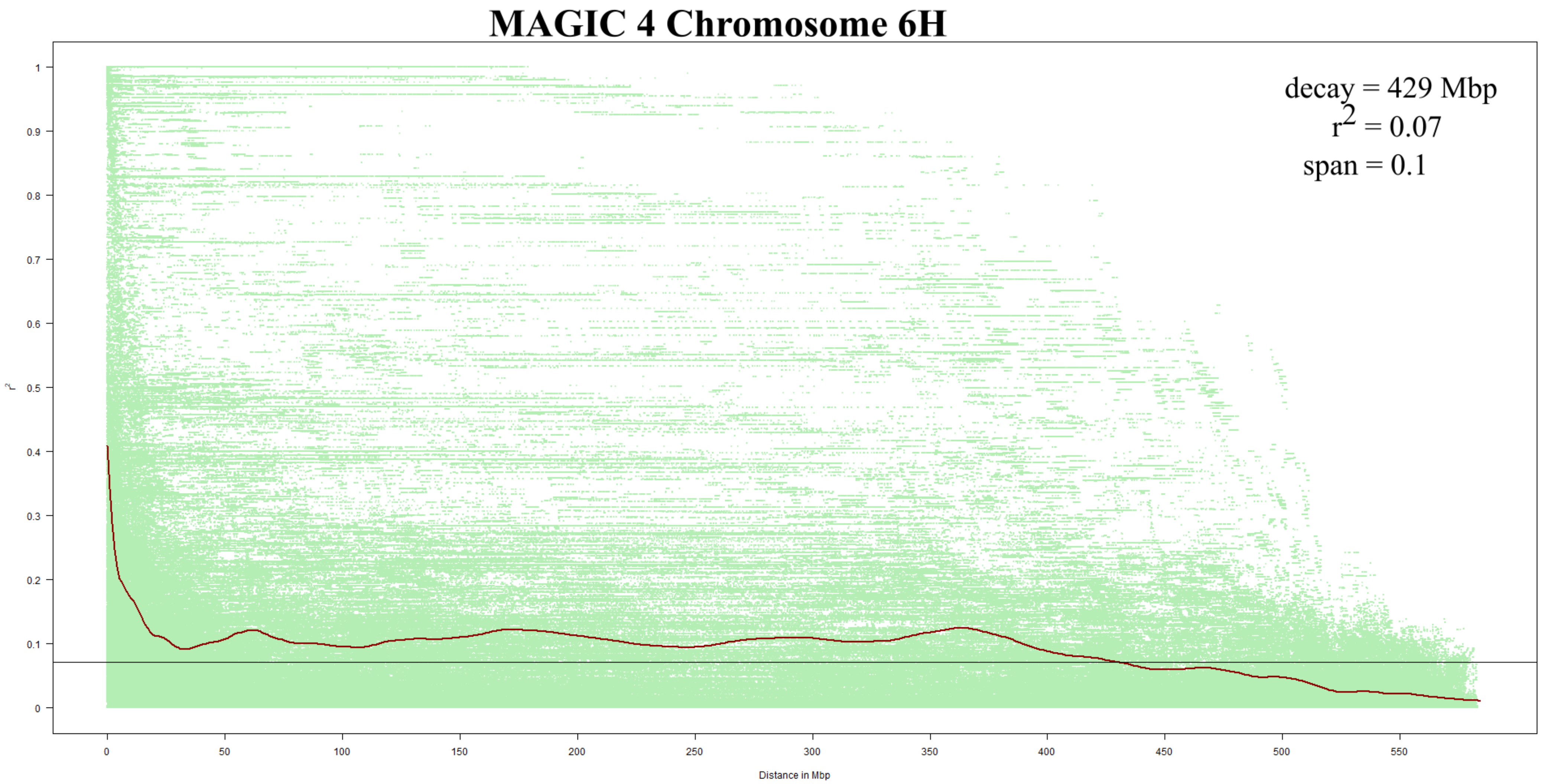
MAGIC 4 Chromosome 6H
decay = 429 Mbp
r2 = 0.07
span = 0.1
r2
Distance in Mbp
0
0.1
0.2
0.3
0.4
0.5
0.6
0.7
0.8
0.9
1
0
50
100
150
200
250
300
350
400
450
500
550

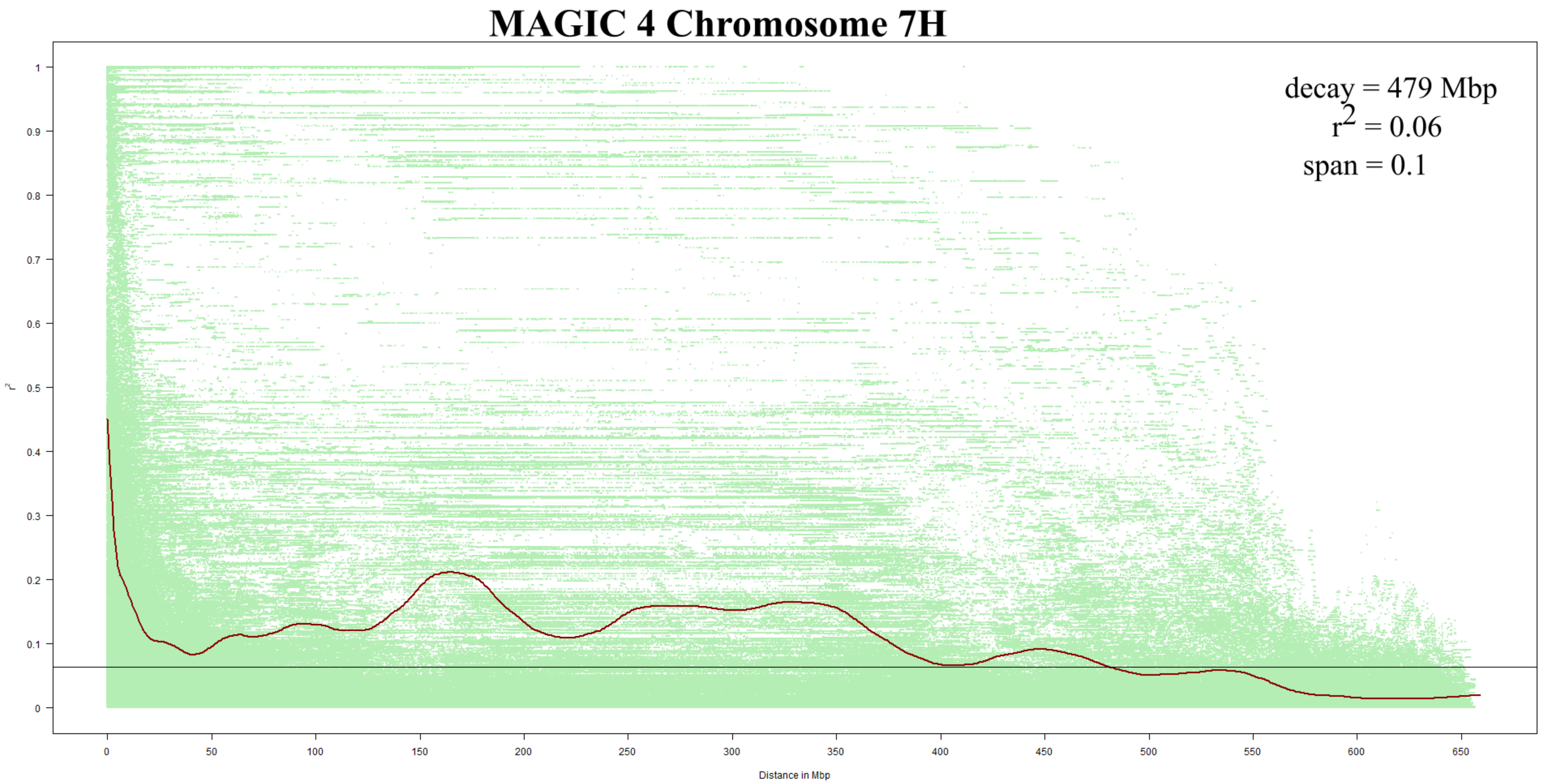
MAGIC 4 Chromosome 7H
decay = 479 Mbp
r^2 = 0.06
span = 0.1
r^2
1
0.9
0.8
0.7
0.6
0.5
0.4
0.3
0.2
0.1
0
0
50
100
150
200
250
300
350
400
450
500
550
600
650
Distance in Mbp

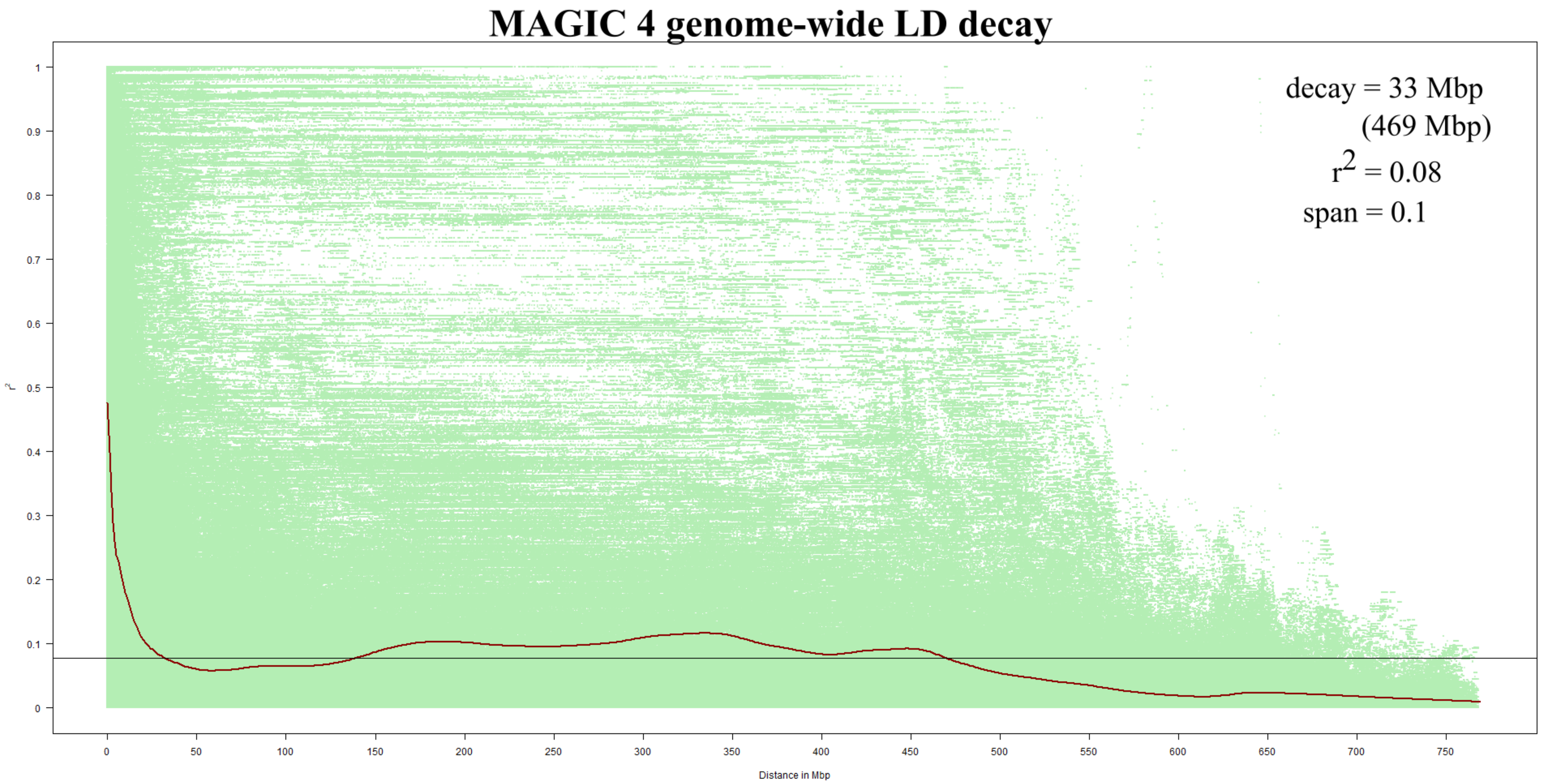

MAGIC 4 genome-wide LD decay
decay = 33 Mbp
(469 Mbp)
r^2 = 0.08
span = 0.1
r^2
0
0.1
0.2
0.3
0.4
0.5
0.6
0.7
0.8
0.9
1
0
50
100
150
200
250
300
350
400
450
500
550
600
650
700
750
Distance in Mbp

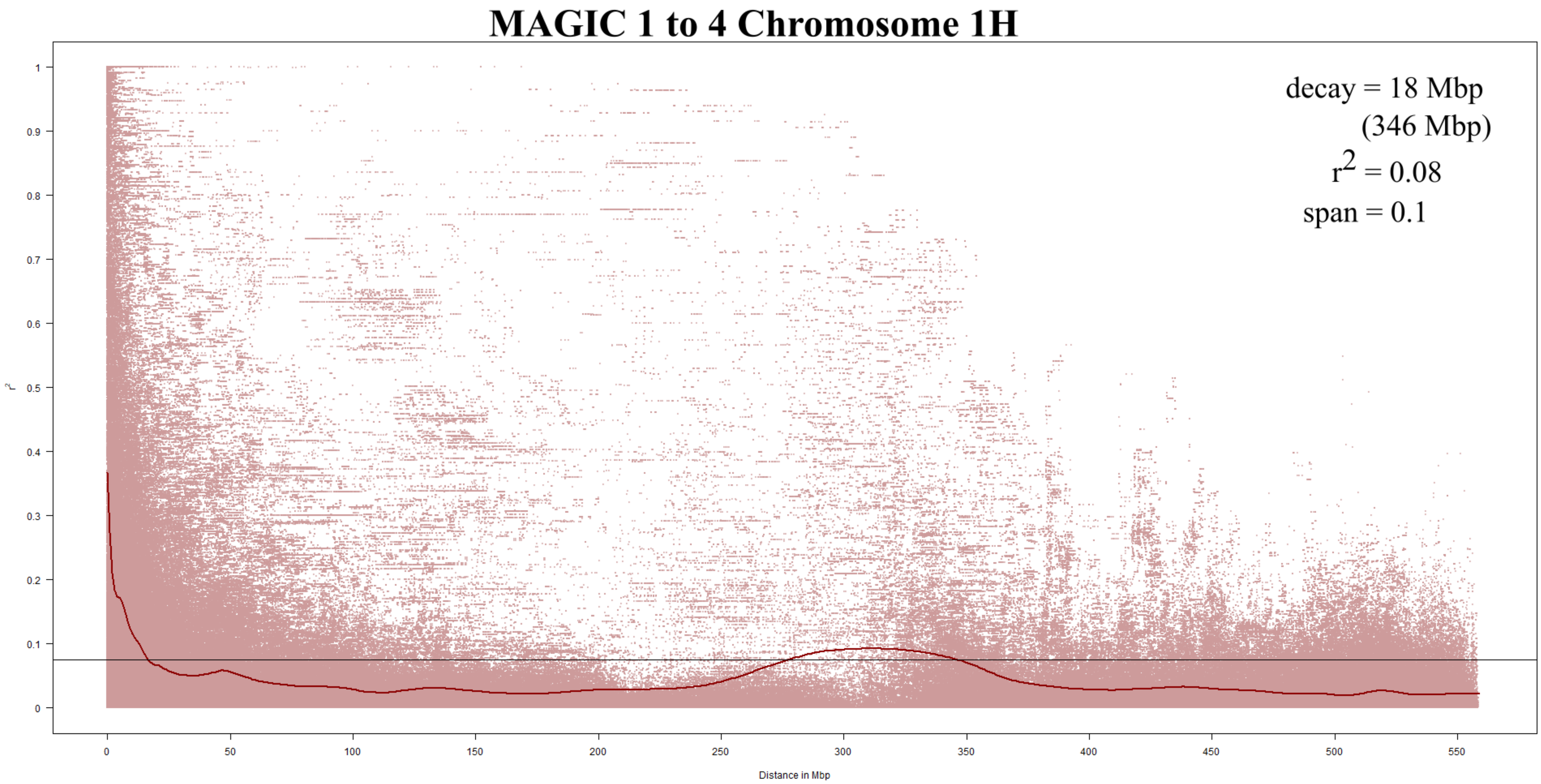
MAGIC 1 to 4 Chromosome 1H
decay = 18 Mbp
(346 Mbp)
r2 = 0.08
span = 0.1
r2
1
0.9
0.8
0.7
0.6
0.5
0.4
0.3
0.2
0.1
0
0
50
100
150
200
250
300
350
400
450
500
550
Distance in Mbp

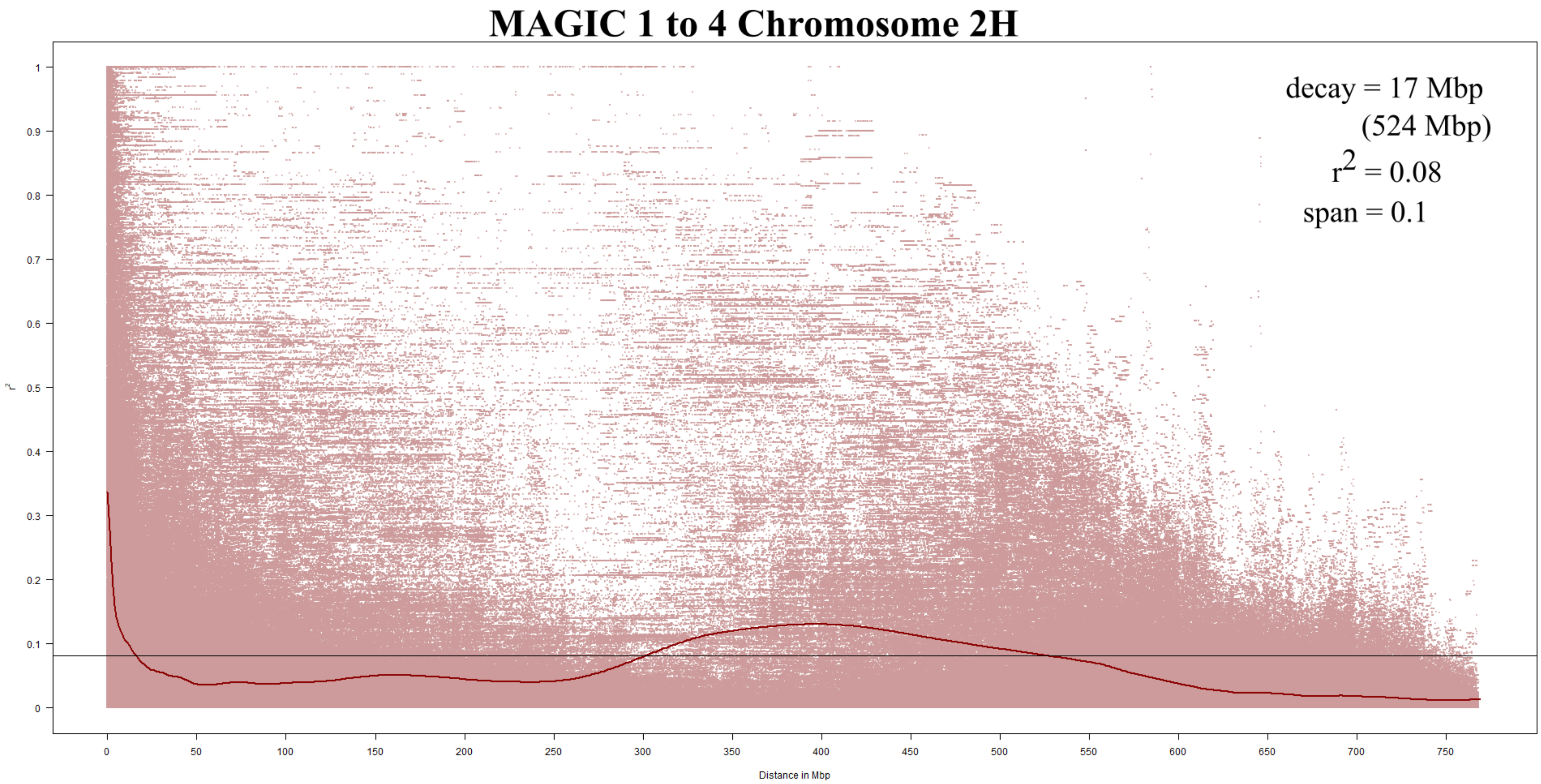

MAGIC 1 to 4 Chromosome 2H
decay = 17 Mbp
(524 Mbp)
r2 = 0.08
span = 0.1
r2
Distance in Mbp
0
0.1
0.2
0.3
0.4
0.5
0.6
0.7
0.8
0.9
1
0
50
100
150
200
250
300
350
400
450
500
550
600
650
700
750

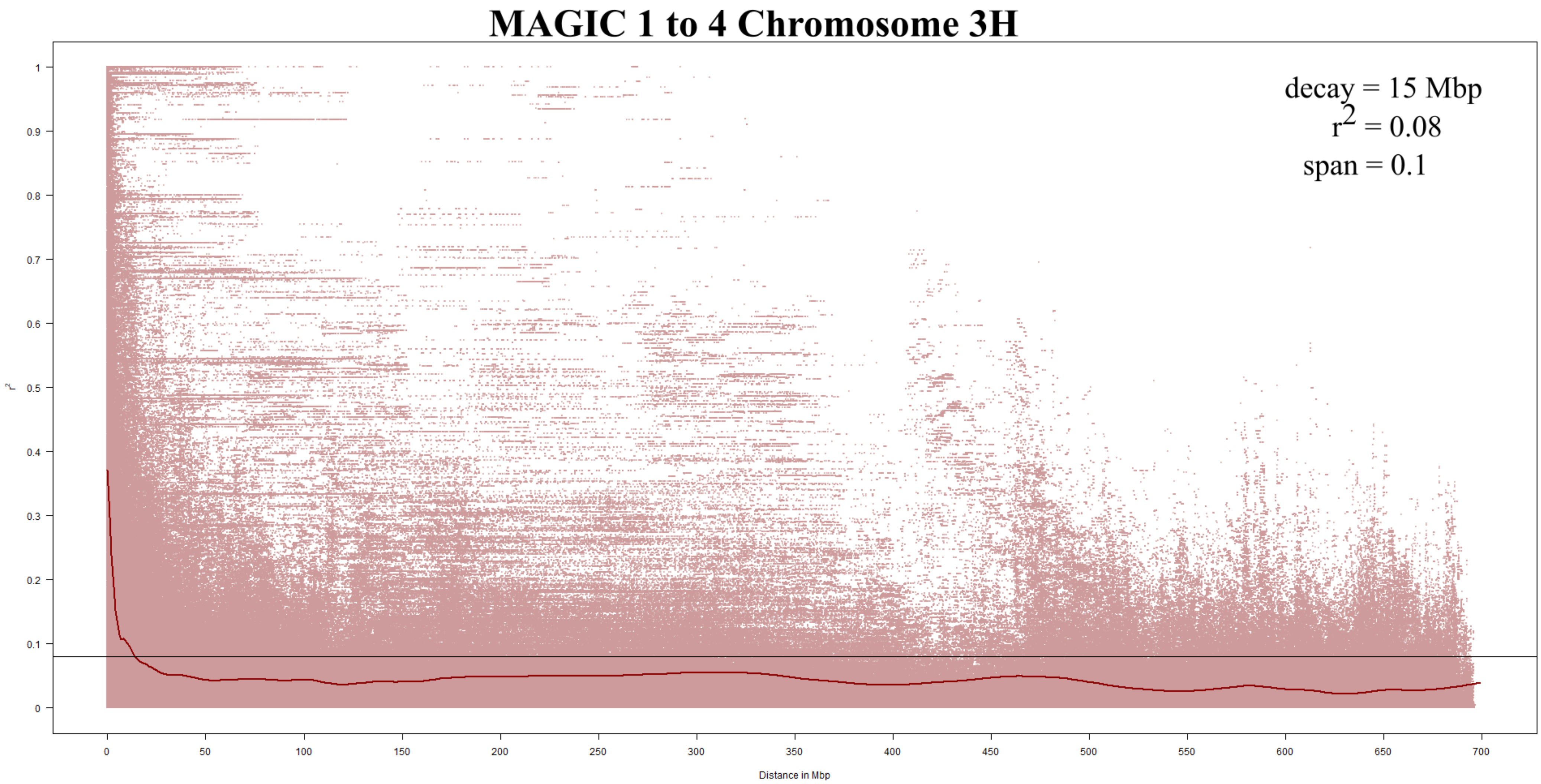

MAGIC 1 to 4 Chromosome 3H
decay = 15 Mbp
r2 = 0.08
span = 0.1
r2
1
0.9
0.8
0.7
0.6
0.5
0.4
0.3
0.2
0.1
0
0
50
100
150
200
250
300
350
400
450
500
550
600
650
700
Distance in Mbp

MAGIC 1 to 4 Chromosome 4H

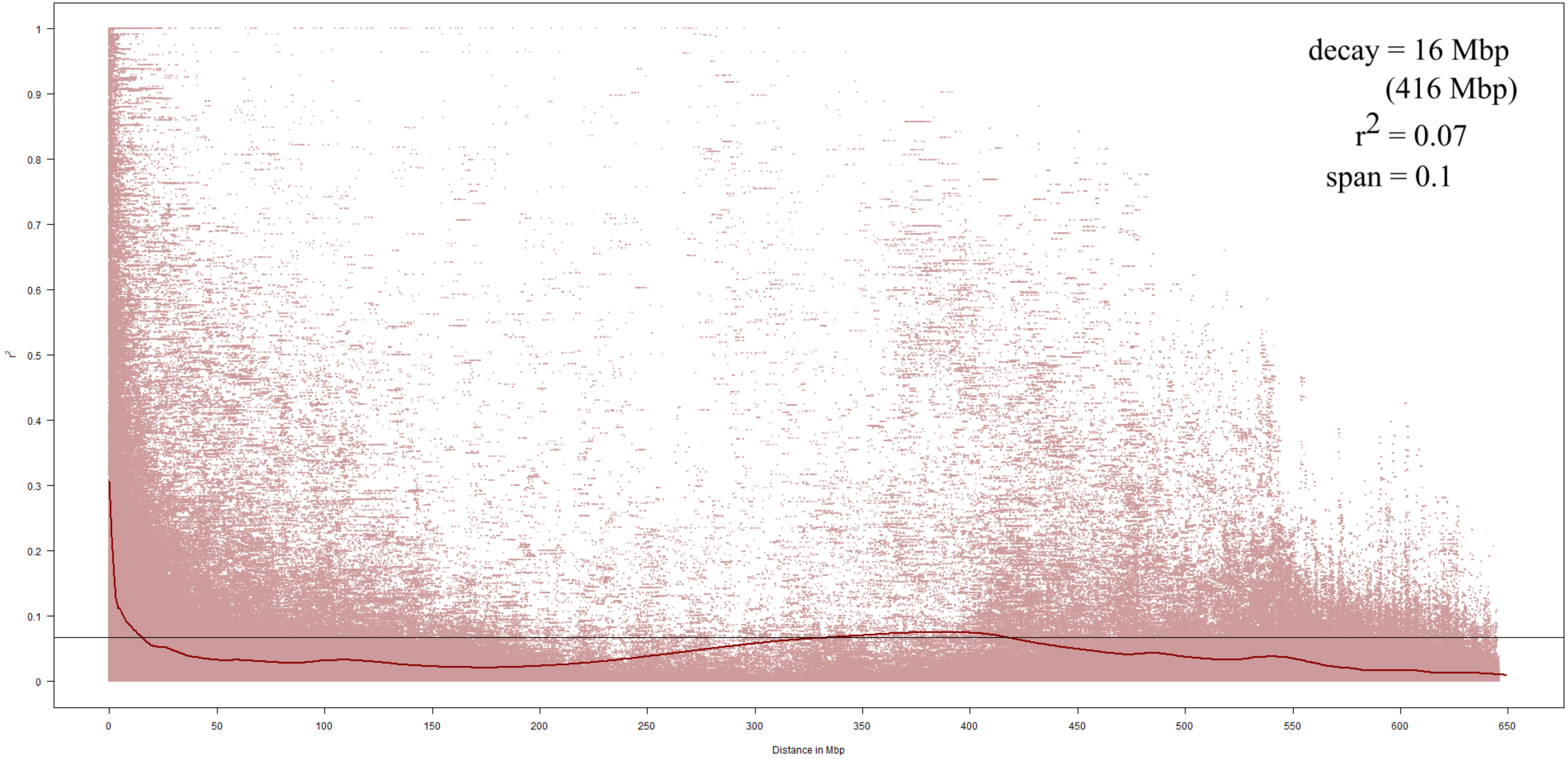

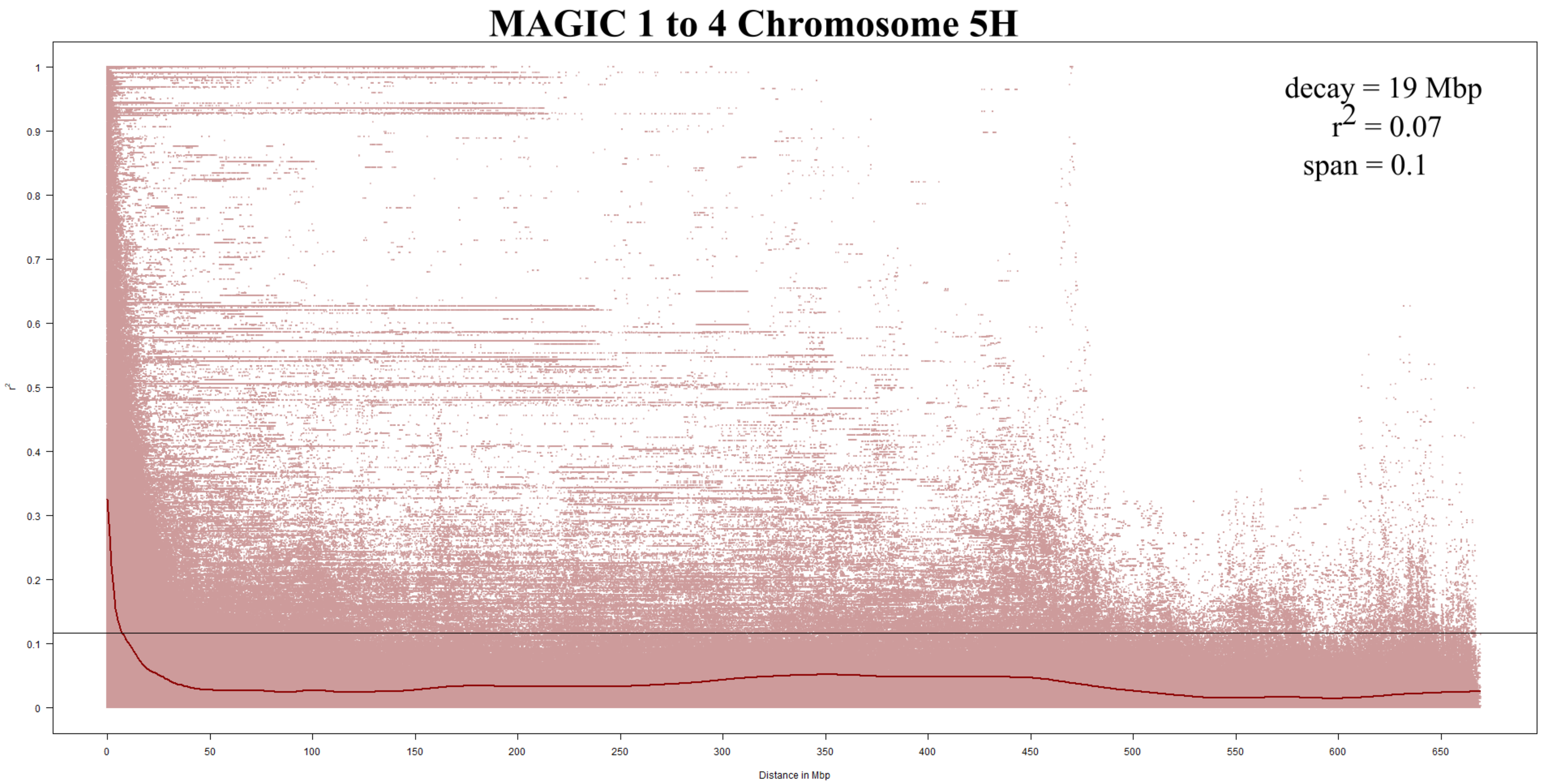

MAGIC 1 to 4 Chromosome 5H
decay = 19 Mbp
r2 = 0.07
span = 0.1
r2
0
0.1
0.2
0.3
0.4
0.5
0.6
0.7
0.8
0.9
1
0
50
100
150
200
250
300
350
400
450
500
550
600
650
Distance in Mbp

MAGIC 1 to 4 Chromosome 6H

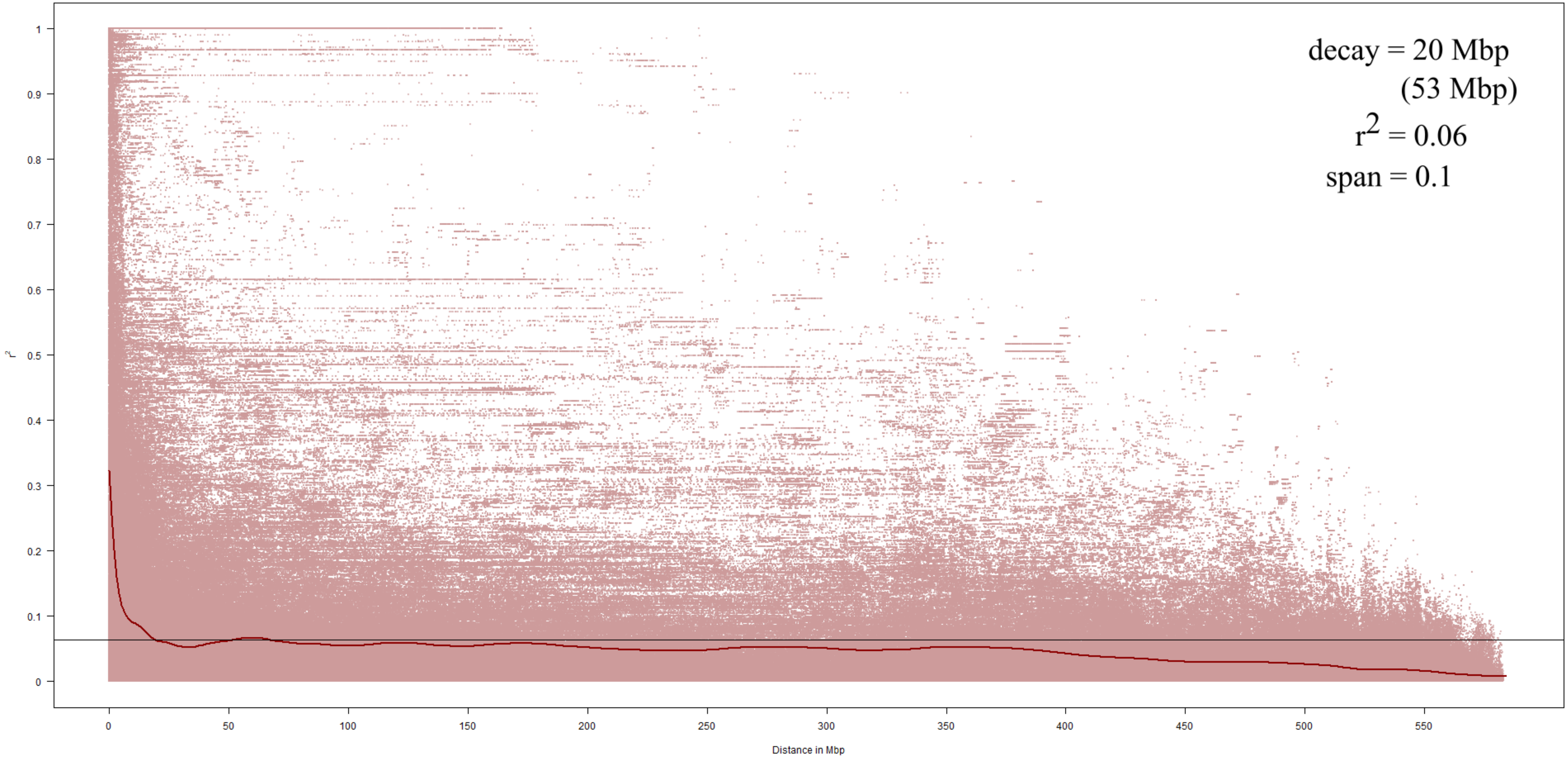

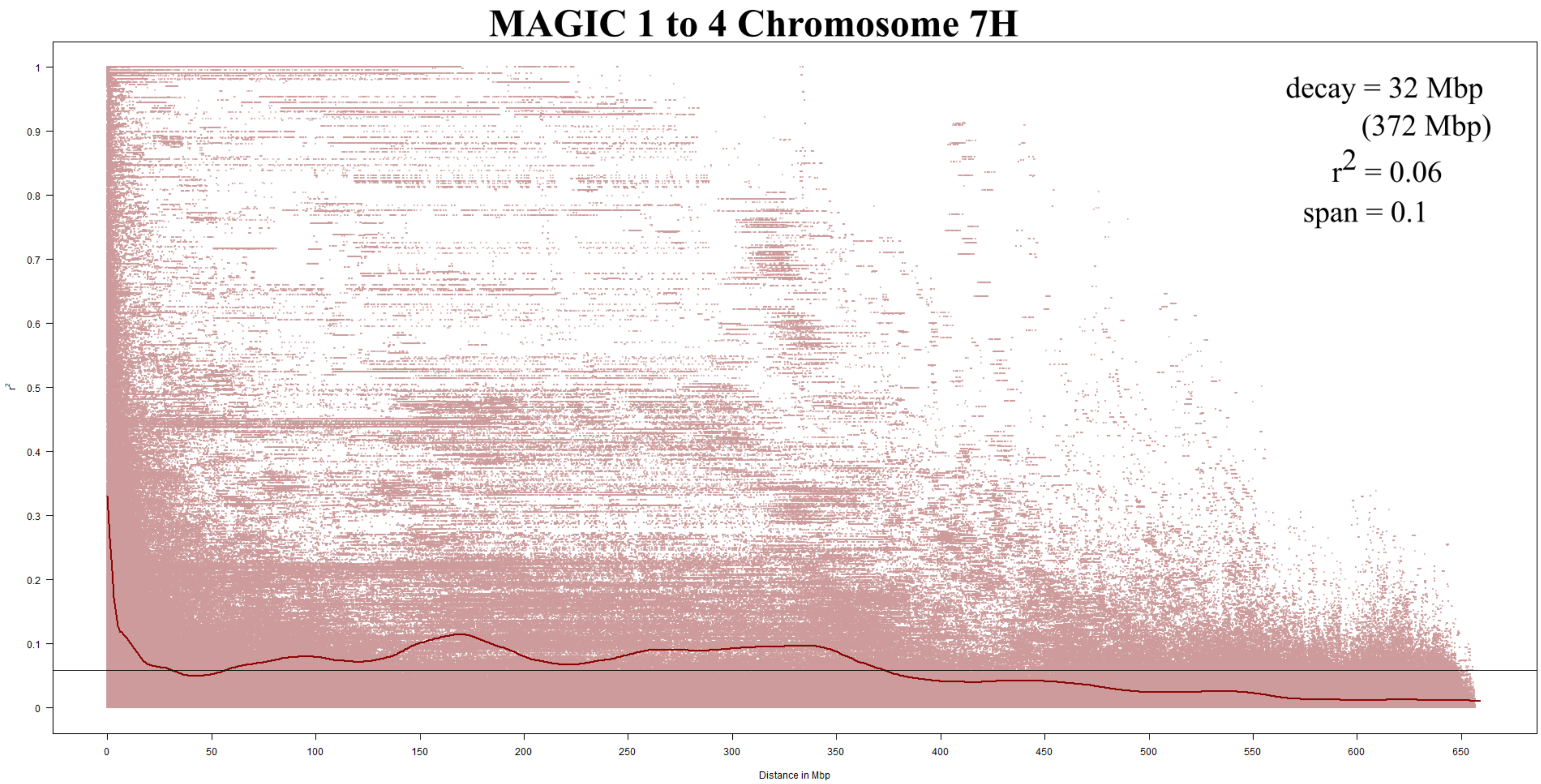

MAGIC 1 to 4 Chromosome 7H
decay = 32 Mbp
(372 Mbp)
r2 = 0.06
span = 0.1
r2
Distance in Mbp
