# Supplementary material for: You Had Me at “MAGIC”!: Four Barley MAGIC Populations Reveal Novel Resistance QTL for Powdery Mildew
Source: Genes (Basel). 2020 Dec 18;11(12):1512. doi: 10.3390/genes11121512 (PMC7766815; doi:10.3390/genes11121512)
Supplement: Supplementary file 1 [file genes-11-01512-s001.zip › Figure S4.pdf]

Figure S4

You had me at "MAGIC"! Four barley MAGIC populations reveal novel resistance QTL for Powdery Mildew

Fluturë Novakazi [1], Lene Krusell [2], Jens Due Jensen [3], Jihad Orabi [3], The PPP Barley Consortium, Ahmed Jahoor [3], Therése Bengtsson [1*]

[1] Department of Plant Breeding, Swedish University of Agricultural Sciences, Box 101, 230 53, Alnarp Sweden

[2] Sejet Plant Breeding, Nørremarksvej 67, 8700 Horsens, Denmark

[3] Nordic Seed A/S, Kornmarken 1, 8464 Galten Denmark

*Correspondence: Therése Bengtsson: therese.bengtsson@slu.se

Content

Manhattan and QQ-plots for each individual MAGIC population and the combined panel (MAGIC 1 to 4) for different models and covariate combinations.

Models tested:

GLM – General Linear Model (Zhang et al. 2010)

MLM – Mixed Linear Model (Zhang et al. 2010)

MLMM – Multiple Loci Mixed linear Model (Segura et al. 2012)

FarmCPU – Fixed and random model Circulating Probability Unification (Liu et al. 2016)

Covariates used:

K – Kinship matrix based on VanRaden Method (Van Raden 2008)

Q – Ancestry coefficient data obtained from STRUCTURE analysis (Pritchard et al. 2000)

The PPP Barley Consortium
Inger Åhman (Department of Plant Breeding, Swedish University of Agricultural Sciences, Box 101, 230 53 Alnarp, Sweden),
Therése Bengtsson (Department of Plant Breeding, Swedish University of Agricultural Sci-ences, Box 101, 230 53 Alnarp, Sweden),
Fluturë Novakazi (Department of Plant Breeding, Swedish University of Agricultural Scienc-es, Box 101, 230 53 Alnarp, Sweden),
Outi Manninen (Boreal Plant Breeding Ltd, Myllytie 10, 31600 Jokioinen, Finland),
Merja Veteläinen (Boreal Plant Breeding Ltd, Myllytie 10, 31600 Jokioinen, Finland),
Mika Isolahti, (Boreal Plant Breeding Ltd, Myllytie 10, 31600 Jokioinen, Finland),
Muath Alsheikh (Graminor A/S, Hommelstadvegen 60, 2322 Ridabu, Norway),
Stein Bergersen (Graminor A/S, Hommelstadvegen 60, 2322 Ridabu, Norway),
Constantin Jansen (Graminor A/S, Hommelstadvegen 60, 2322 Ridabu, Norway),
Susanne Windju (Graminor A/S, Hommelstadvegen 60, 2322 Ridabu, Norway),
Marja Jalli, (Natural Resources Institute Finland (Luke), Tietotie 4, 31600 Jokioinen, Finland),
Juho Hautsalo, (Natural Resources Institute Finland (Luke), Survontie 9, 40500 Jyväskylä, Finland,
Ahmed Jahoor (NordicSeed A/S, Kornmarken 1, 8464 Galten, Denmark) (project leader),
Jens Due Jensen (Nordic Seed A/S, Kornmarken 1, 8464Galten, Denmark),
Jihad Orabi (Nordic Seed A/S, Kornmarken 1, 8464 Galten, Denmark),
Nana Vagndorf Nordestgaard (Nordic Seed A/S, Kornmarken 1, 8464 Galten, Denmark),
Lene Krusell (Sejet Plant Breeding, Nørremarksvej 67, 8700 Horsens, Denmark),
Rasmus Lund Hjortshøj (Sejet Plant Breeding, Nørremarksvej 67, 8700 Horsens, Denmark),
Charlotte Damgård Robertsen, (Sejet Plant Breeding, Nørremarksvej 67, 8700 Horsens, Den-mark),
Magnus Göransson (The Agricultural University of Iceland, Faculty of Land and Animal Re-sources, Hvanneyri, 311 Borgarnes, Iceland)

MAGIC 1+2

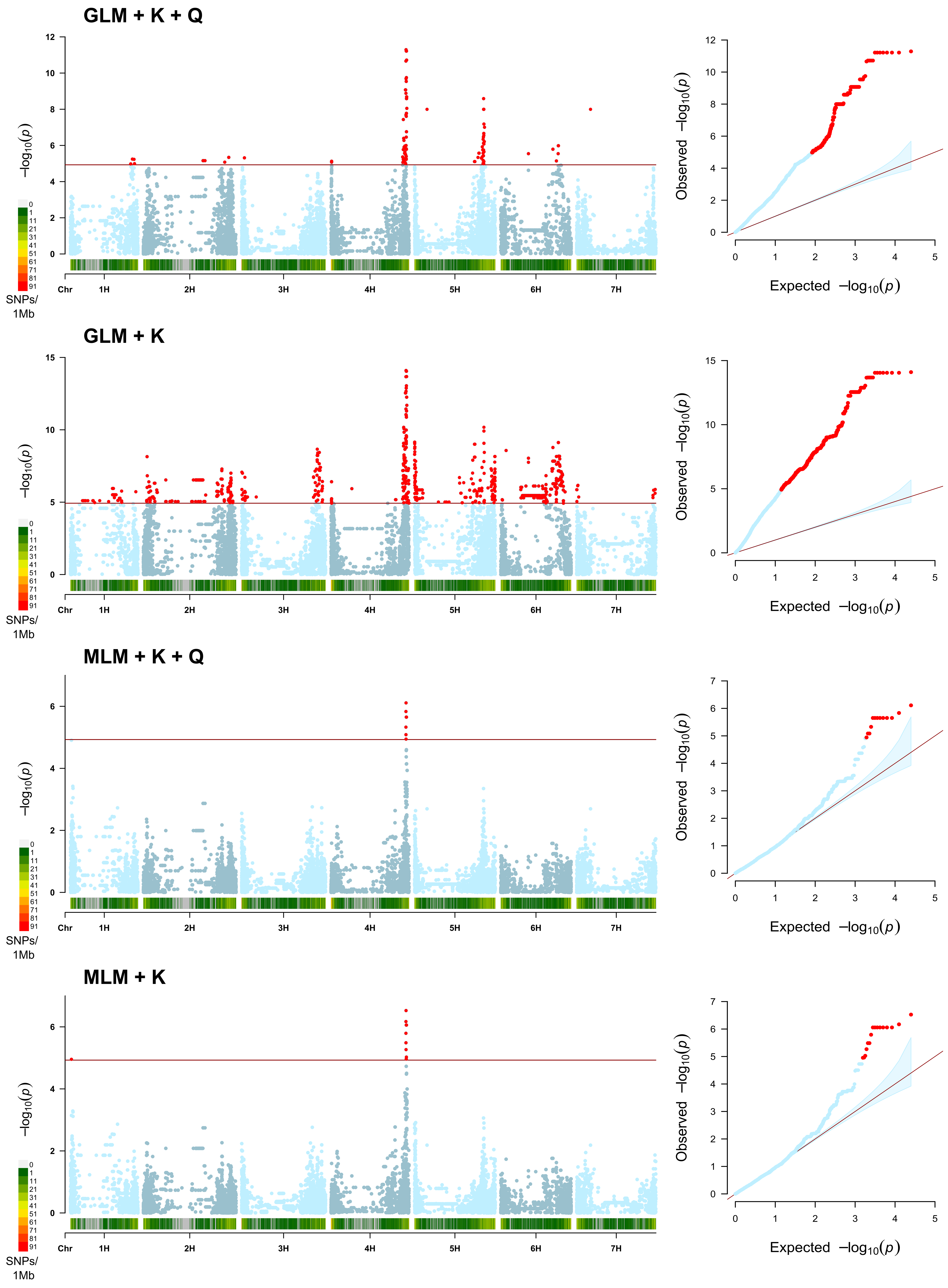
GLM + K + Q
GLM + K
MLM + K + Q
MLM + K
−log10(p)
Chr
1H
2H
3H
4H
5H
6H
7H
SNPs/
1Mb
Observed −log10(p)
Expected −log10(p)

MAGIC 1+2

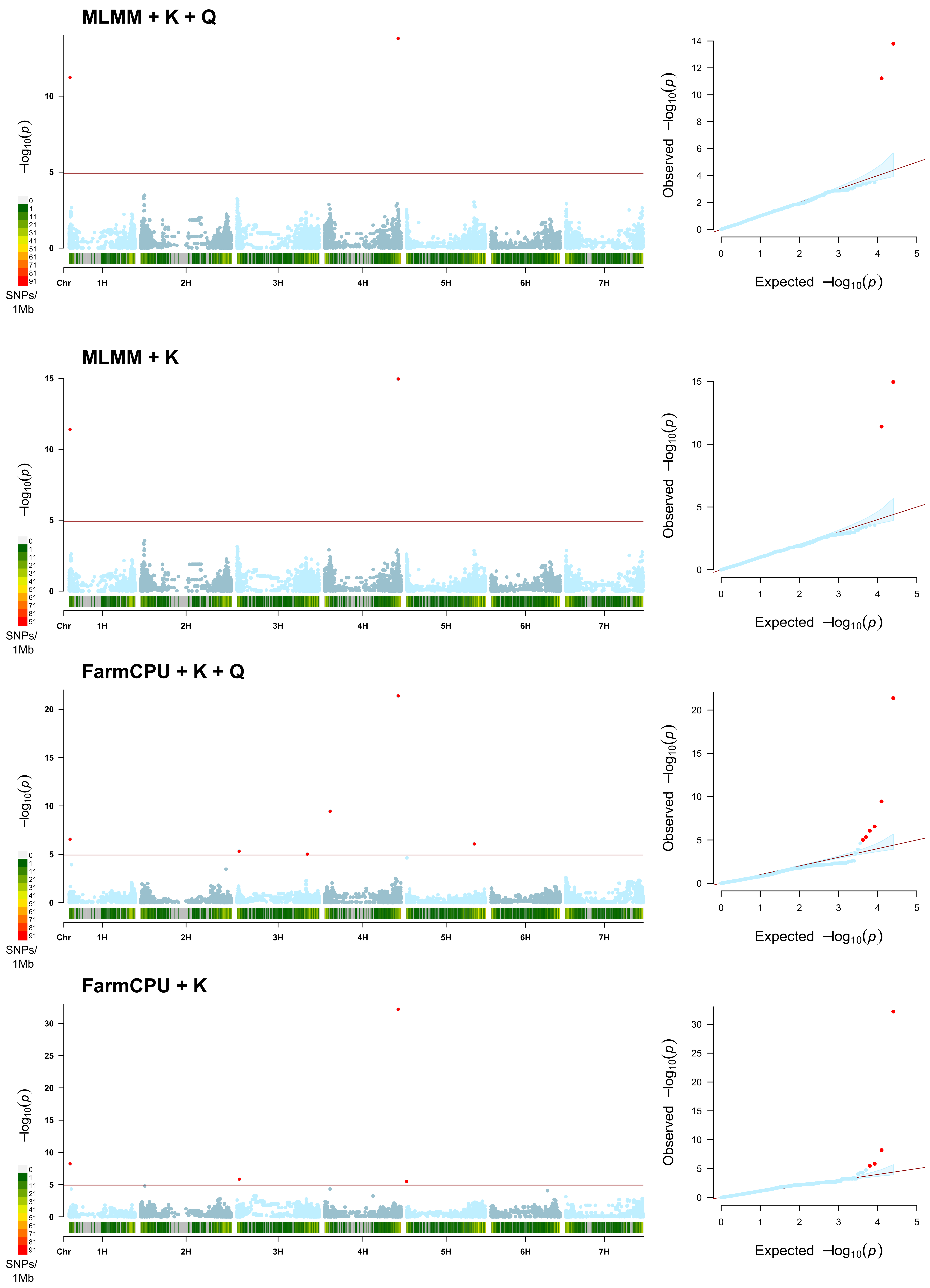
MLMM + K + Q
MLMM + K
FarmCPU + K + Q
FarmCPU + K
−log10(p)
SNPs/
1Mb
Chr
1H
2H
3H
4H
5H
6H
7H
Observed −log10(p)
Expected −log10(p)

MAGIC 3

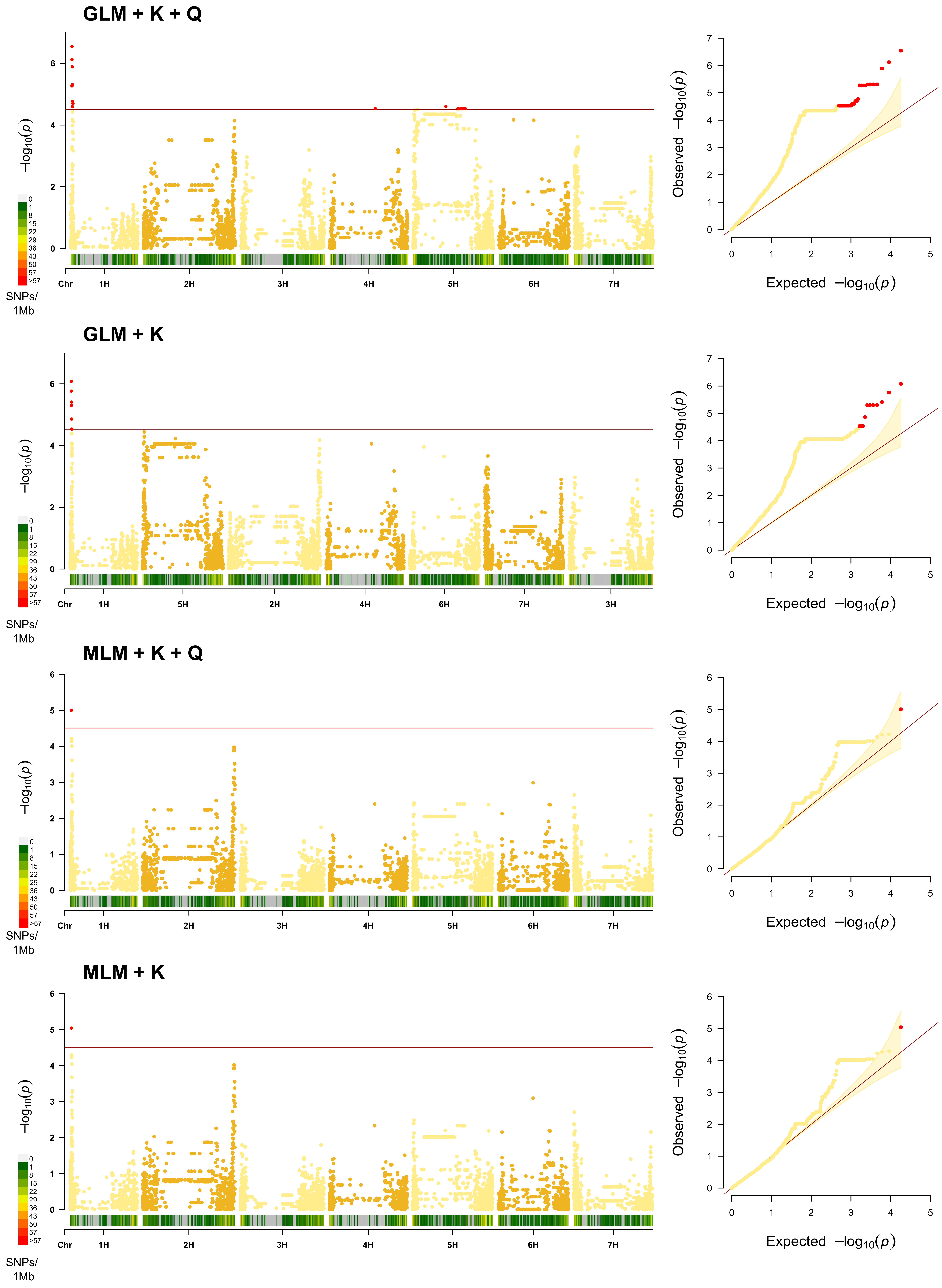
GLM + K + Q
GLM + K
MLM + K + Q
MLM + K
−log10(p)
SNPs/
1Mb
0
1
8
15
22
29
36
43
50
57
>57
Chr
1H
2H
3H
4H
5H
6H
7H
Observed −log10(p)
Expected −log10(p)

MAGIC 3

MLMM + K + Q

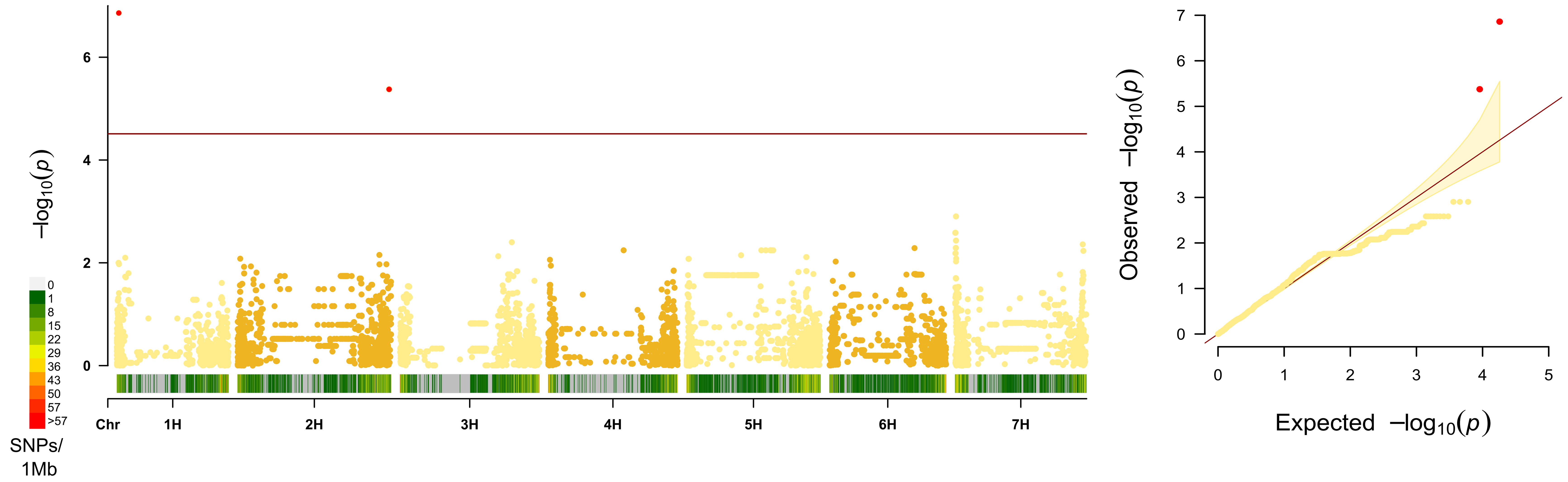

MLMM + K

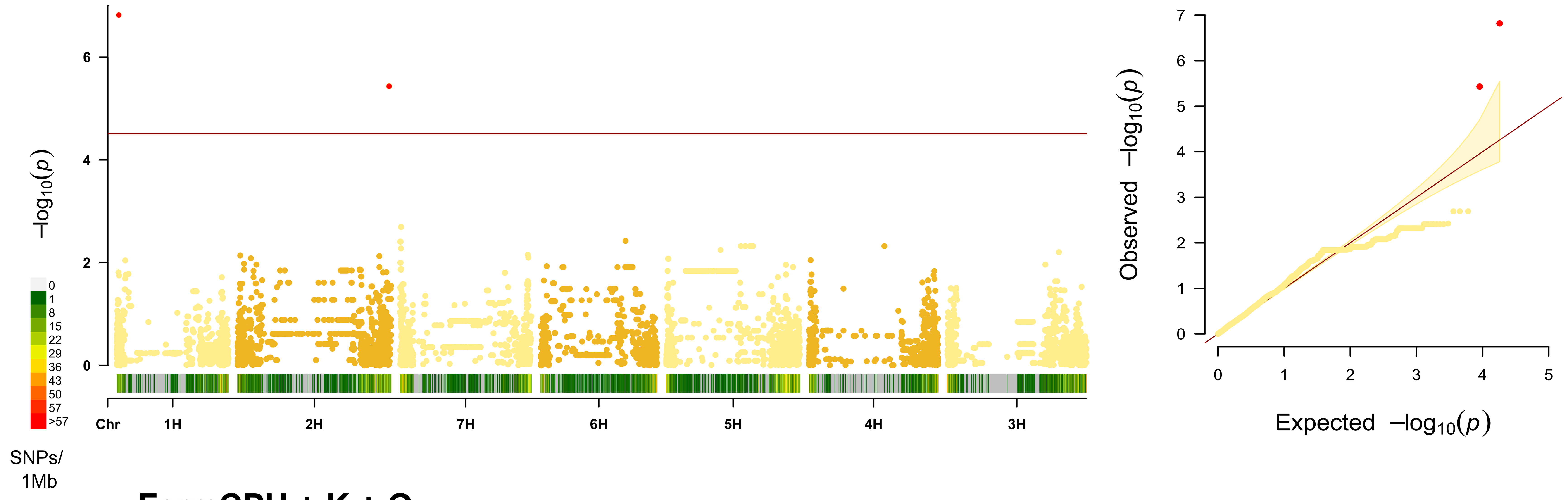

FarmCPU + K + Q

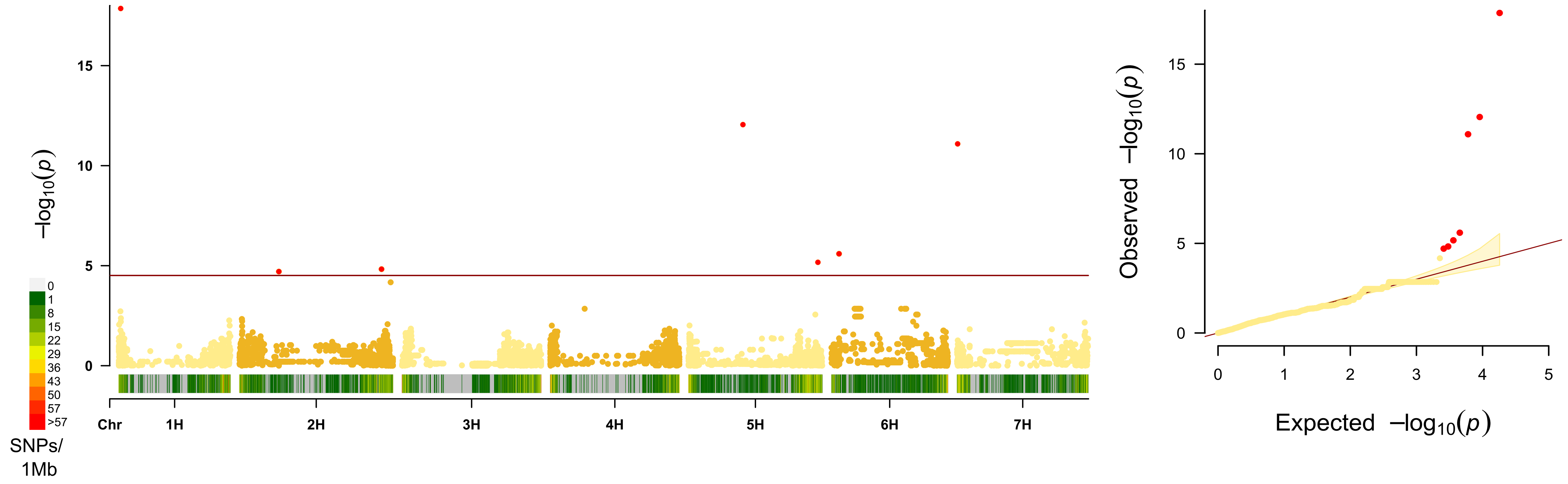

FarmCPU + K

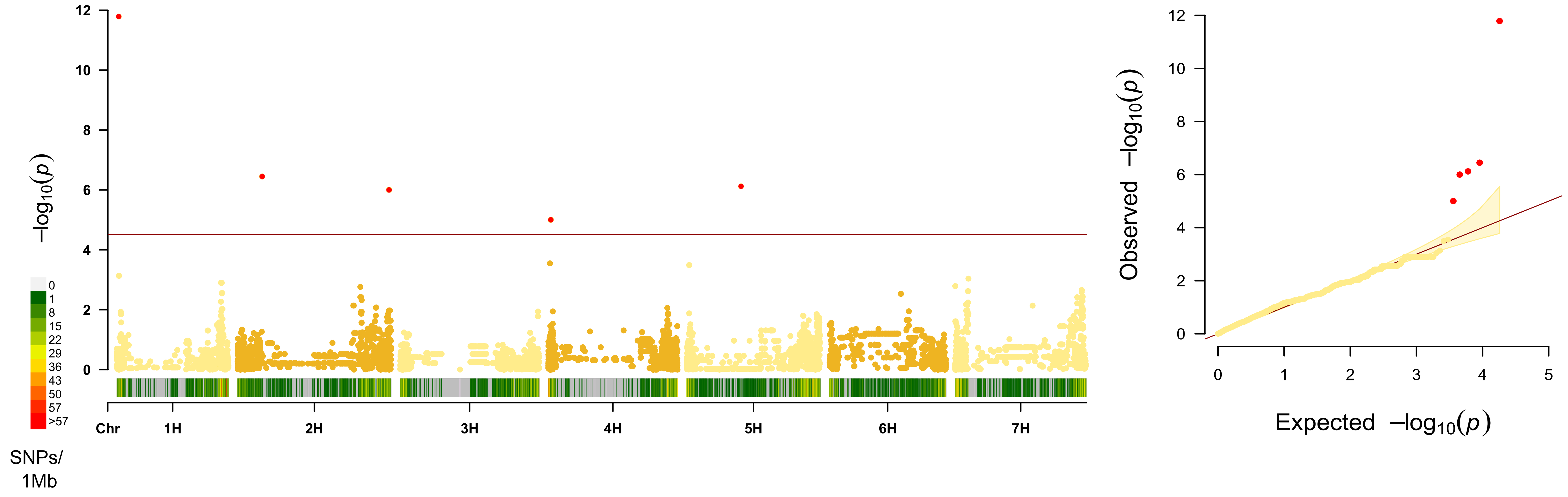

MAGIC 4

GLM + K + Q

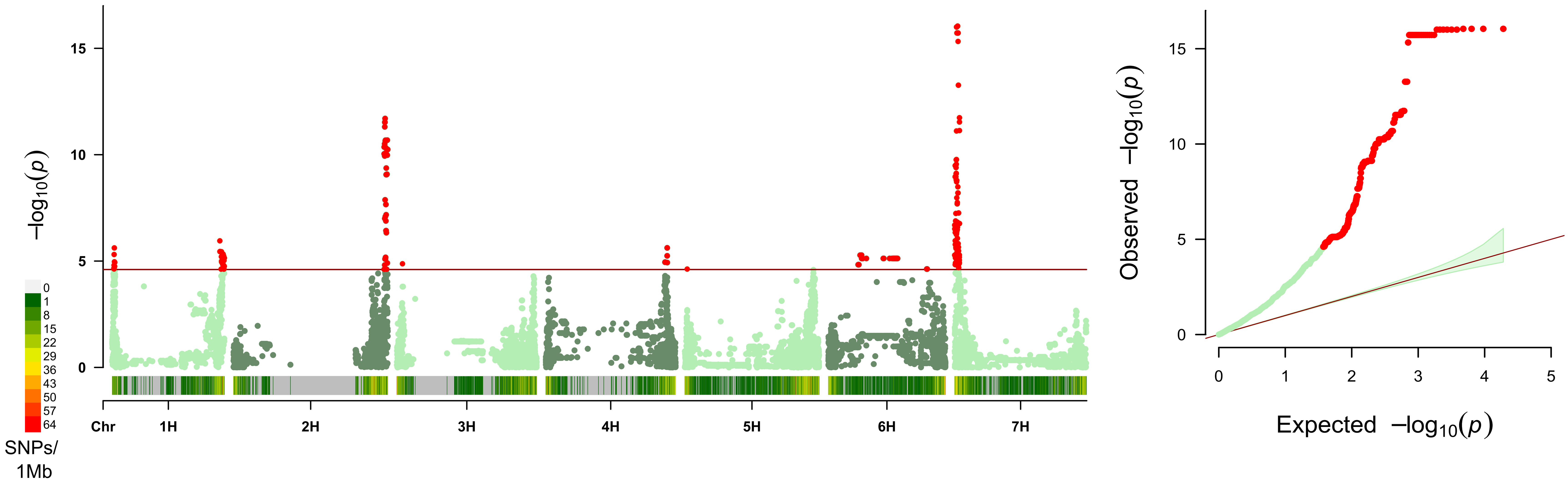

GLM + K

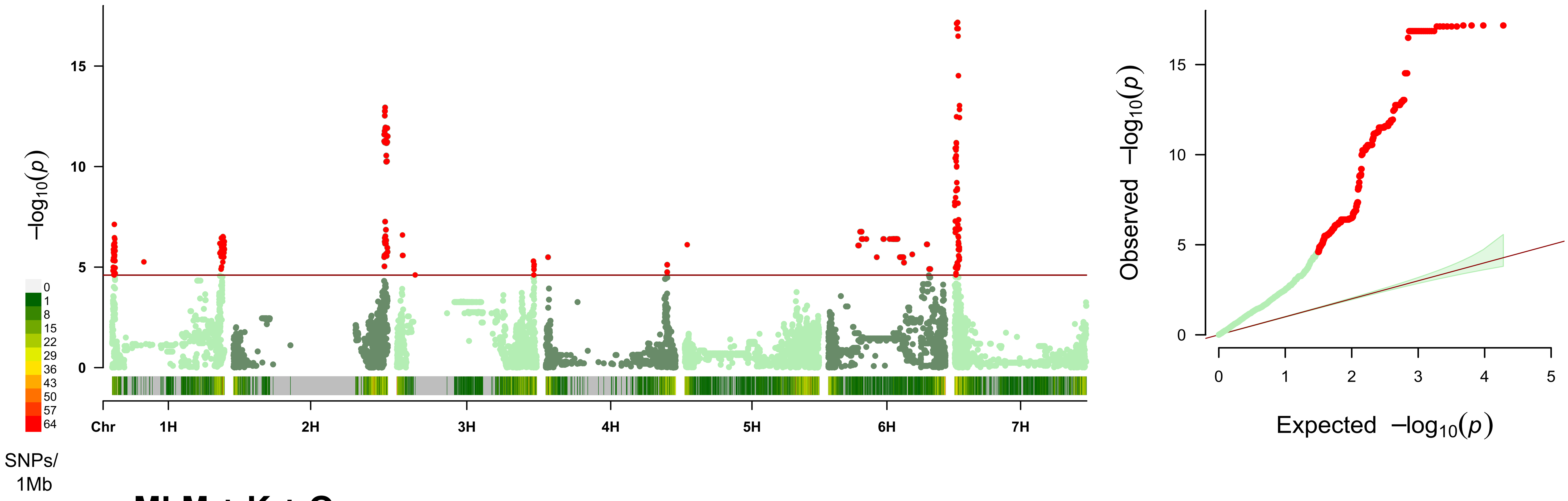

MLM + K + Q

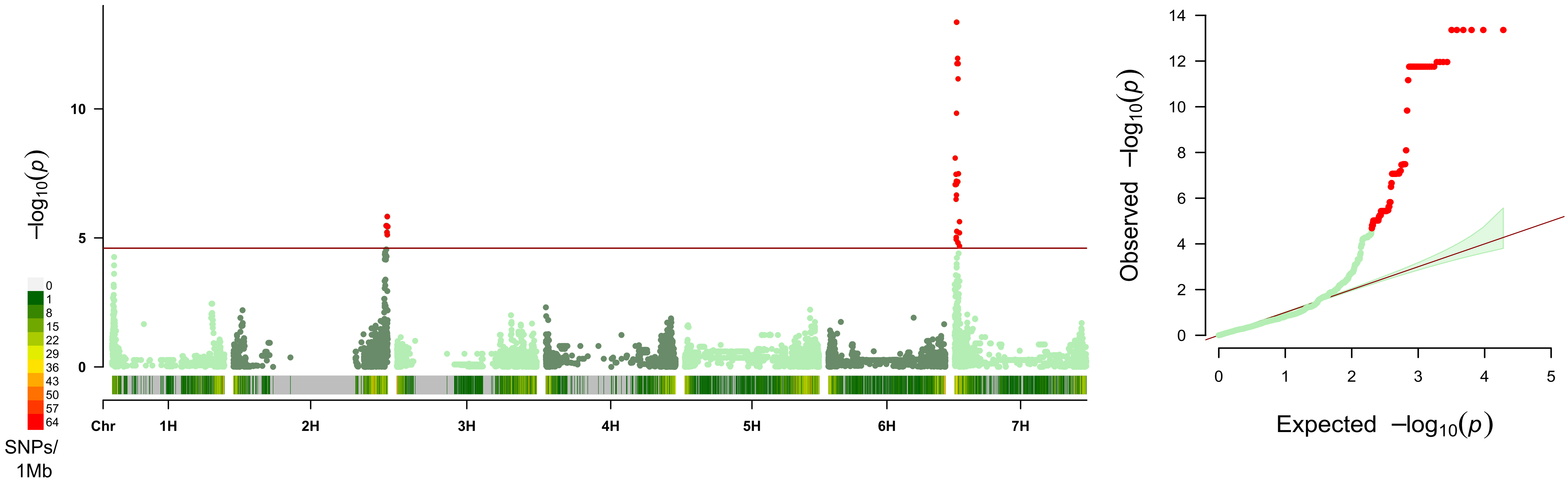

MLM + K

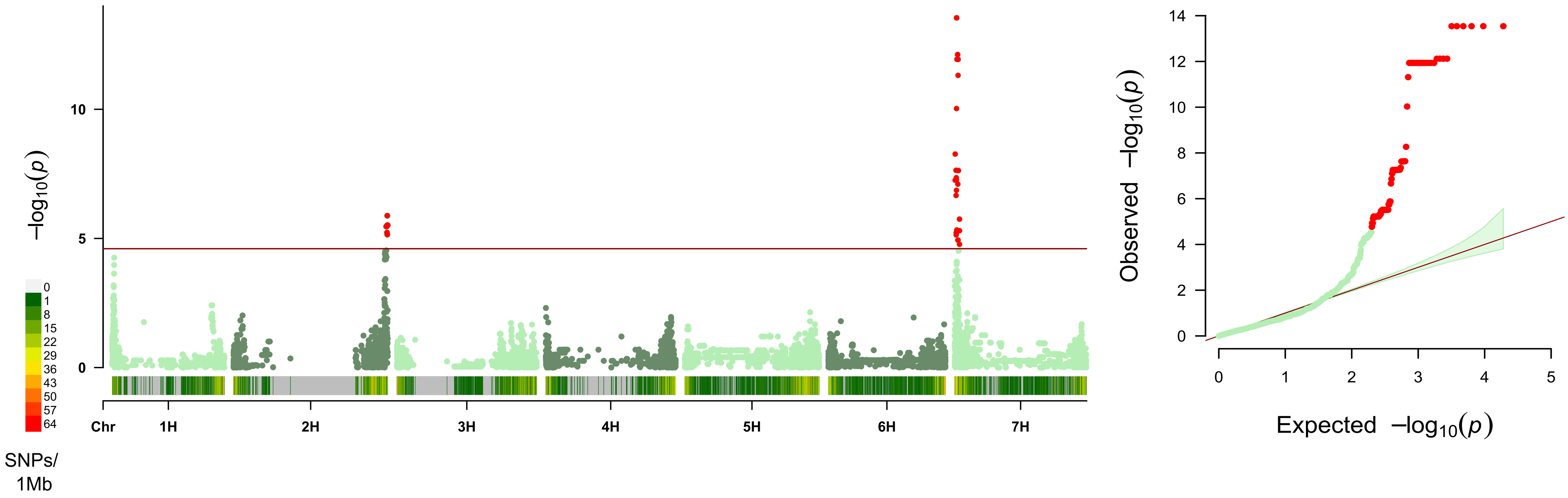

MAGIC 4

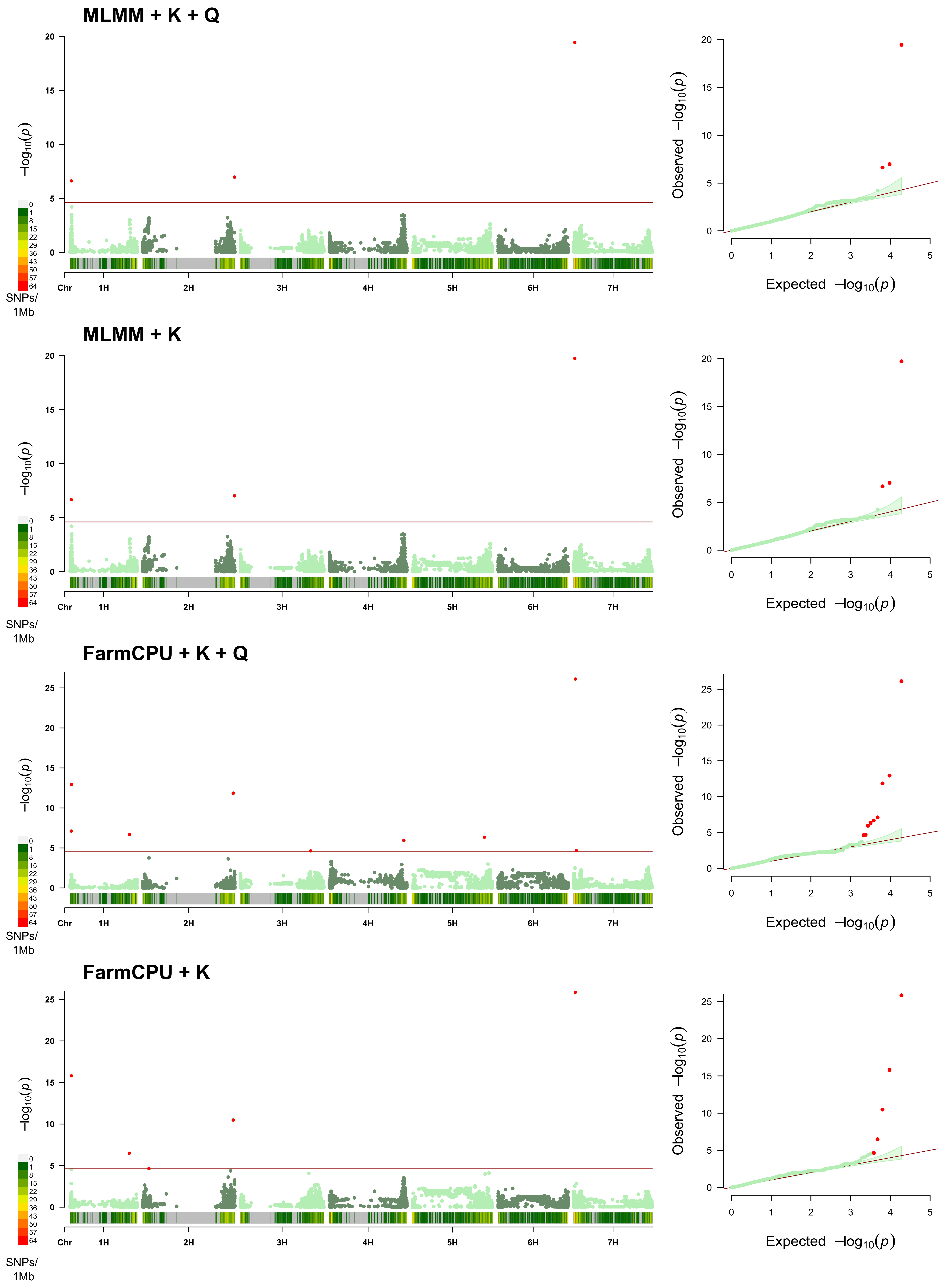
MLMM + K + Q
MLMM + K
FarmCPU + K + Q
FarmCPU + K
−log10(p)
Observed −log10(p)
Expected −log10(p)
Chr
1H
2H
3H
4H
5H
6H
7H
SNPs/
1Mb
0
1
8
15
22
29
36
43
50
57
64

MAGIC 1 to 4

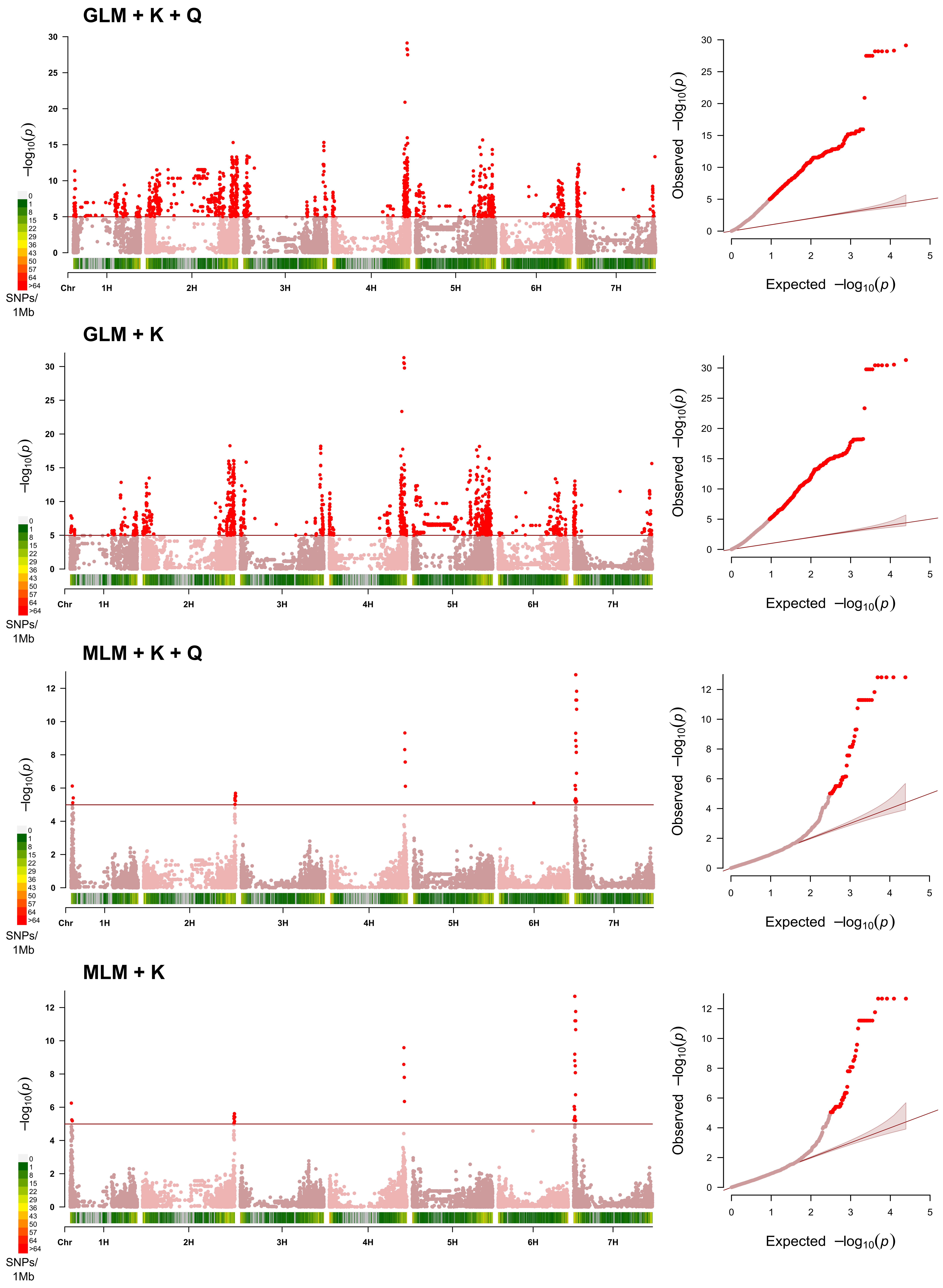
GLM + K + Q
GLM + K
MLM + K + Q
MLM + K
Chr
1H
2H
3H
4H
5H
6H
7H
SNPs/
1Mb
Expected
Observed

MAGIC 1 to 4

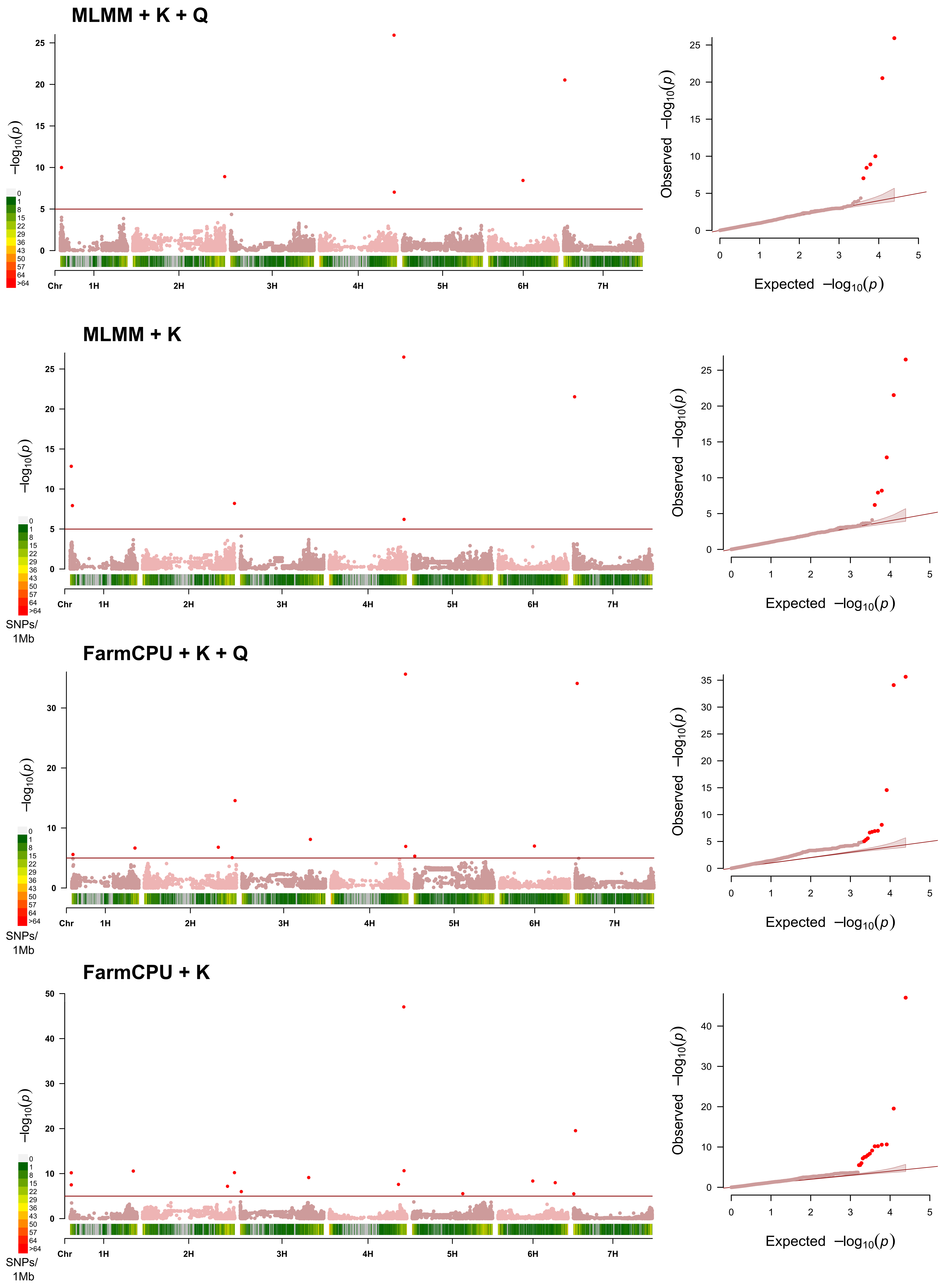

MLMM + K + Q
MLMM + K
FarmCPU + K + Q
FarmCPU + K
−log10(p)
Chr
1H
2H
3H
4H
5H
6H
7H
SNPs/
1Mb
0
1
8
15
22
29
36
43
50
57
64
>64
Observed −log10(p)
Expected −log10(p)
