# Supplementary material for: You Had Me at “MAGIC”!: Four Barley MAGIC Populations Reveal Novel Resistance QTL for Powdery Mildew
Source: Genes (Basel). 2020 Dec 18;11(12):1512. doi: 10.3390/genes11121512 (PMC7766815; doi:10.3390/genes11121512)
Supplement: Supplementary file 1 [file genes-11-01512-s001.zip › Table S1.pdf]

Table S1

You had me at “MAGIC”! Four barley MAGIC populations reveal novel resistance QTL for Powdery Mildew

Fluturë Novakazi [1], Lene Krusell [2], Jens Due Jensen [3], Jihad Orabi [3], The PPP Barley Consortium, Ahmed Jahoor [3], Therése Bengtsson [1*]

[1] Department of Plant Breeding, Swedish University of Agricultural Sciences, Box 101, 230 53, Alnarp Sweden

[2] Sejet Plant Breeding, Nørremarksvej 67, 8700 Horsens, Denmark

[3] Nordic Seed A/S, Kornmarken 1, 8464 Galten Denmark

*Correspondence: Therése Bengtsson: therese.bengtsson@slu.se

Contents

Descriptive statistics for all observations and panels

Correlation plots for all observations and panels

The PPP Barley Consortium
Inger Åhman (Department of Plant Breeding, Swedish University of Agricultural Sciences, Box 101, 230 53 Alnarp, Sweden),
Therése Bengtsson (Department of Plant Breeding, Swedish University of Agricultural Sci-ences, Box 101, 230 53 Alnarp, Sweden),
Fluturë Novakazi (Department of Plant Breeding, Swedish University of Agricultural Scienc-es, Box 101, 230 53 Alnarp, Sweden),
Outi Manninen (Boreal Plant Breeding Ltd, Myllytie 10, 31600 Jokioinen, Finland),
Merja Veteläinen (Boreal Plant Breeding Ltd, Myllytie 10, 31600 Jokioinen, Finland),
Mika Isolahti, (Boreal Plant Breeding Ltd, Myllytie 10, 31600 Jokioinen, Finland),
Muath Alsheikh (Graminor A/S, Hommelstadvegen 60, 2322 Ridabu, Norway),
Stein Bergersen (Graminor A/S, Hommelstadvegen 60, 2322 Ridabu, Norway),
Constantin Jansen (Graminor A/S, Hommelstadvegen 60, 2322 Ridabu, Norway),
Susanne Windju (Graminor A/S, Hommelstadvegen 60, 2322 Ridabu, Norway),
Marja Jalli, (Natural Resources Institute Finland (Luke), Tietotie 4, 31600 Jokioinen, Finland),
Juho Hautsalo, (Natural Resources Institute Finland (Luke), Survontie 9, 40500 Jyväskylä, Finland,
Ahmed Jahoor (NordicSeed A/S, Kornmarken 1, 8464 Galten, Denmark) (project leader),
Jens Due Jensen (Nordic Seed A/S, Kornmarken 1, 8464Galten, Denmark),
Jihad Orabi (Nordic Seed A/S, Kornmarken 1, 8464 Galten, Denmark),
Nana Vagndorf Nordestgaard (Nordic Seed A/S, Kornmarken 1, 8464 Galten, Denmark),
Lene Krusell (Sejet Plant Breeding, Nørremarksvej 67, 8700 Horsens, Denmark),
Rasmus Lund Hjortshøj (Sejet Plant Breeding, Nørremarksvej 67, 8700 Horsens, Denmark),
Charlotte Damgård Robertsen, (Sejet Plant Breeding, Nørremarksvej 67, 8700 Horsens, Den-mark),
Magnus Göransson (The Agricultural University of Iceland, Faculty of Land and Animal Re-sources, Hvanneyri, 311 Borgarnes, Iceland)

Descriptive statistics for all observations and panels, computed with the psych software package v, 1.8.12 (Revelle, 2018) using the R software (R Development Core Team, 2017), Table shows number of observations (n), mean, standard deviation (sd), median, minimum (min), maximum (max), range, skew, kurtosis, and standard error (se).

MAGIC 1+2

Observation	n	mean	sd	median	min	max	range	skew	kurtosis	se
Dyngby_17_1	134	3.09	2.09	3	1	8	7	0.52	-1.05	0.18
Dyngby_17_2	134	2.72	1.77	2	1	7	6	0.54	-1.04	0.15
Dyngby_18_1	134	3.19	1.77	3	1	7	6	0.31	-1.18	0.15
Horsens_18_1	134	2.62	1.65	2	1	6	5	0.48	-1.22	0.14
Horsens_18_2	134	3.93	2.63	4	1	9	8	0.35	-1.24	0.23
All	134	3.11	1.74	3	1	7	6	0.04	-0.87	0.16

MAGIC 3

Observation	n	mean	sd	median	min	max	range	skew	kurtosis	se
Dyngby_17_1	83	4.04	1.74	4	1	7	6	-0.22	-1.03	0.19
Dyngby_17_2	83	3.48	1.71	3	1	7	6	0.22	-0.95	0.19
Dyngby_18_1	83	4.14	1.46	4	1	7	6	0.08	-0.76	0.16
Horsens_18_1	83	3.04	1.31	3	1	6	5	0.29	-0.84	0.14
Horsens_18_2	83	5.47	1.71	6	2	9	7	0.01	-0.65	0.19
All	83	4.03	1.41	4	1	7	6	0.04	-0.87	0.16

MAGIC 4

Observation	n	mean	sd	median	min	max	range	skew	kurtosis	se
Dyngby_17_1	279	4.48	1.58	5	1	7	6	-0.74	-0.27	0.09
Dyngby_17_2	279	3.28	1.28	3	1	6	5	-0.24	-0.65	0.08
Dyngby_18_1	279	4.14	1.18	4	1	7	6	-0.36	-0.12	0.07
Horsens_18_1	279	4.14	1.20	4	1	7	6	-0.52	-0.20	0.07
Horsens_18_2	279	4.17	1.42	4	1	8	7	0.03	-0.51	0.09
All	279	4.04	1.12	4	1	7	6	-0.39	-0.10	0.07

MAGIC 1 to 4

Observation	n	mean	sd	median	min	max	range	skew	kurtosis	se
Dyngby_17_1	490	4.04	1.85	4	1	8	7	-0.35	-1.02	0.08
Dyngby_17_2	490	3.17	1.52	3	1	7	6	0.10	-0.81	0.07
Dyngby_18_1	490	3.90	1.46	4	1	7	6	-0.25	-0.59	0.07
Horsens_18_1	490	3.56	1.51	4	1	7	6	-0.28	-1.00	0.07
Horsens_18_2	490	4.33	1.94	4	1	9	8	0.13	-0.51	0.09
All	490	3.80	1.41	4	1	7	6	-0.27	-0.61	0.06

Analysis of variance (ANOVA model III, with Satterthwaite's method) of powdery mildew using lmer function in R, Genotypes, environments, and genotype by environment interactions were considered, as fixed effects, and the observations nested within the environments, as random effect

**MAGIC 1+2**	Sum of Squares	Mean Squares	Degree of Freedom	F-value	Pr(>F)
GEN	1816	13.66	133	7.25	<0.0001
ENV	0.59	0.29	2	0.16	0.8647
GEN*ENV	190	0.71	266	0.38	1

Residual estimate = 1.8831, and Residual standard error = 1.3723

**MAGIC 3**	Sum of Squares	Mean Squares	Degree of Freedom	F-value	Pr(>F)
GEN	741	9.04	82	9.9231	<0.0001
ENV	0.15	0.076	2	0.0832	0.9232
GEN*ENV	74.07	0.45	164	0.4956	1

Residual estimate = 0.9113, and Residual standard error = 0.9546

**MAGIC 4**	Sum of Squares	Mean Squares	Degree of Freedom	F-value	Pr(>F)
GEN	1579	5.68	278	6.8812	<0.0001
ENV	0.20	0.10	2	0.1205	0.8925
GEN*ENV	312	0.56	556	0.680	1

Residual estimate = 0.8253, and Residual standard error = 0.9085

**MAGIC 1 to 4**	Sum of Squares	Mean Squares	Degree of Freedom	F-value	Pr(>F)
GEN	4429	9.06	489	6.544	<0.0001
ENV	0.50	0.25	2	0.1820	0.8460
GEN*ENV	575	0.59	978	0.425	1

Residual estimate=1.3841, and Residual standard error= 1.1765
